# Supplementary material for: Proteomic and genetic analyses of influenza A viruses identify pan-viral host targets
Source: Nat Commun. 2023 Sep 27;14:6030. doi: 10.1038/s41467-023-41442-z (PMC10533562; doi:10.1038/s41467-023-41442-z)
Supplement: Supplementary file 1 — Supplementary Information File [file 41467_2023_41442_MOESM1_ESM.pdf]

Supplementary Information:

Proteomic and Genetic Analyses of Influenza A Viruses Identify Pan-Viral Host Targets

Haas *et al.*

Supplementary Information

1. Supplementary Figures and Figure Legends 1-7

2. Supplementary References

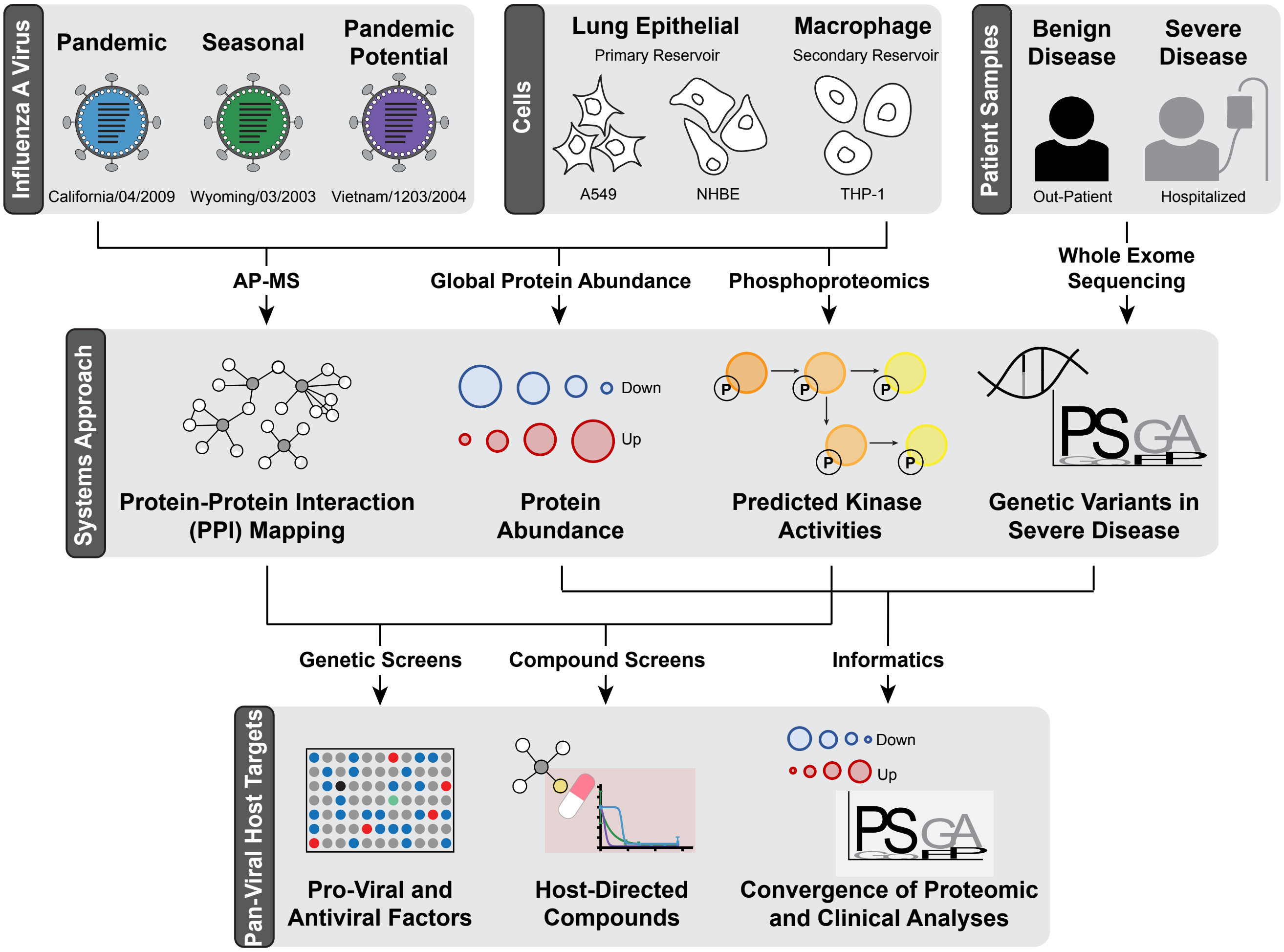

Supplementary Figure 1. An Integrative Systems Biology Approach Functionally Characterizes Pan-Influenza A Virus (IAV)-Human Protein Interactions and Signaling Networks. An integrative systems biology approach studying three IAV strains that impact human health, including 2009 swine flu pandemic strain pH1N1, seasonal-circulating strain H3N2 and pandemic-potential strain H5N1, in three human cell types relevant for IAV infection and in an influenza patient cohort. Using global approaches, we mapped IAV-human protein-protein interactions (PPIs) by affinity purification-mass spectrometry (AP-MS) and generated an IAV-human interactome. By global proteomic profiling, we quantified changes in global protein abundance and phosphorylation and identified kinases with IAV-modulated changes in activity. From a cohort of patients with benign or severe influenza disease, whole exome sequencing revealed genes with pLOF variants associated with severe influenza disease. Host factors identified in the systems approaches were functionally interrogated to identify pan-viral host targets. siRNA knockdown of human proteins from PPI and PH approaches revealed pro-viral and antiviral factors of IAV infection. Compounds targeting PPI and PH factors were screened against pH1N1, H3N2 and H5N1 IAV infection, as well as SARS-CoV-2 infection, which identified compounds with antiviral activity across influenza and coronavirus families. Bioinformatic analyses identified host factors at the convergence of proteomic and patient datasets. Functional IAV host factors identified in this study represent putative targets for future potential pan-viral HDT.

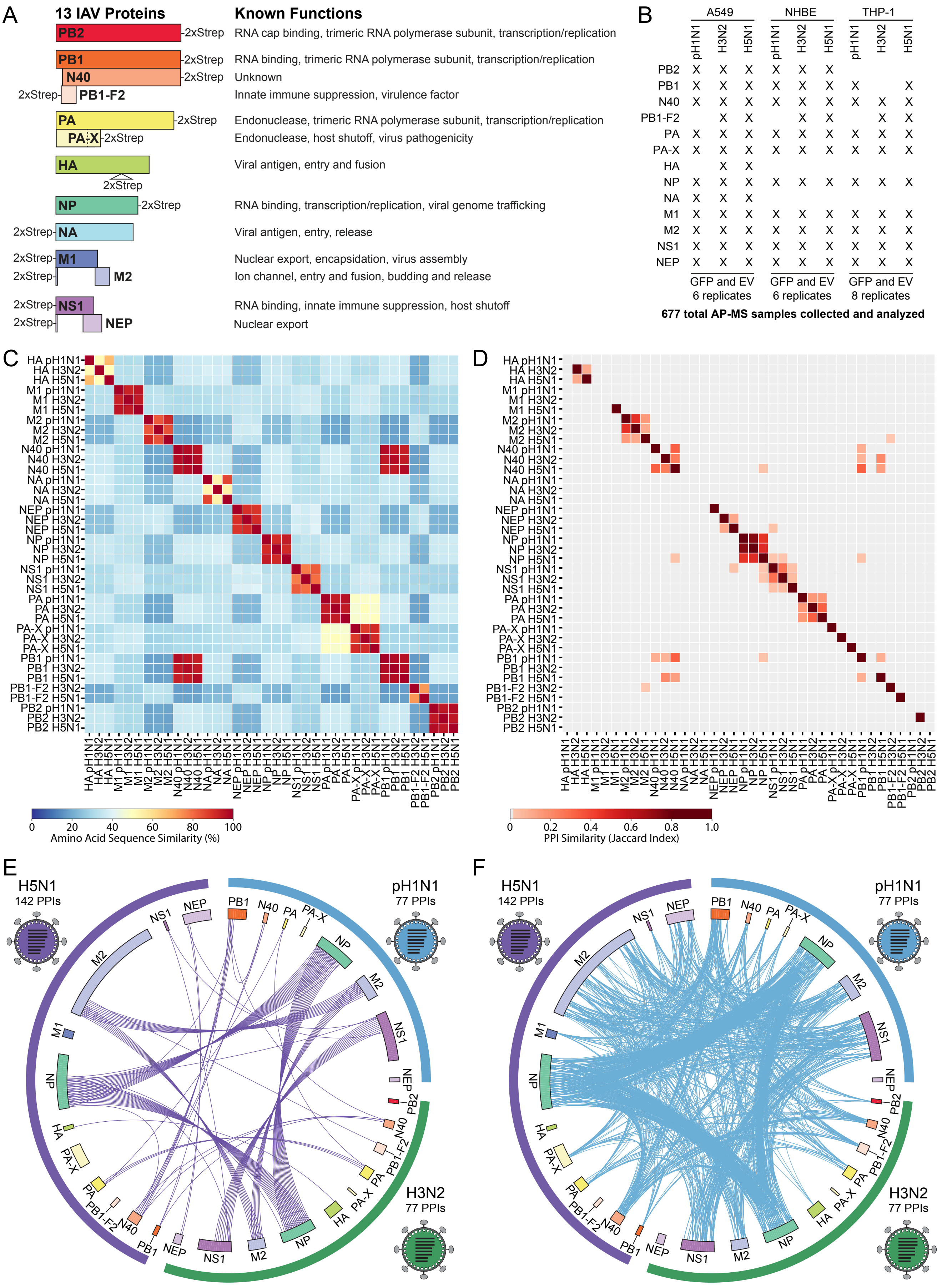

Supplementary Figure 2. Summary of IAV AP-MS Data. A. Schematic of the 13 IAV proteins with N-

terminal, internal or C-terminal 2X-Strep tag (left) and their known biological functions (right), drawn to scale and grouped by genomic RNA segment to show protein products from each segment. IAV proteins include: virus surface proteins (HA, NA) and membrane-embedded ion channel (M2) involved in virus fusion and entry (HA, M2) and virus budding and release (M2, NA); an RNA-binding protein involved in viral genome trafficking (NP); proteins involved in viral genome transcription and replication (trimeric RNA-dependent RNA polymerase subunits PA, PB1 and PB2); proteins that facilitate viral RNA export from the nucleus (NEP, M1) and encapsidation of viral RNA during virus assembly (M1); proteins that modulate host immune response, host shutoff, virus pathogenicity and virulence (NS1, PA-X, PB1-F2); and a protein needed for efficient replication but whose cellular function is not well-characterized (N40)[1,2,3]. **B.** AP-MS samples successfully collected and analyzed across the 13 IAV proteins from three IAV strains in three cell types (marked as X). pH1N1 does not express PB1-F2[4], therefore no samples were generated. GFP and empty vector (EV) control samples were also collected in each cell type. Number of replicates for each 2X-Strep-tagged IAV and control protein in each cell type are listed. **C.** Heatmap comparing percent amino acid sequence similarity across the total 38 IAV proteins (12 from pH1N1 which excludes PB1-F2, 13 from H3N2 and 13 from H5N1). **D.** Heatmap comparing PPI similarity expressed as Jaccard index for human proteins interacting with the total 38 IAV proteins. **E-F.** Circos plots representing the human interacting proteins shared between IAV proteins of the three strains (**E**, purple lines) or biological pathways of the human interacting proteins shared between IAV proteins of the three strains (**F**, blue lines). Inner circle depicts the IAV proteins (bars for each IAV protein colored as in (**A**) and scaled to the number of interactions). Outer circle depicts the IAV strain (pH1N1 blue, H3N2 green, H5N1 purple; bar scaled to the number of interactions). Circos plots were generated using Metascape[5].

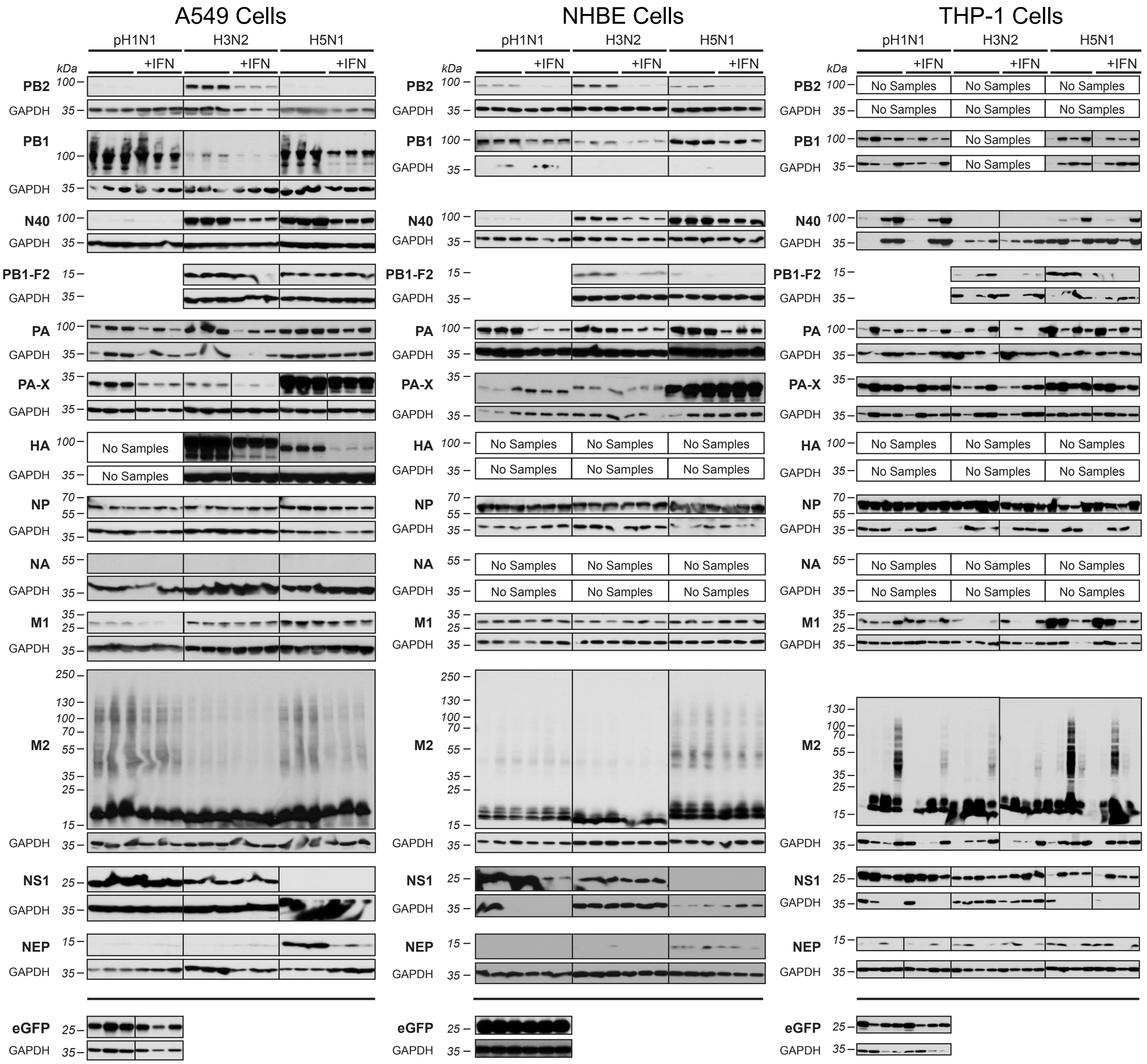

Supplementary Figure 3. IAV Protein Expression. Western blots probing against the 2X-Strep tag to assay IAV protein expression in three cell types. pH1N1 does not express PB1-F2[4], therefore no samples were generated. Samples that had cell toxicity upon IAV protein expression or no IAV protein expression were not collected for MS analysis (marked as No Samples). Across the three cell lines, these include: pH1N1 HA 2X-Strep (A549 cells); HA 2X-Strep from all strains and NA 2X-Strep from all strains (NHBE cells); and PB2 2X-Strep from all strains, H3N2 PB1 2X-Strep, HA 2X-Strep from all strains, and NA 2X-Strep from all strains (PMA-differentiated THP-1 cells). GAPDH loading controls were probed on the same blot for each IAV protein (bottom row, paired with each IAV protein). Cells were untreated or treated with universal type I interferon (+IFN), however there were few discernible differences in observed PPIs between untreated and treated samples, therefore replicate sets were combined totaling six replicates (A549 and NHBE) or eight replicates (THP-1) to increase statistical power (see also **Methods**). Sets of adjacent gel lanes are outlined by black boxes and arranged by strain and cell treatment; borders demarcate non-adjacent lanes. Uncropped Western blot scans are included as Source Data.

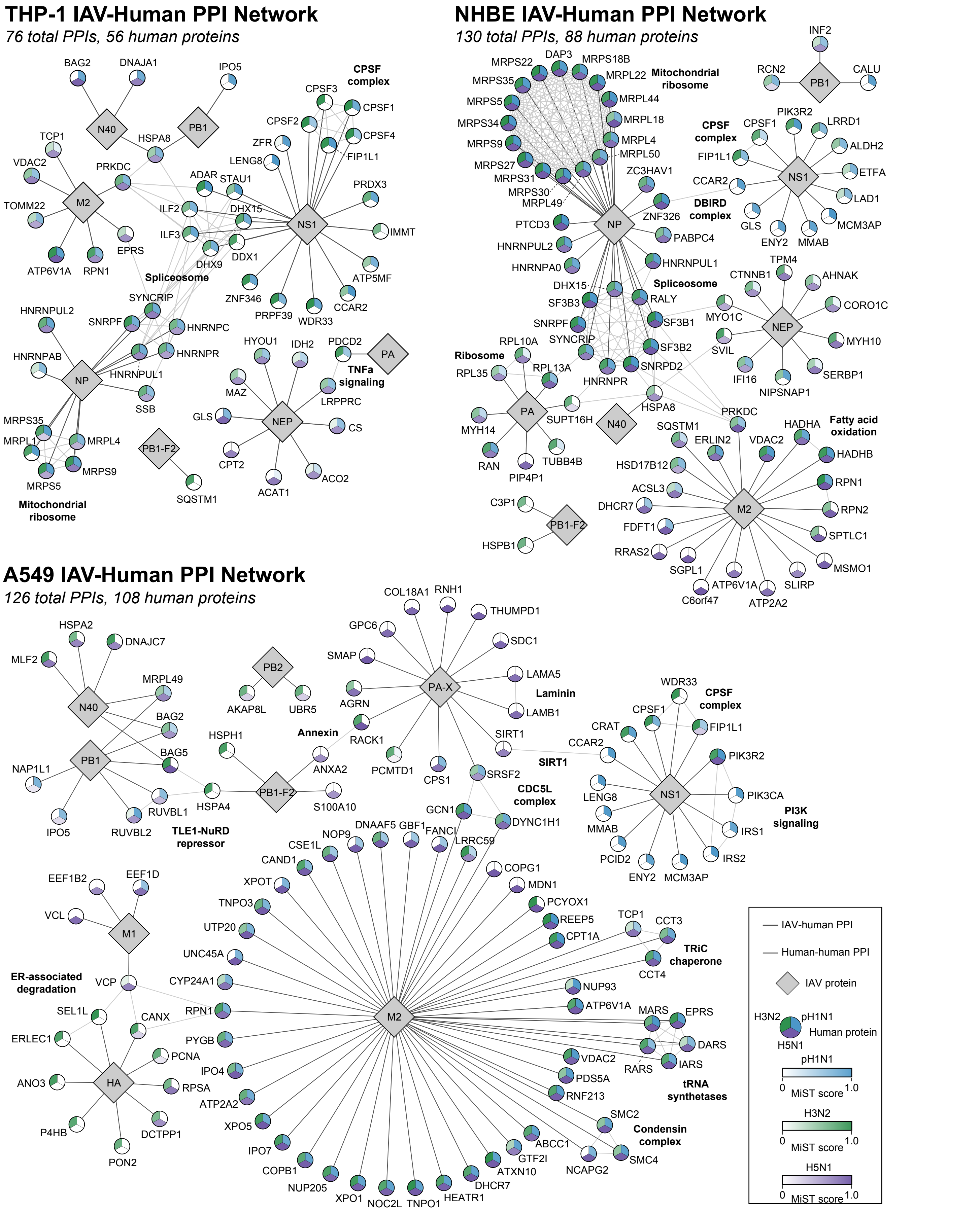

Supplementary Figure 4. PPI Networks Specific to Each Cell Type. PPI networks specific to each cell type.

76 total high-confidence IAV-human PPIs across all strains in PMA-differentiated THP-1 cells are mapped between eight IAV proteins and 56 human proteins (top, left). 130 total high-confidence IAV-human PPIs across all strains in NHBE cells are mapped between eight IAV proteins and 88 human proteins (top, right). 126 total high-confidence IAV-human PPIs across all strains in A549 cells are mapped between nine IAV proteins and 108 human proteins (bottom). IAV protein nodes and human protein nodes are colored as described in **Fig2**. Human-human PPIs are identified as curated in CORUM[6] and labeled as described in **Fig2**.

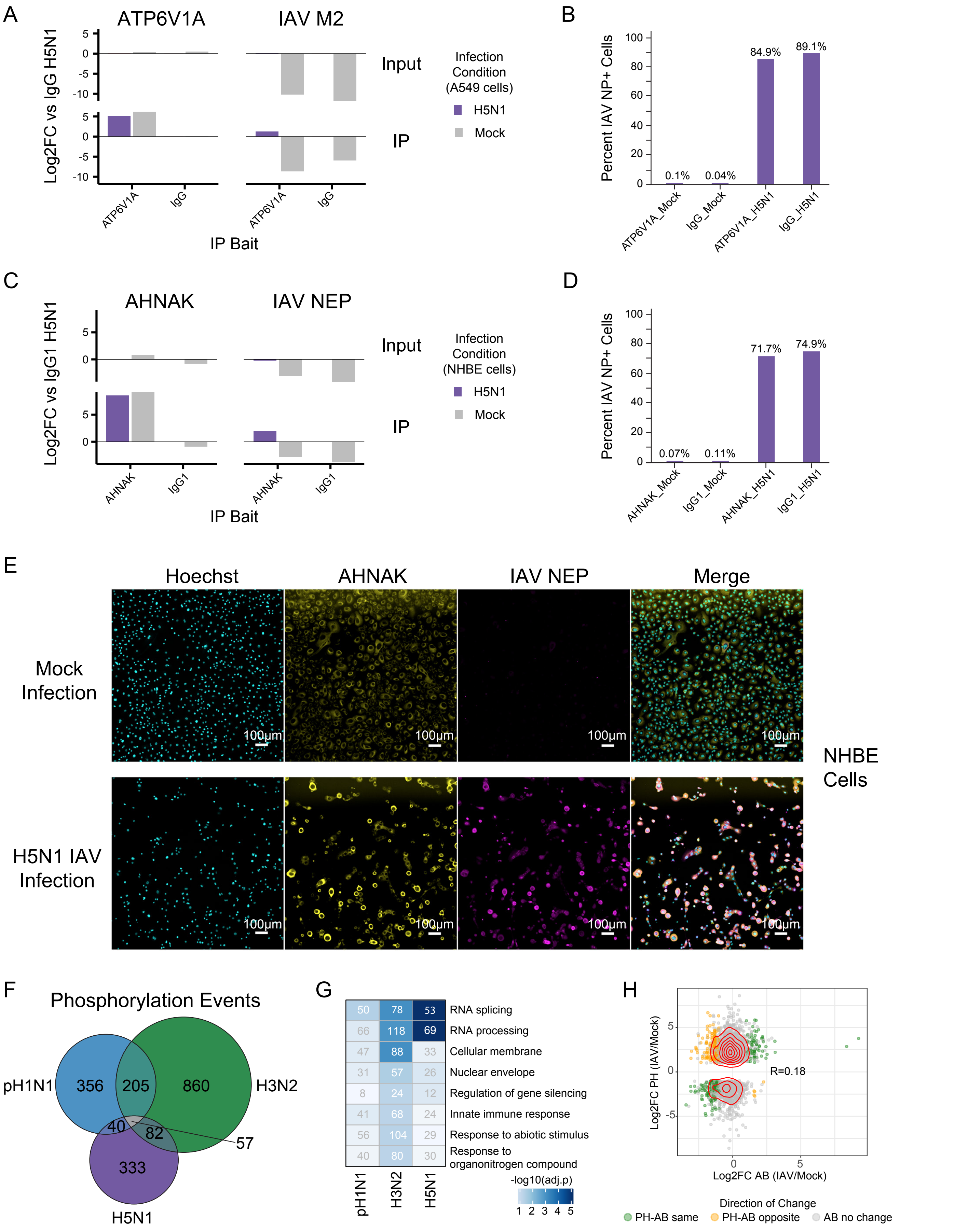

Supplementary Figure 5. Validation of ATP6V1A and AHNAK PPIs in H5N1-Infected Cells, and PH and

AB Dataset Analyses. A and C. Input and immunoprecipitation (IP) protein intensity measured by targeted MS of (**A**) ATP6V1A and IAV M2 (Log2FC experimental/IgG from H5N1-infected A549 cells), and (**C**) AHNAK and IAV NEP (Log2FC experimental/IgG1 from H5N1-infected NHBE cells). A549 cells or NHBE cells were mock-infected or H5N1 IAV-infected (MOI 0.5, 24hr) prior to endogenous pulldown. **B and D.** Percent IAV NP-positive (NP+) cells quantified from (**B**) ATP6V1A and IgG pulldowns from A549 cells in (**A**), or (**D**) AHNAK and IgG1 pulldowns from NHBE cells in (**C**). Flow cytometry data was analyzed using the gating strategy in **Supplementary Figure 6A**. **E.** Immunofluorescence staining against cell nuclei (Hoechst, cyan), AHNAK (yellow) and IAV NEP (magenta) in NHBE cells that were mock-infected or H5N1 IAV-infected (MOI 0.5, 24 hours; performed in n=3 biologically independent samples; images from n=1 representative replicate). **F.** Venn diagram of the total number of significantly changing phosphorylation events at 18 hours post-infection (pH1N1, H3N2) and 12 hours post-infection (H5N1), unified across both cell types. **G.** Heatmap of GO enrichments of the PH data at 18 hours post-infection (pH1N1, H3N2) and 12 hours post-infection (H5N1), unified across cell types. Increasing shading intensity reflects increasing significance of the enrichment term. Significant GO terms were defined as those with adjusted p-value < 0.05, and non-redundant terms were selected by automated clustering procedure (see also **Methods**). The number of proteins per enriched cluster are shown in white if significant (adjusted p-value < 0.05), and grey if not significant (adjusted p-value > 0.05). **H.** Scatterplot of all significant log2FC in protein phosphorylation (Log2FC PH (IAV/Mock)) and corresponding log2FC in protein abundance (Log2FC AB (IAV/Mock)) in IAV-infected cells compared to mock-infected controls. Data points are colored as described in **Fig3E**. Each data point is an individual phosphosite or combination of phosphosites when multiple phosphorylations were observed within single peptides, and all significantly changing sites from the same protein were correlated against that protein's Log2FC in AB.

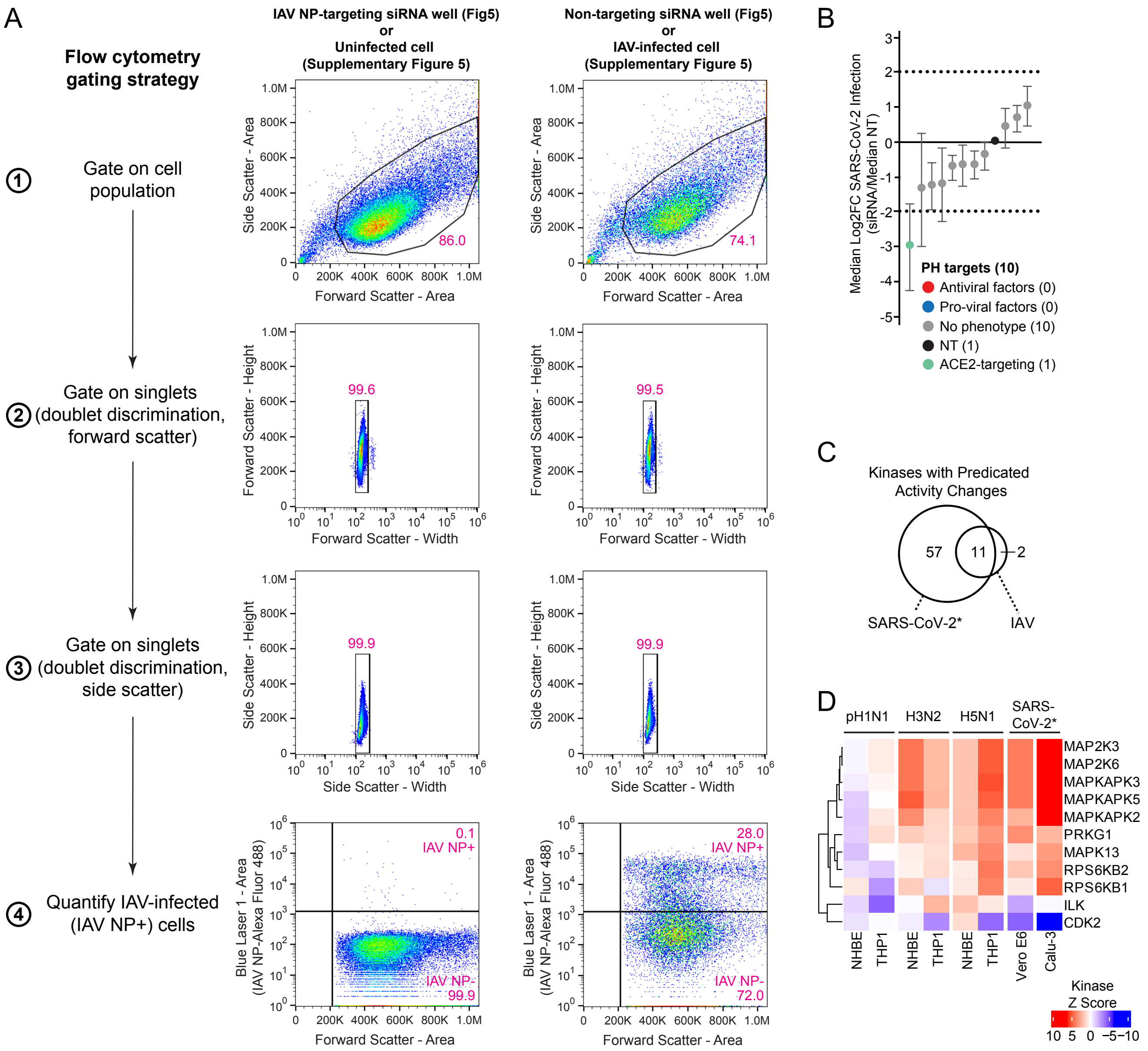

Supplementary Figure 6. Probing IAV Targets against SARS-CoV-2 Infection Identifies Factors that Regulate both IAV and SARS-CoV-2. A. Flow cytometry gating strategy to quantify percent IAV-infected (%IAV NP+) A549 or NHBE cells, using representative flow wells (cell samples) from the siRNA screen for IAV NP-targeting siRNA and non-targeting siRNA controls. **B.** Distribution of log2 fold changes in SARS-CoV-2 infection for siRNA knockdown of 10 PH targets (from **Fig5E**) compared to NT siRNA (black dot), plotted as the median of nine replicates (n=3 biologically independent samples, each in n=3 technical replicates) per target. The log2 fold change in SARS-CoV-2 infection was calculated for each experimental siRNA against a replicate-matched NT siRNA (**Methods**). siRNA with median log2 fold change ≤ -2 were labeled pro-viral factors (blue dots) and siRNA with median log2 fold change ≥ 2 were labeled antiviral factors (red dots). siRNA in between these thresholds were labeled no/weak phenotype (grey dots). The median log2 fold change of positive-control ACE2-targeting siRNA is represented (green dot). Error bars represent median absolute deviations (MAD). **C.** Venn diagram comparing kinases with significant activity changes during IAV infection (this study) and during SARS-CoV-2 infection[7] (asterisk signifies data was derived from[7]). **D.** Heatmap of kinase activity predictions from the phosphorylation data with IAV infection (at 18 hours post-infection (pH1N1, H3N2) and 12 hours post-infection (H5N1); in NHBE and THP-1 cells) and SARS-CoV-2 infection (at 24 hours post-infection; in Vero E6 and Calu-3 cells) from published studies[7,8,9] and thresholded at p-value < 0.05 (asterisk signifies data was derived from[7,8,9]). Kinase Z-score reflects predicted kinase activity, with increased kinase activity in red and decreased kinase activity in blue.

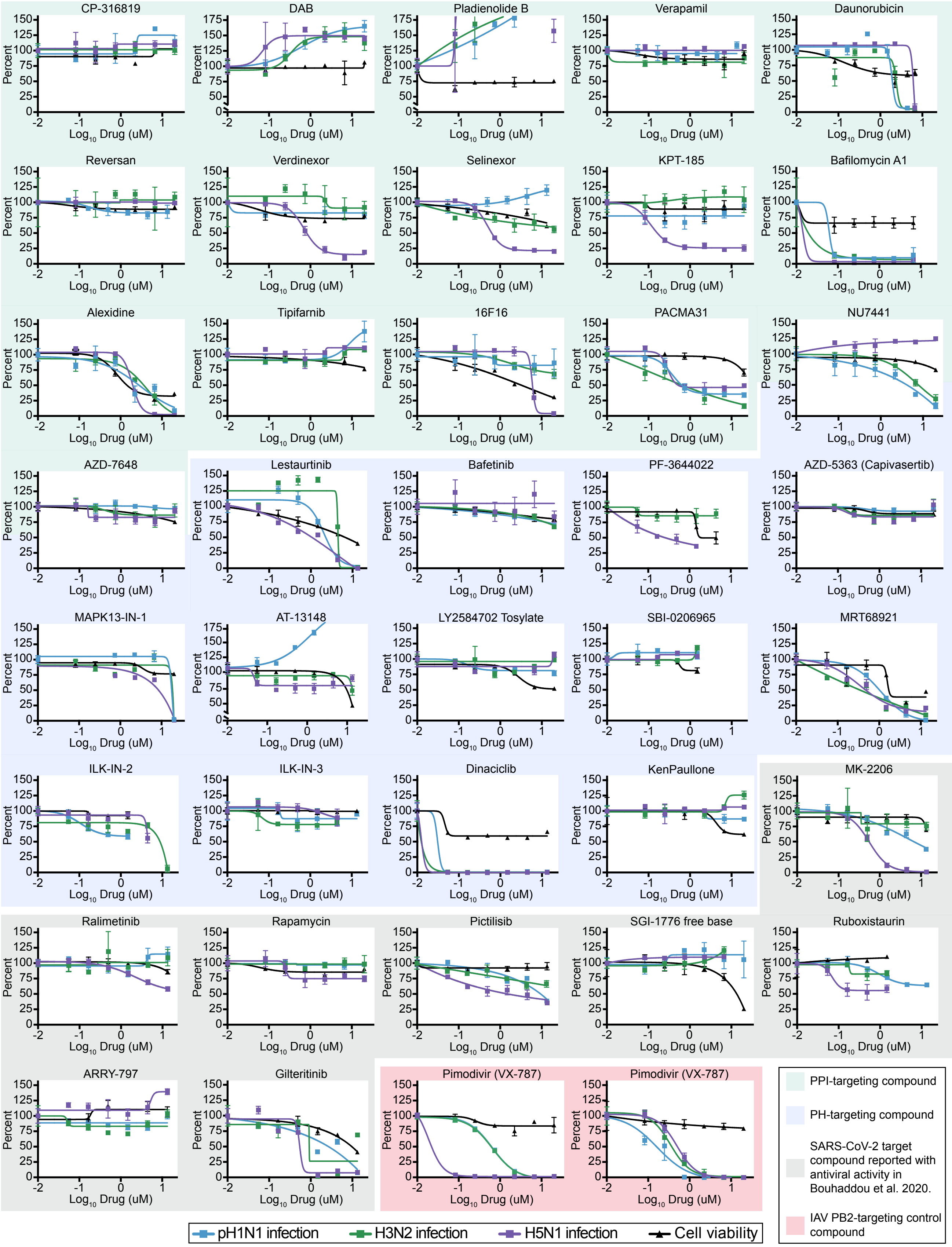

Supplementary Figure 7. Host-Directed Compound Dose-Response Curves for pH1N1, H3N2 and H5N1 IAV and Cell Viability. Dose-response curves for a total of 37 host-directed compounds, including: 16 IAV PPI-targeting drugs (green background), 15 kinase-targeting drugs (blue background), and 8 kinase-targeting

drugs mined from a published study with antiviral activity against SARS-CoV-2[7] (grey background). pH1N1 was not screened with PF-3644022. Dose-response curves are also included for a control compound, IAV PB2-targeting Pimodivir (VX-787), which was run in two sets (red background). Assays were performed in n=3 biologically independent samples in A549 cells, with high throughput imaging and quantification of percent IAV-infected cells (%NP+ cells) for each of the three IAV strains (blue line=pH1N1; green line=H3N2; purple line=H5N1), and percent viable cells (black line). For each data point, the mean of three replicates is shown. Error bars represent standard error of mean (SEM). Graph data points are also provided as Source Data.

Supplementary References

1. Wise, H. M. *et al.* A complicated message: Identification of a novel PB1-related protein translated from influenza A virus segment 2 mRNA. *J. Virol.* **83**, 8021–8031 (2009).
2. Dou, D., Revol, R., Östbye, H., Wang, H. & Daniels, R. Influenza A Virus Cell Entry, Replication, Virion Assembly and Movement. *Front. Immunol.* **9**, 1581 (2018).
3. Klemm, C., Boergeling, Y., Ludwig, S. & Ehrhardt, C. Immunomodulatory Nonstructural Proteins of Influenza A Viruses. *Trends Microbiol.* **26**, 624–636 (2018).
4. Garten, R. J. *et al.* Antigenic and genetic characteristics of swine-origin 2009 A(H1N1) influenza viruses circulating in humans. *Science* **325**, 197–201 (2009).
5. Zhou Y. *et al*. Metascape provides a biologist-oriented resource for the analysis of systems-level datasets. *Nat. Commun.* **10**, 1523 (2019).
6. Giurgiu, M. *et al.* CORUM: the comprehensive resource of mammalian protein complexes-2019. *Nucleic Acids Res.* **47**, D559–D563 (2019).
7. Bouhaddou, M. *et al.* The Global Phosphorylation Landscape of SARS-CoV-2 Infection. *Cell* **182**, 685–712.e19 (2020).
8. Thorne, L. G. *et al.* Evolution of enhanced innate immune evasion by SARS-CoV-2. *Nature* **602**, 487–495 (2022).
9. Thorne, L. G. *et al.* Publisher Correction: Evolution of enhanced innate immune evasion by SARS-CoV-2. *Nature* **604**, E14 (2022).
