# Supplementary material for: Proteomic and genetic analyses of influenza A viruses identify pan-viral host targets
Source: Nat Commun. 2023 Sep 27;14:6030. doi: 10.1038/s41467-023-41442-z (PMC10533562; doi:10.1038/s41467-023-41442-z)
Supplement: Supplementary file 3 — Description of Additional Supplementary Files [file 41467_2023_41442_MOESM3_ESM.docx]

##### **Description of Additional Supplementary Files**

**Supplementary Data 1**

Title: AP-MS IAV Protein Sequences, Scored IAV-Human PPIs, Interacting Proteins across Published Studies, PPI Enrichments and PRM Data, Related to Figures 1-2 and Supplementary Figures 2-5.

Description: Sequences of IAV and control proteins for AP-MS, including tag location, codon-optimization, sequence notes, and full protein and DNA sequences for proteins encoded by pH1N1, H3N2 and H5N1 IAV (Protein_Sequences_for_APMS tab). Full list of MiST-scored PPIs (Full_PPI_List tab) and thresholded high-confidence MiST-scored PPIs (Filtered_PPI_List tab) between 13 proteins from three IAV strains and human proteins in three cell types. Scoring thresholds are described in **Methods**. Column descriptions for PPI lists are provided (PPI_List_Column_Descriptions tab). We report the human interacting proteins that were novel to our AP-MS study and published in other studies that used AP-MS with affinity-tagged IAV proteins exogenously expressed in cell lines^28,30^, AP-MS in the context of virus infection^24,29^, and an orthologous yeast two-hybrid approach^34^ (InteractingProteins_By_Study tab). Gene Ontology (GO) molecular function (MF) enrichments for PPIs are reported for each IAV protein unified across all strains and cell types, with the full enrichment list (PPI_GO_MFfull tab) and with heatmap selection criteria of p-value < 0.002 and top 3 non-redundant terms with at least 2 genes for at least one IAV protein (see also **Methods**) (PPI_GO_MFselection tab). Column descriptions for GO MF lists are provided (GO_List_Column_Descriptions tab). PRM data are provided from endogenous ATP6V1A or AHNAK pulldowns quantifying co-purified IAV M2 or NEP, respectively (PRM_ATP6V1A_Log2FC and PRM_AHNAK_Log2FC tabs).

**Supplementary Data 2**

Title: Global Protein Abundance and Phosphorylation Data, Phosphorylation GO Enrichments and Predicted Kinase Activities, Related to Figure 3 and Supplementary Figure 5.

Description: Full list of global protein abundance measurements (AB_Full tab), list of significant protein abundance measurements (AB_Sig tab), full list of phosphorylation site measurements (PH_Full tab), and list of significant phosphorylation site measurements (PH_Sig tab) with pH1N1, H3N2 and H5N1 IAV infection in NHBE and THP-1 cells. All tabs report the log2 fold change in protein abundance or phosphorylation at the specified site, as well as adjusted p-value. Full lists report measurements from all time points (3hr, 6hr, 12hr, 18hr). Significant lists report measurements at 18 hours post-infection (pH1N1, H3N2) and 12 hours post-infection (H5N1), with selection criteria of: (1) adjusted p-value < 0.05; and (2) absolute(log2FC) > 1 (see also **Methods**). Empty data cells represent no protein detected at the indicated time point, IAV strain or cell type. Column descriptions for all AB and PH tabs are reported (ABPH_Column_Descriptions). GO enrichments for significantly changing phosphorylation sites are reported at 18 hours post-infection (pH1N1, H3N2) and 12 hours post-infection (H5N1) in NHBE and THP-1 cells with the full enrichment list (PH_GO_Full tab) and with heatmap selection criteria of adjusted p-value < 0.05 and automated clustering to select non-redundant terms (see also **Methods**) (PH_GO_Selection tab). Column descriptions for GO lists are provided (GO_List_Column_Descriptions tab). Finally, a full list of predicted kinase activities at each time point post-infection with pH1N1, H3N2 or H5N1 IAV in NHBE and THP-1 cells is reported (Predicted_Kinase_Activity tab). Column descriptions for Kinase Activity are provided (Kinase_Column_Descriptions tab).

**Supplementary Data 3**

Title: Identification of Rare Gene Variants in Patients with Severe Influenza Disease, Related to Figure 4.

Description: Detailed mutation test reports showing gene variant site locations and the results from six pLOF tests for genes identified in patient samples that correspond to proteins identified in the AB, PH and PPI datasets (List_of_pLOF_variants_ABgenes, List_of_pLOF_variants_PHgenes, and List_of_pLOF_variants_PPIgenes tabs). Association test results of hospitalized patients vs non-hospitalized patients, reporting the fraction of individuals carrying pLOF, the number of variants tested for pLOF, the number of singletons, and association tests, are provided for genes that correspond to proteins identified in the AB, PH and PPI datasets (Association_test_ABgenes, Association_test_PHgenes, and Association_test_PPIgenes tabs). Lastly, all pLOF variants from the PH dataset that have phosphorylation disruption mutations sites, including pLOF and phosphorylation site plus any existing data from PhosphoSitePlus, are provided for genes that correspond to proteins identified in the AB, PH and PPI datasets (List_of_pLOF_and_PHsite_ABgenes, List_of_pLOF_and_PHsite_PHgenes, and List_of_pLOF_and_PHsite_PPIgenes tabs).

**Supplementary Data 4**

Title: siRNA Targets and Log2 Fold Change in IAV and SARS-CoV-2 Infection, Related to Figure 5 and Supplementary Figure 6.

Description: For the IAV screen, this table reports a list of siRNA target genes from the IAV PPI and PH datasets with corresponding cell viability (IAVsiRNA_CellViability tab) and the log2 fold change in IAV infection (IAVsiRNA_Infectivity tab). For the SARS-CoV-2 screen, this table reports a list of siRNA target genes with corresponding cell viability (SARS2siRNA_CellViability tab) and the log2 fold change in SARS-CoV-2 infection (SARS2siRNA_Infectivity tab).

**Supplementary Data 5**

Title: Compound Screening against IAV Infection, Related to Figure 6 and Supplementary Figure 7.

Description: Table listing the 37 host-directed compounds screened in this study (Compounds tab), including: 29 IAV PPI- and kinase-targeting compounds; and 8 kinase-targeting compounds that have antiviral activity against SARS-CoV-2^19^. Table reports the compound’s designated protein target and maps the protein target to the dataset in which it was identified: the functionally validated IAV PPI dataset (**Fig5G**), IAV kinase analysis (**Fig3F**), or from a SARS-CoV-2 global PH study that reports compounds with antiviral activity against SARS-CoV-2^19^. Also reported are each compound’s U.S. approval status, supplier, catalog number, PubChem ID, IC50 and IC90 (IAV inhibition), CC10 and CC50 (cell viability), and selectivity index (SI). Compounds that were screened and do not have IAV inhibition (IC50 or IC90) or cell toxicity (CC10 or CC50) values as determined by nonlinear regression fit and fit hill functions are reported as > 20uM. For compounds where CC50 is reported as > 20uM, SI was calculated and reported as > (20uM/IC50). For compounds where CC50 and IC50 are reported as > 20uM, SI is reported as N/A. Column descriptions are provided (Column_Descriptions tab).

**Supplementary Data 6**

Title: Liquid Chromatography (LC) and Mass Spectrometry (MS) Acquisition Parameters, Related to Methods.

Description: LC and MS acquisition parameters are reported for the three instruments that acquired proteomic data in our study. A table of contents is included matching each instrument to their respective LC and MS parameters tabs and to the proteomic data collected on each instrument, including data type, cell type and IAV strain.
