# Supplementary figures and images for: Proteomic and genetic analyses of influenza A viruses identify pan-viral host targets
Source: Nat Commun. 2023 Sep 27;14:6030. doi: 10.1038/s41467-023-41442-z (PMC10533562; doi:10.1038/s41467-023-41442-z)

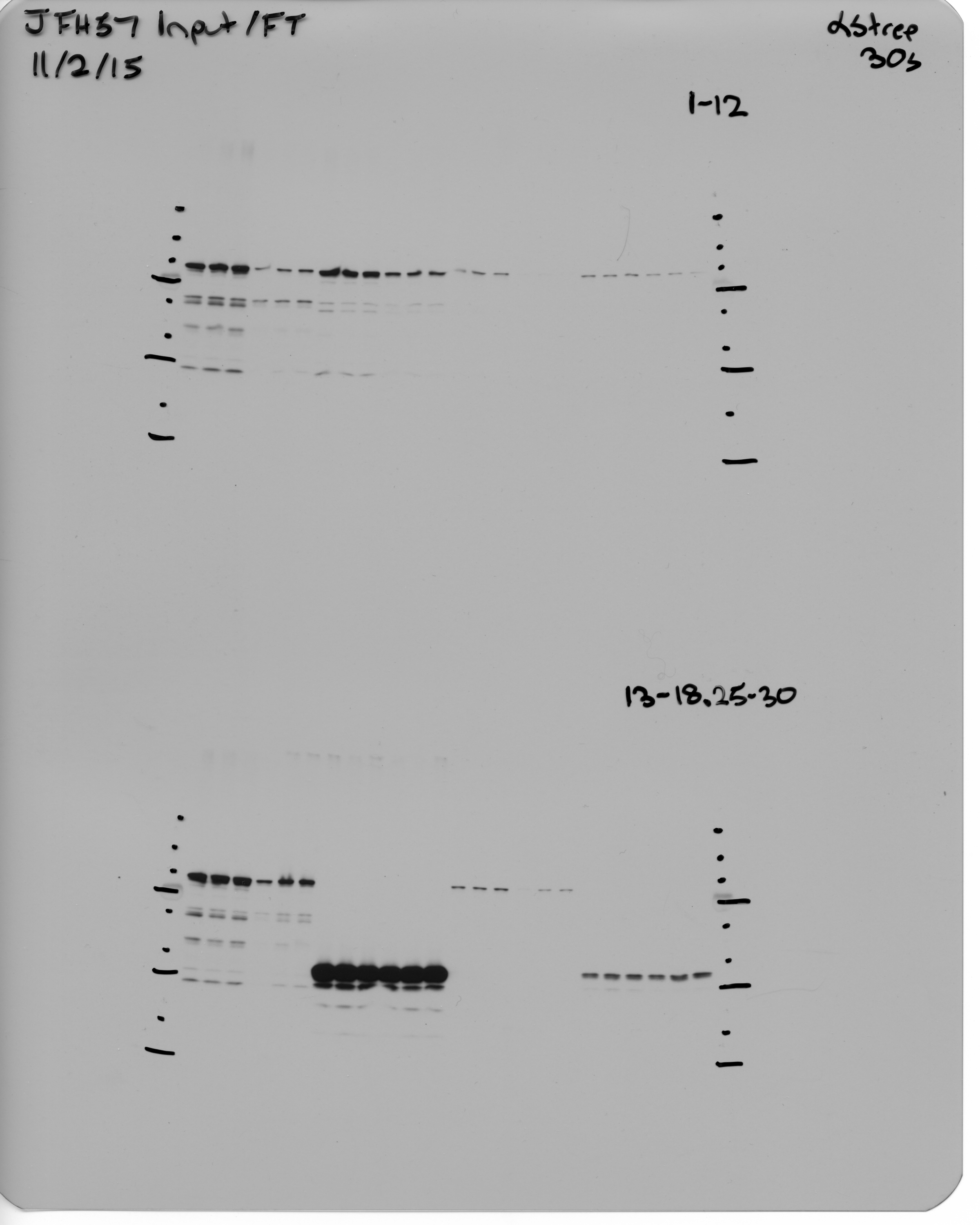

Supplement: Supplementary file 11 — Source Data [file 41467_2023_41442_MOESM11_ESM.zip › Haas_SourceData/Western Blot Scans (Supp Fig 3)/NHBE/Strep - 001.tif]

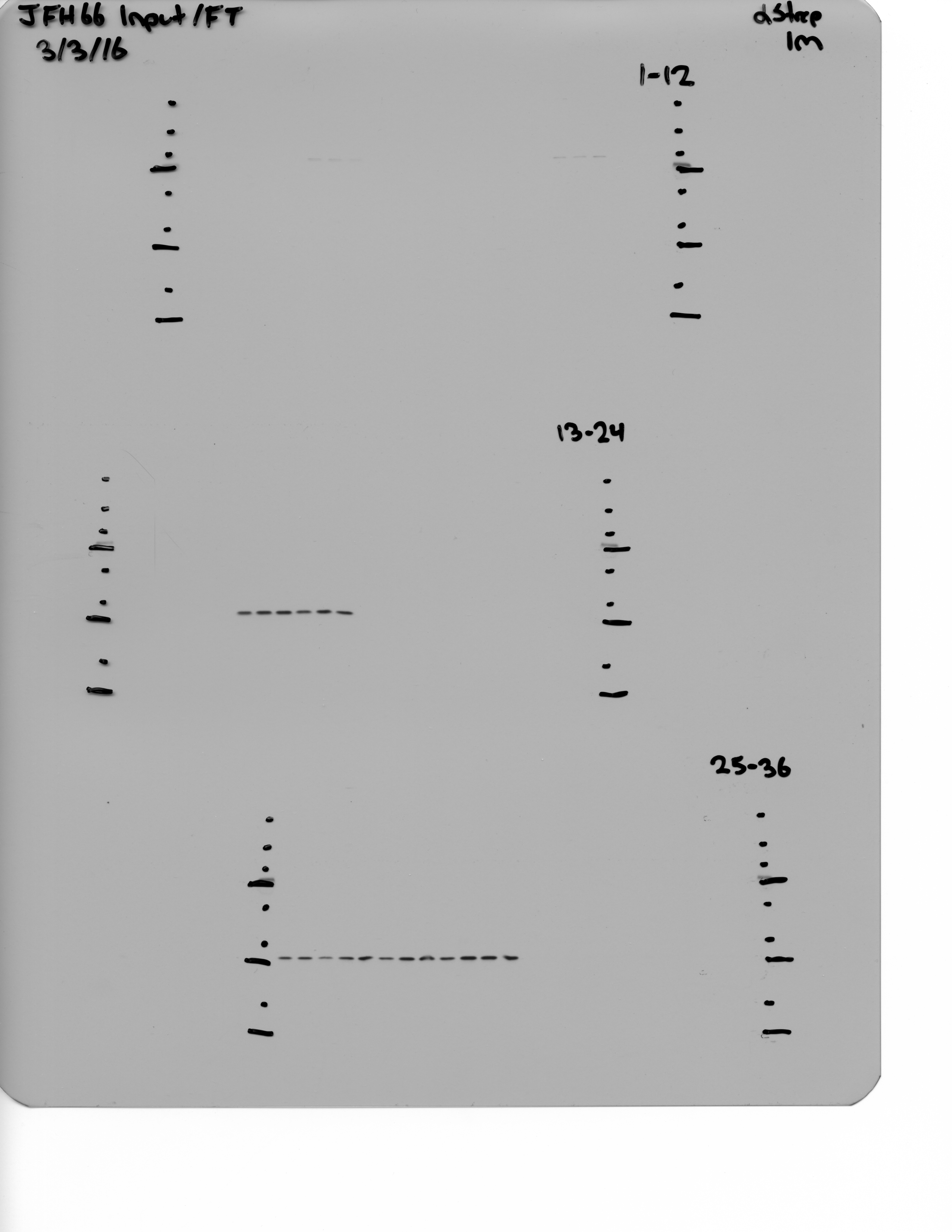

Supplement: Supplementary file 11 — Source Data [file 41467_2023_41442_MOESM11_ESM.zip › Haas_SourceData/Western Blot Scans (Supp Fig 3)/NHBE/JFH066 - Strep - 1m.tif]

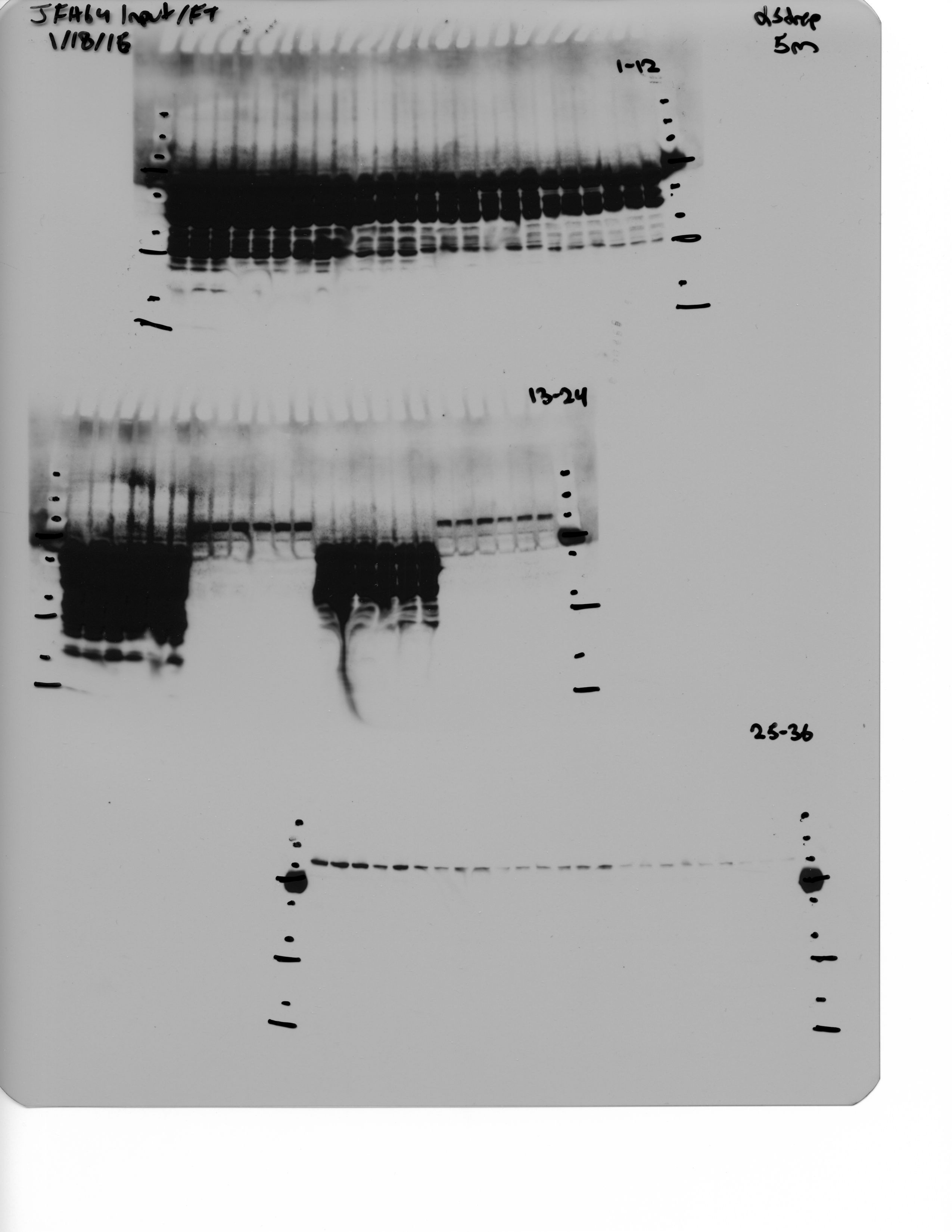

Supplement: Supplementary file 11 — Source Data [file 41467_2023_41442_MOESM11_ESM.zip › Haas_SourceData/Western Blot Scans (Supp Fig 3)/NHBE/JFH064 - Strep - 5m.tif]

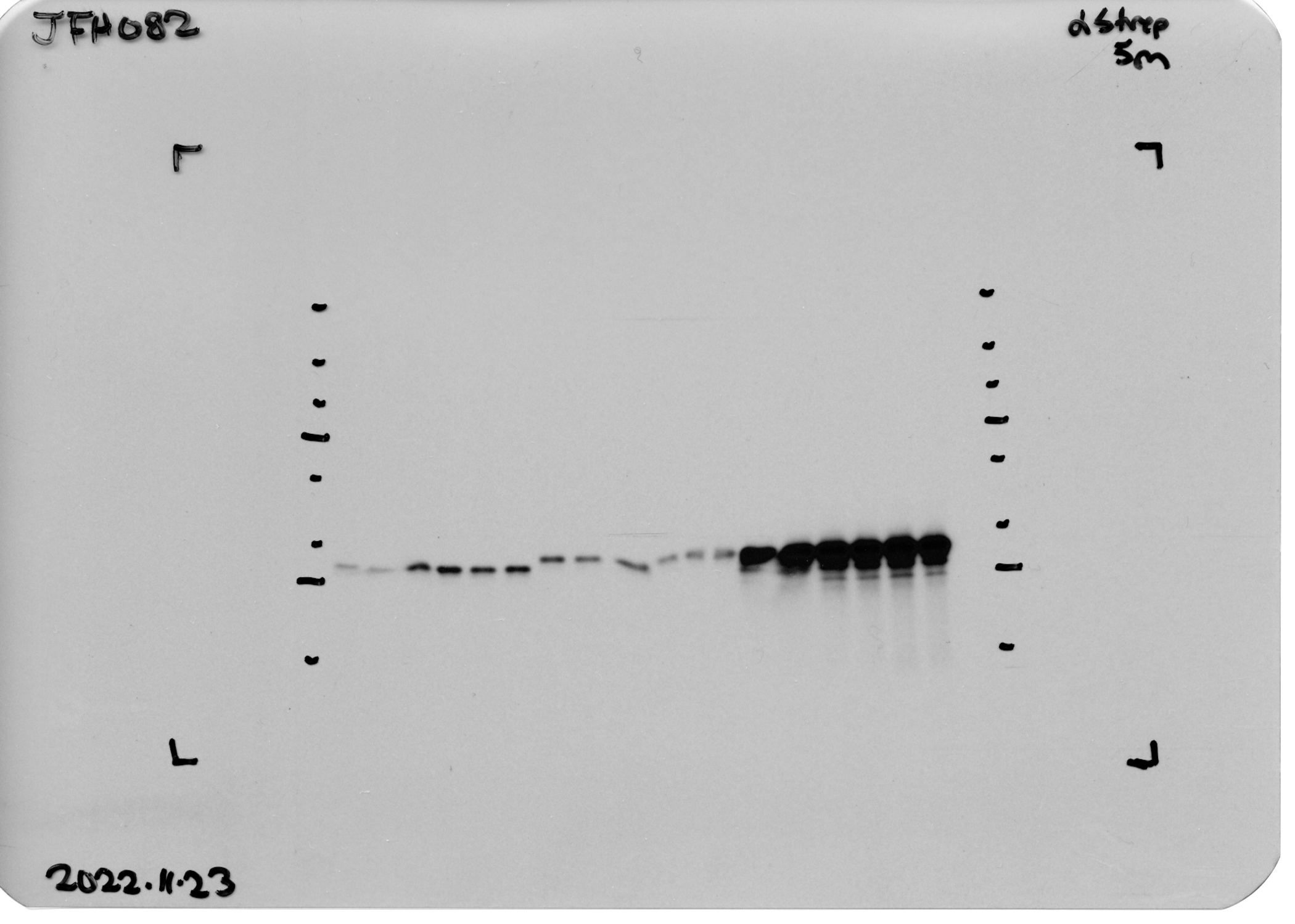

Supplement: Supplementary file 11 — Source Data [file 41467_2023_41442_MOESM11_ESM.zip › Haas_SourceData/Western Blot Scans (Supp Fig 3)/NHBE/JFH082 - Strep - 5m.tif]

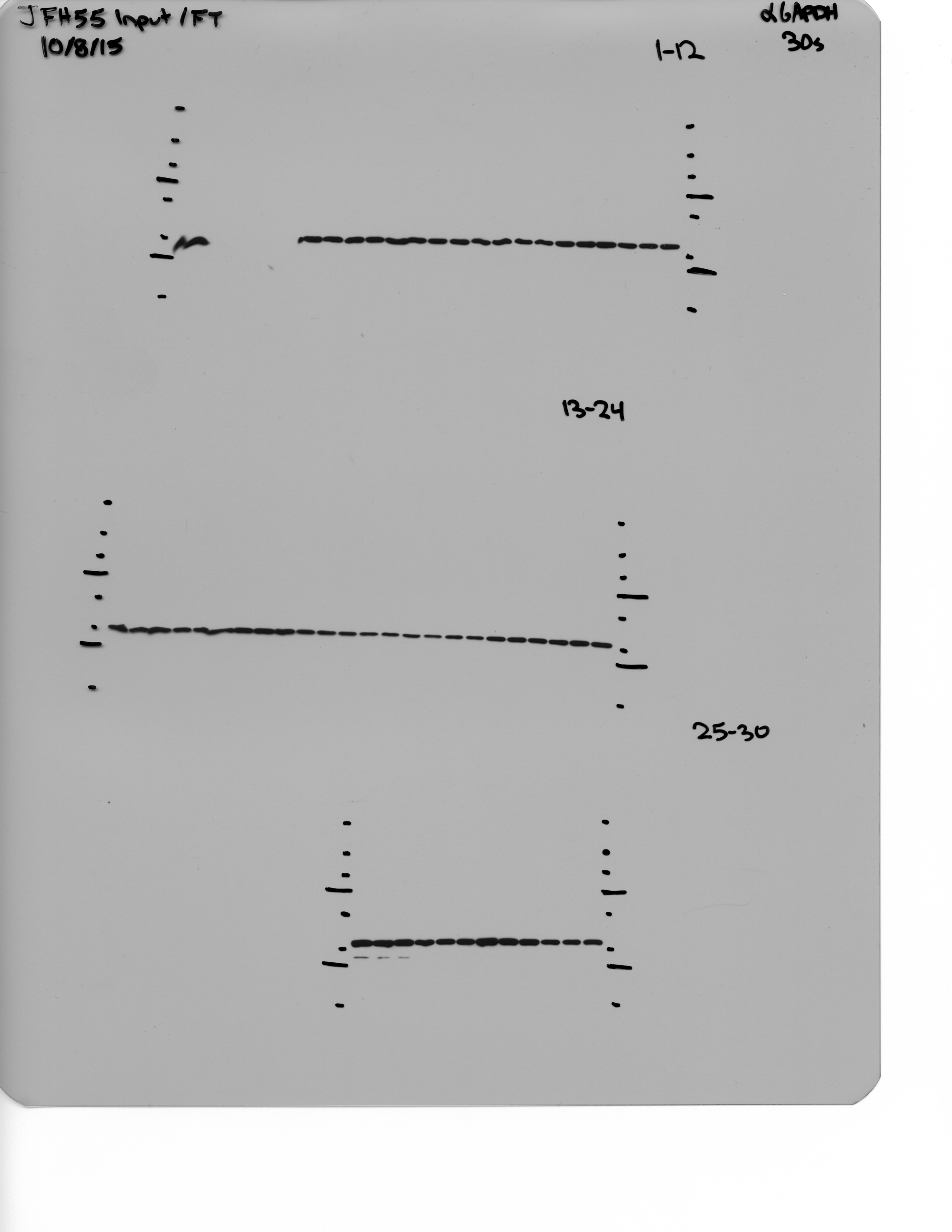

Supplement: Supplementary file 11 — Source Data [file 41467_2023_41442_MOESM11_ESM.zip › Haas_SourceData/Western Blot Scans (Supp Fig 3)/NHBE/JFH055 - GAPDH - 30s.tif]

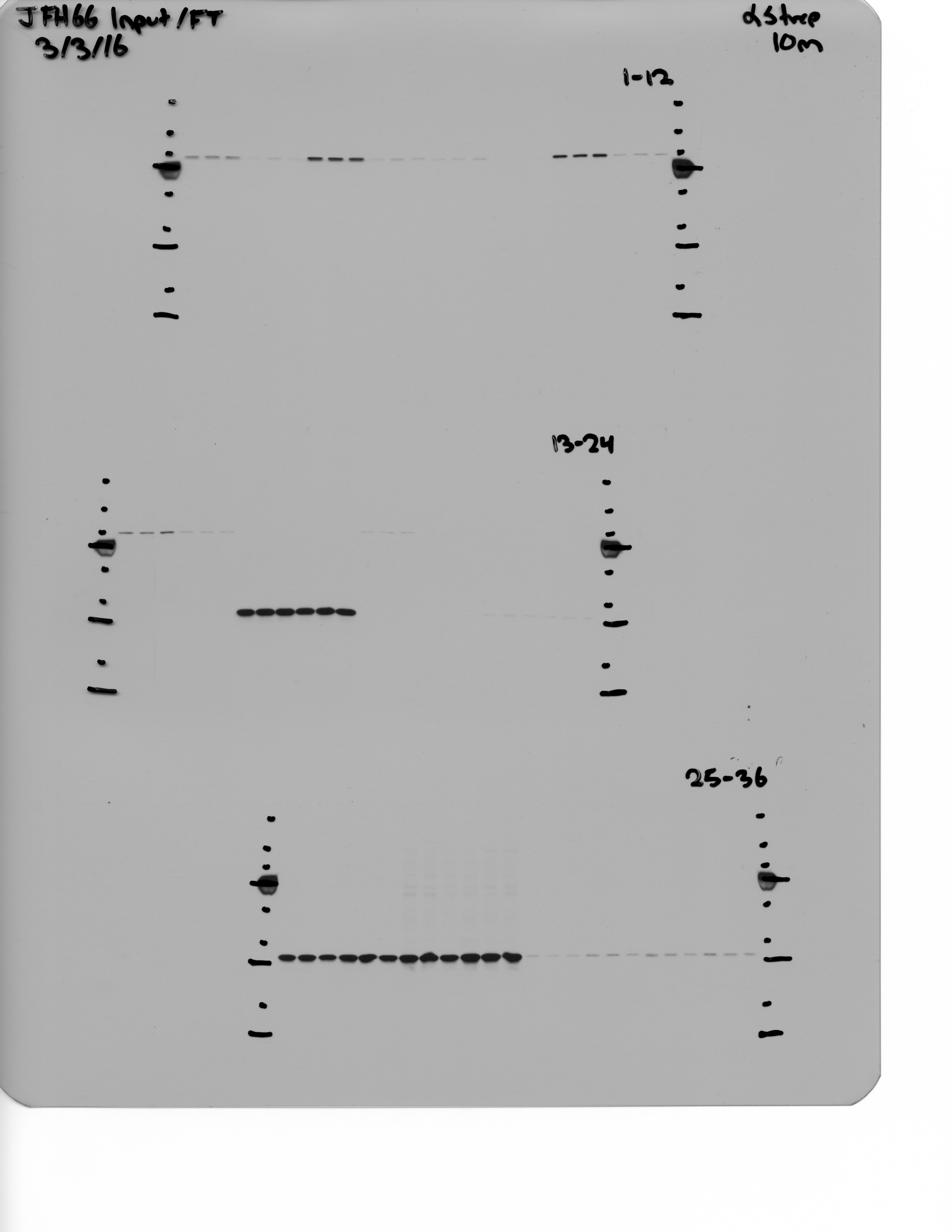

Supplement: Supplementary file 11 — Source Data [file 41467_2023_41442_MOESM11_ESM.zip › Haas_SourceData/Western Blot Scans (Supp Fig 3)/NHBE/JFH066 - Strep - 10m.tif]

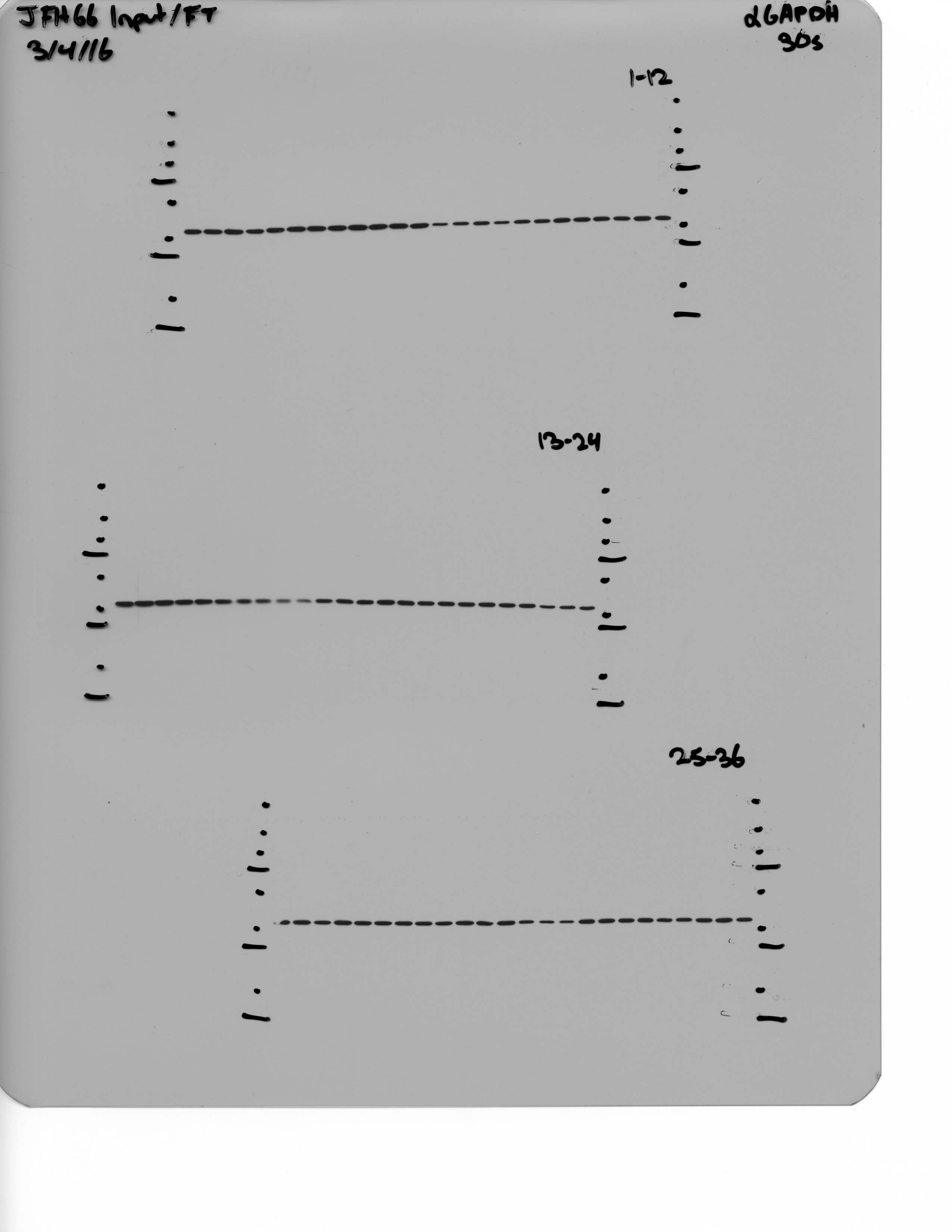

Supplement: Supplementary file 11 — Source Data [file 41467_2023_41442_MOESM11_ESM.zip › Haas_SourceData/Western Blot Scans (Supp Fig 3)/NHBE/JFH066 - GAPDH - 30s.tif]

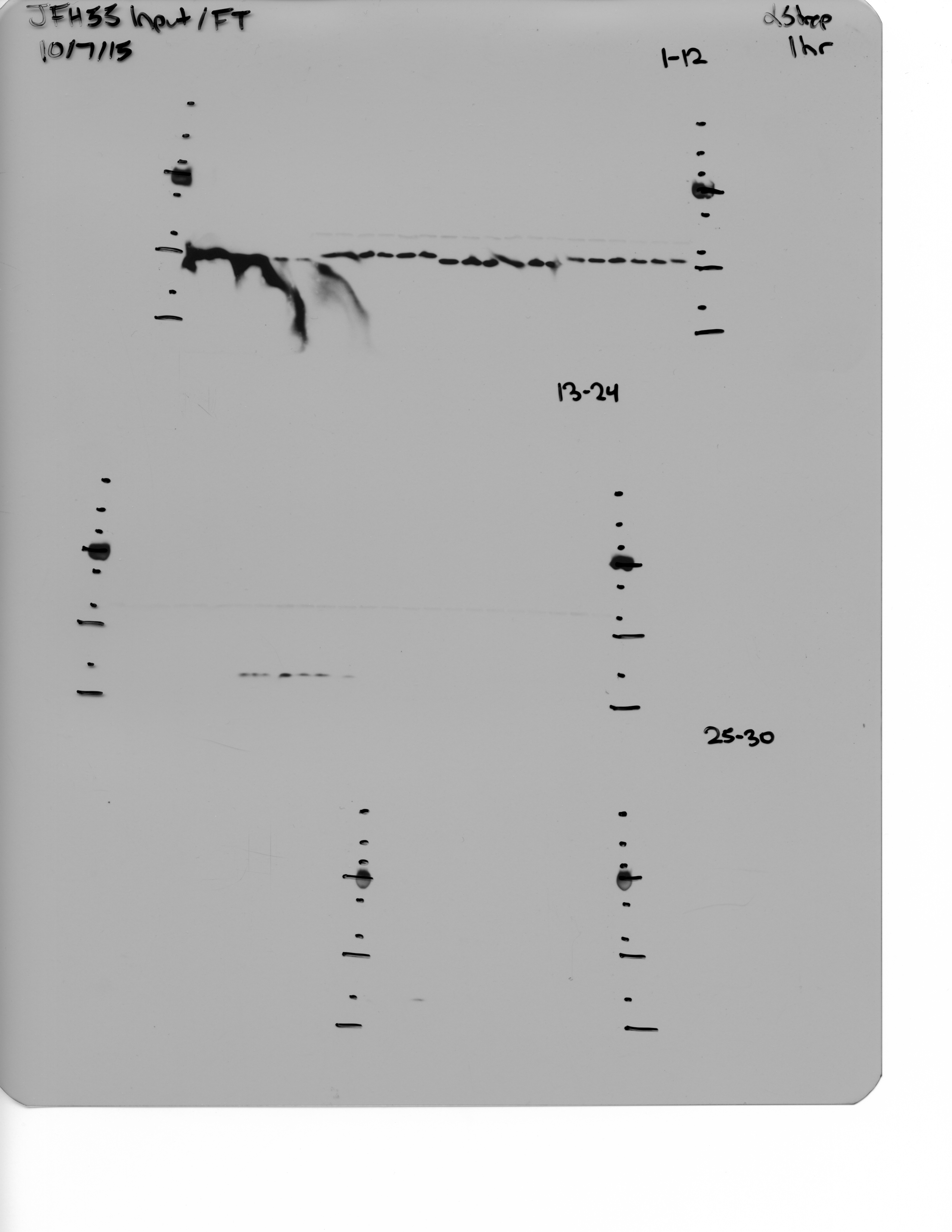

Supplement: Supplementary file 11 — Source Data [file 41467_2023_41442_MOESM11_ESM.zip › Haas_SourceData/Western Blot Scans (Supp Fig 3)/NHBE/JFH055 - Strep - 60m.tif]

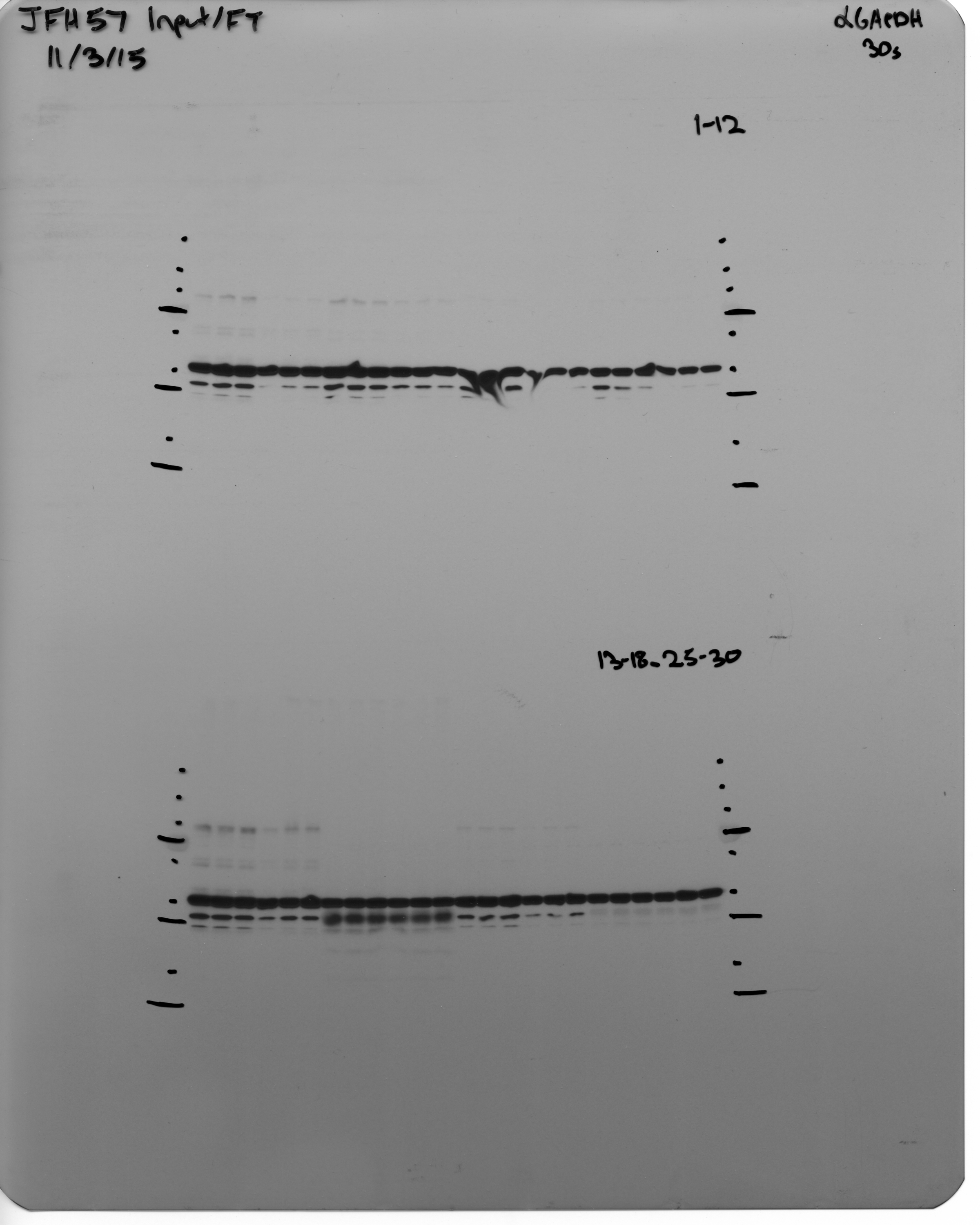

Supplement: Supplementary file 11 — Source Data [file 41467_2023_41442_MOESM11_ESM.zip › Haas_SourceData/Western Blot Scans (Supp Fig 3)/NHBE/JFH057 - GAPDH - 30s.tif]

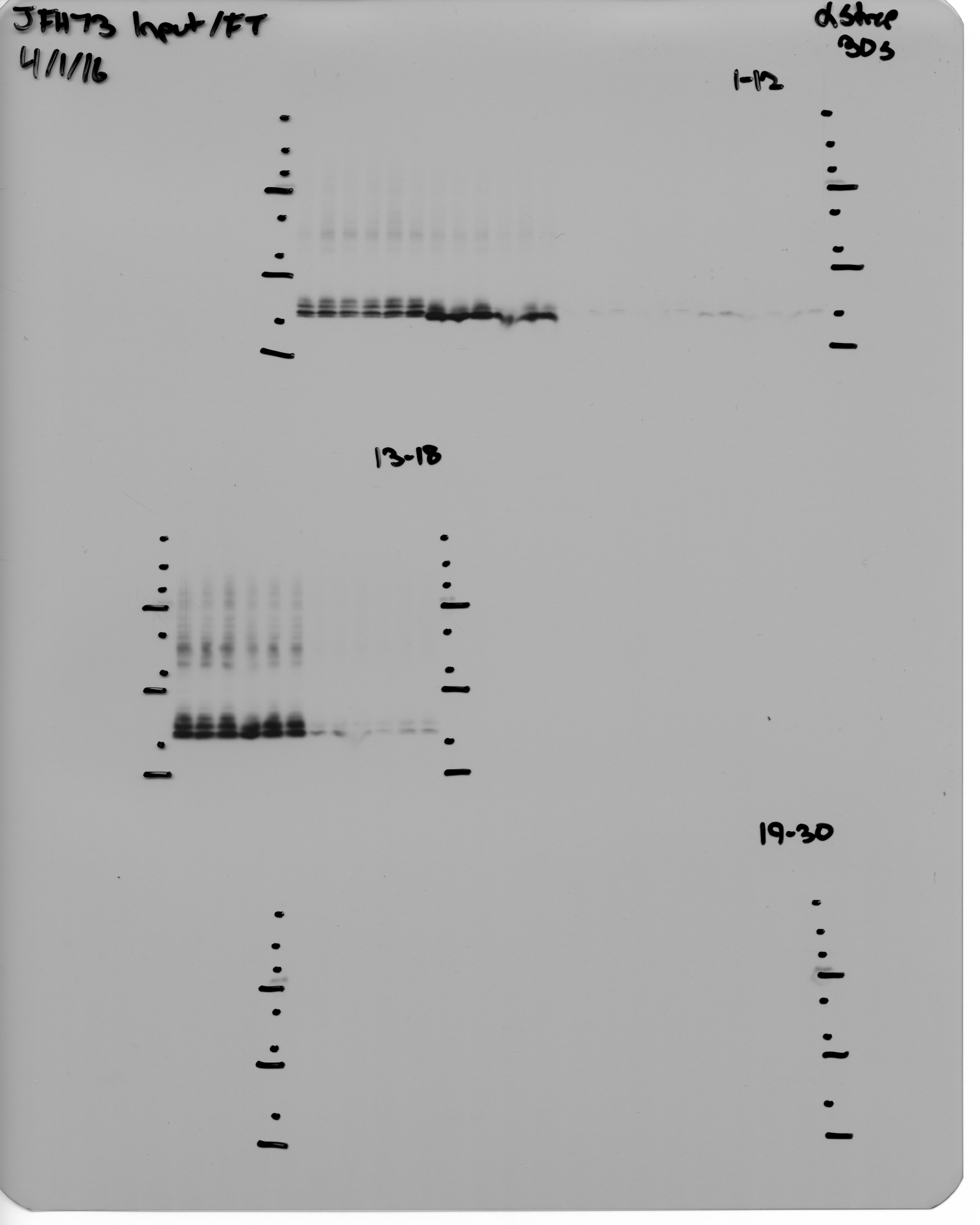

Supplement: Supplementary file 11 — Source Data [file 41467_2023_41442_MOESM11_ESM.zip › Haas_SourceData/Western Blot Scans (Supp Fig 3)/NHBE/JFH073 - Strep - 30s.tif]

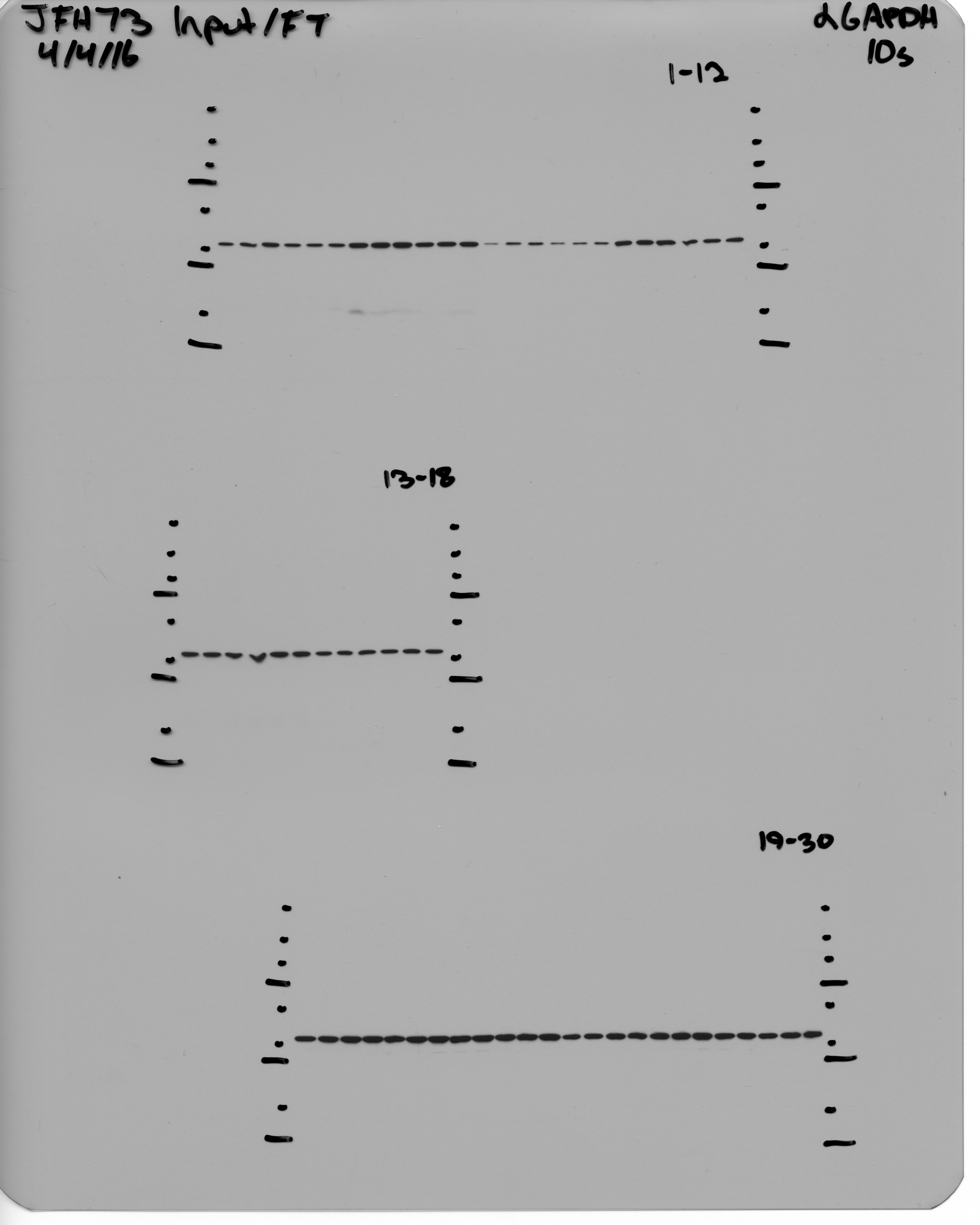

Supplement: Supplementary file 11 — Source Data [file 41467_2023_41442_MOESM11_ESM.zip › Haas_SourceData/Western Blot Scans (Supp Fig 3)/NHBE/JFH073 - GAPDH - 10s.tif]

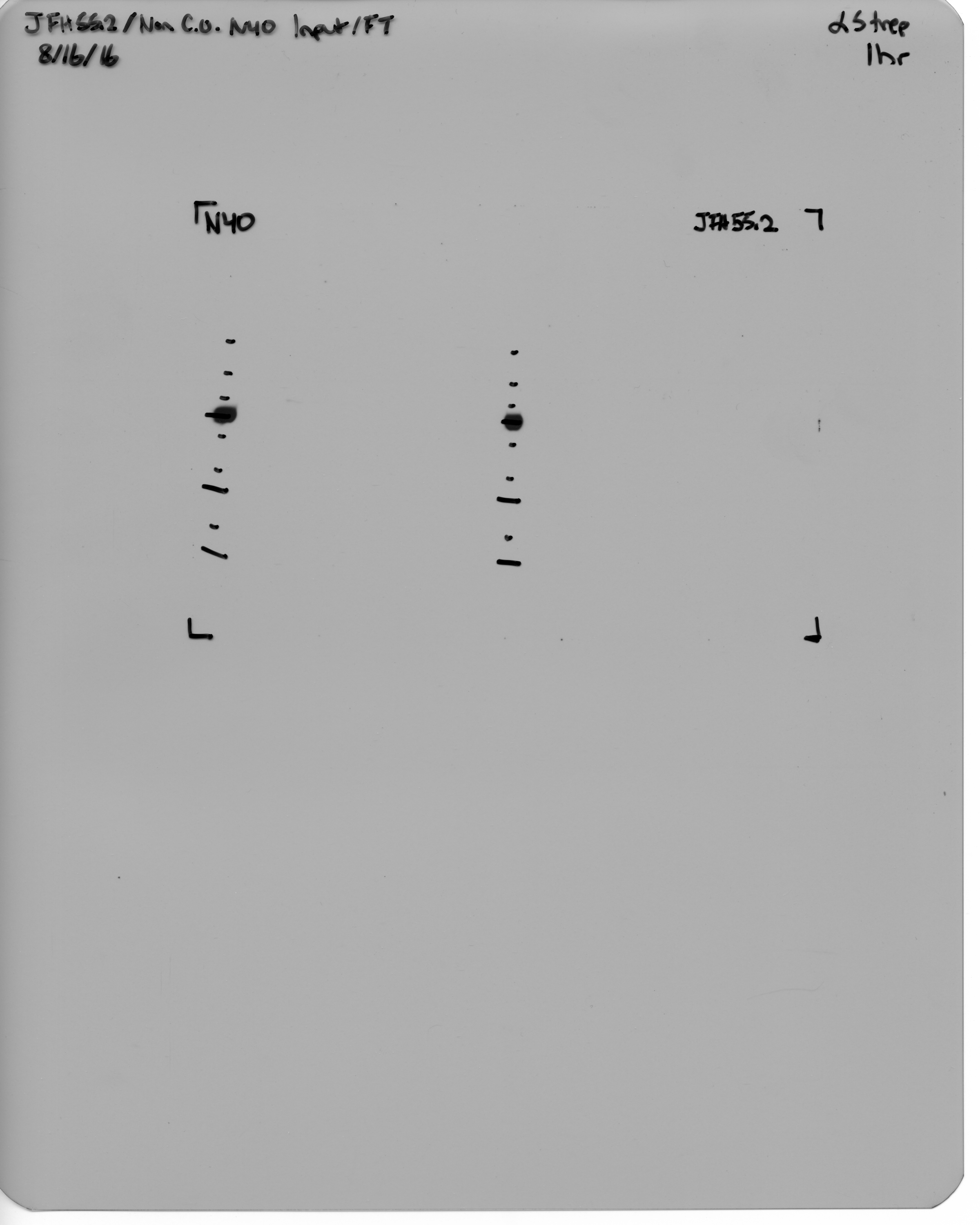

Supplement: Supplementary file 11 — Source Data [file 41467_2023_41442_MOESM11_ESM.zip › Haas_SourceData/Western Blot Scans (Supp Fig 3)/NHBE/JFH055.2 - Strep - 60m.tif]

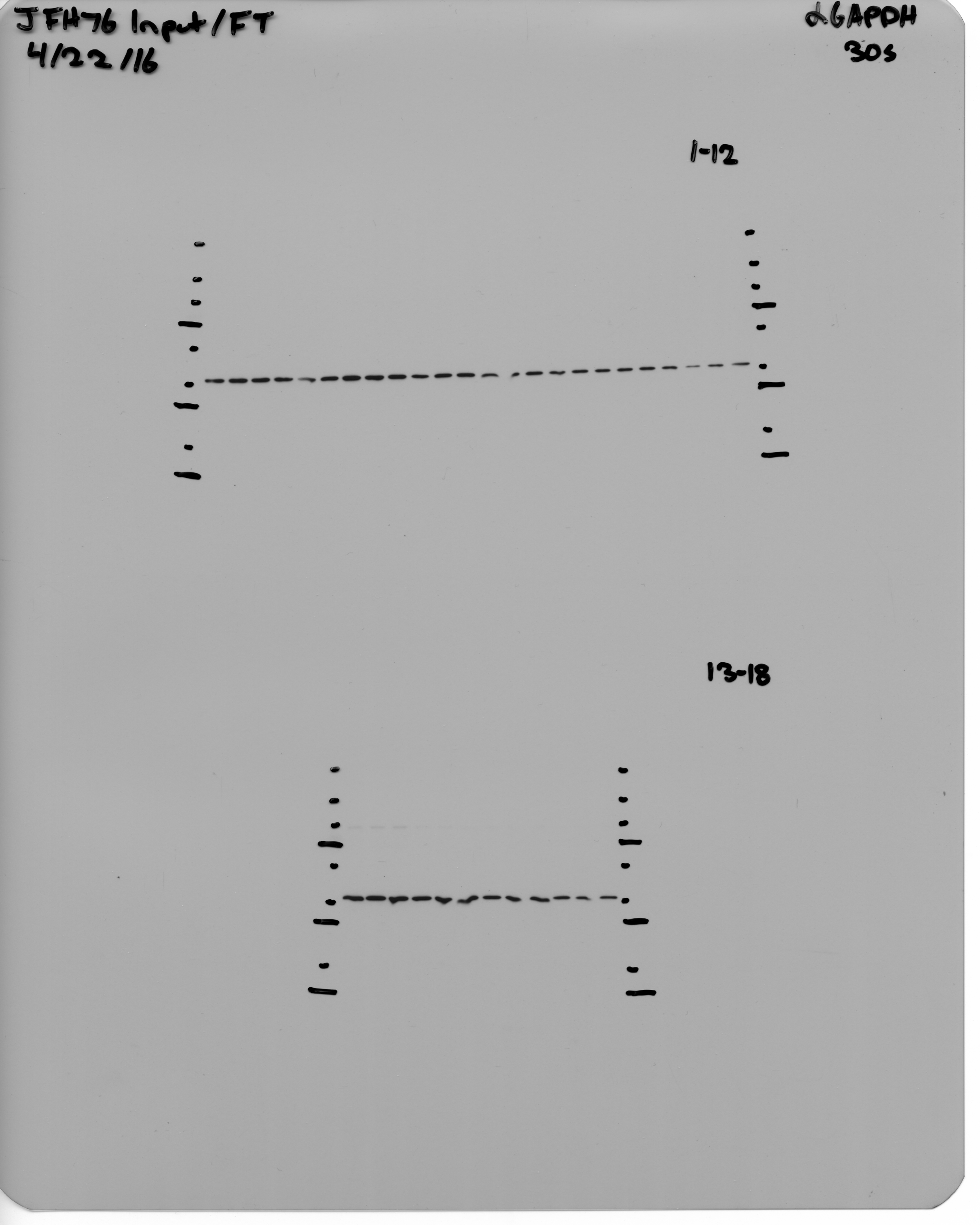

Supplement: Supplementary file 11 — Source Data [file 41467_2023_41442_MOESM11_ESM.zip › Haas_SourceData/Western Blot Scans (Supp Fig 3)/NHBE/JFH076 - GAPDH - 30s.tif]

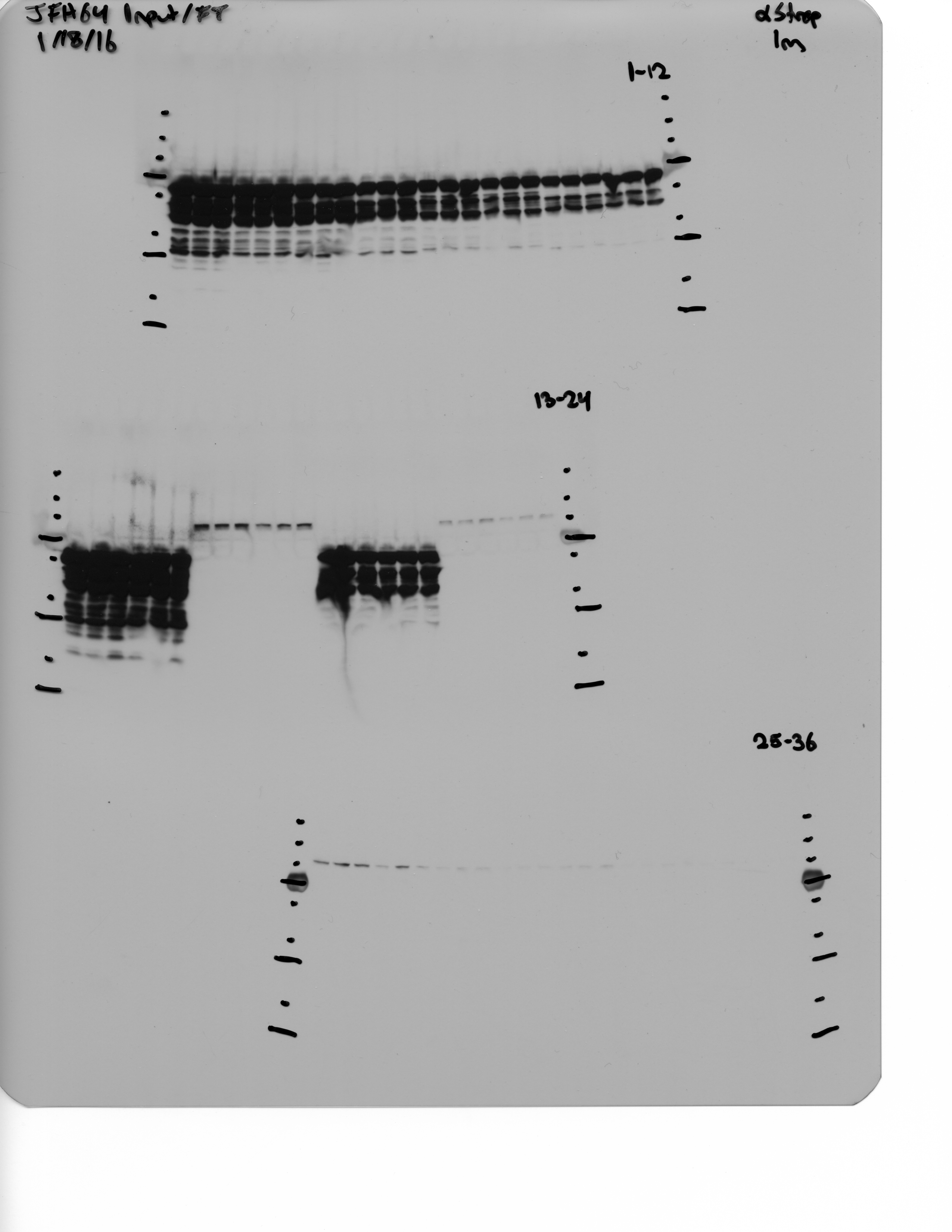

Supplement: Supplementary file 11 — Source Data [file 41467_2023_41442_MOESM11_ESM.zip › Haas_SourceData/Western Blot Scans (Supp Fig 3)/NHBE/JFH064 - Strep - 1m.tif]

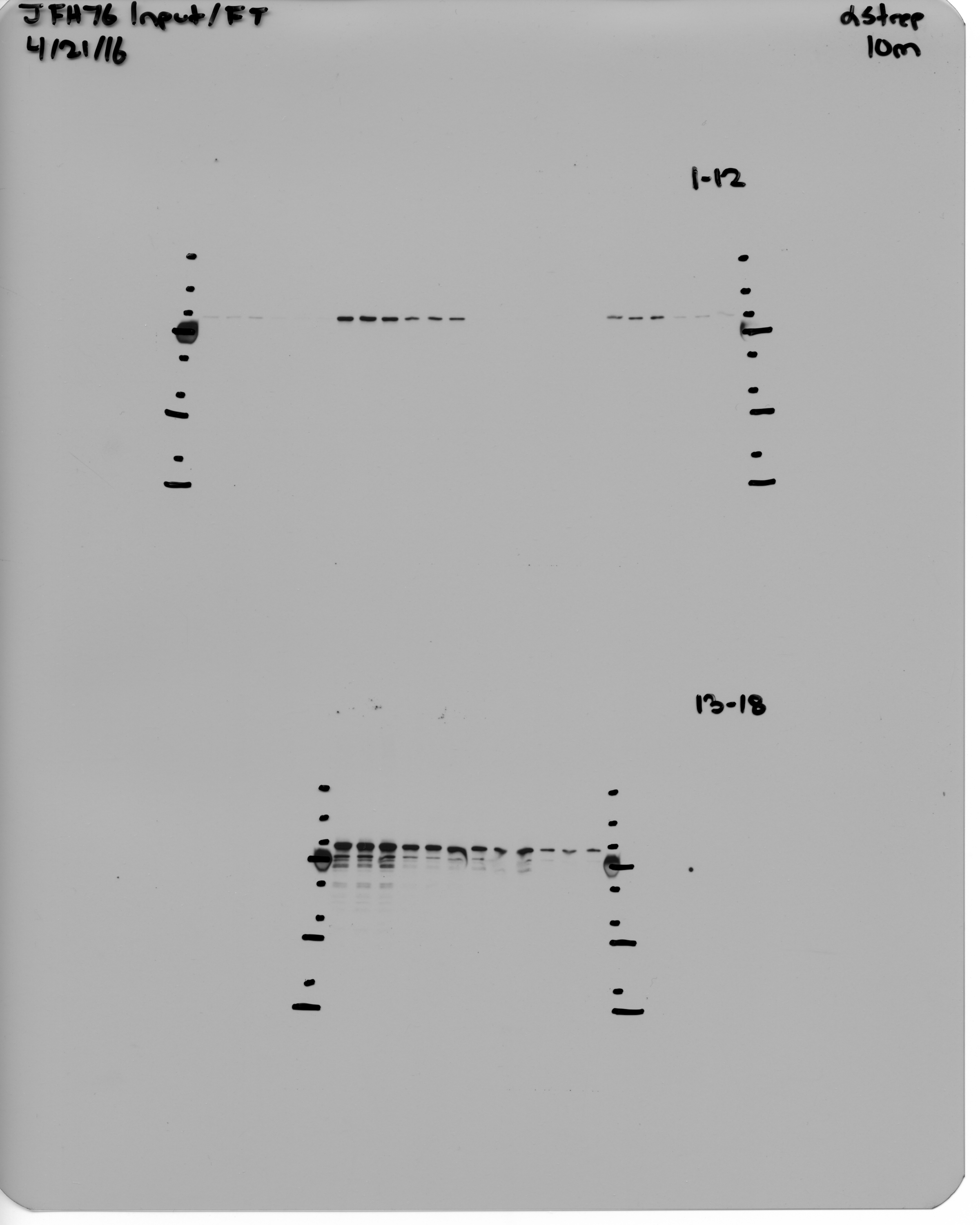

Supplement: Supplementary file 11 — Source Data [file 41467_2023_41442_MOESM11_ESM.zip › Haas_SourceData/Western Blot Scans (Supp Fig 3)/NHBE/JFH076 - Strep - 10m.tif]

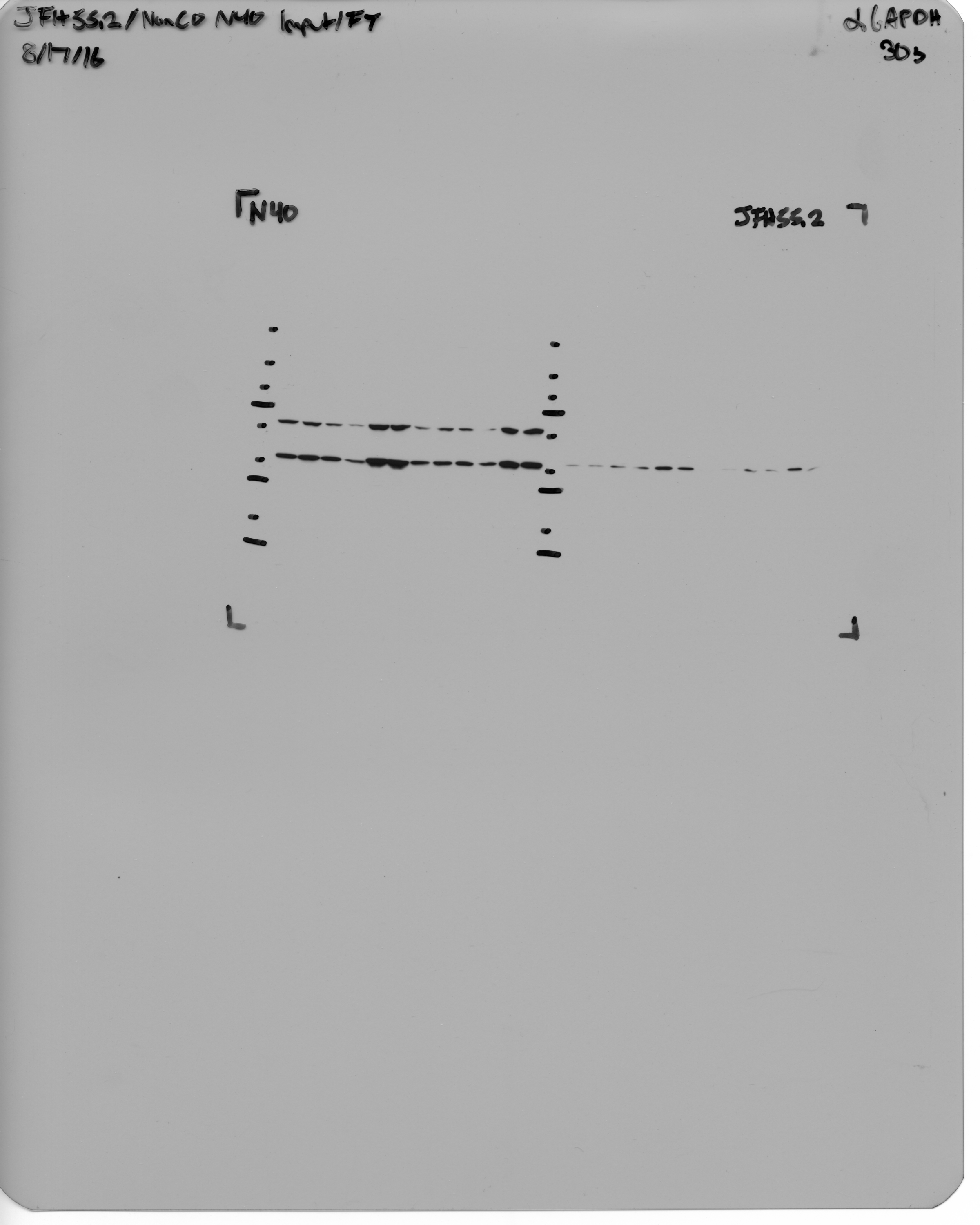

Supplement: Supplementary file 11 — Source Data [file 41467_2023_41442_MOESM11_ESM.zip › Haas_SourceData/Western Blot Scans (Supp Fig 3)/NHBE/JFH055.2 - GAPDH - 30s.tif]

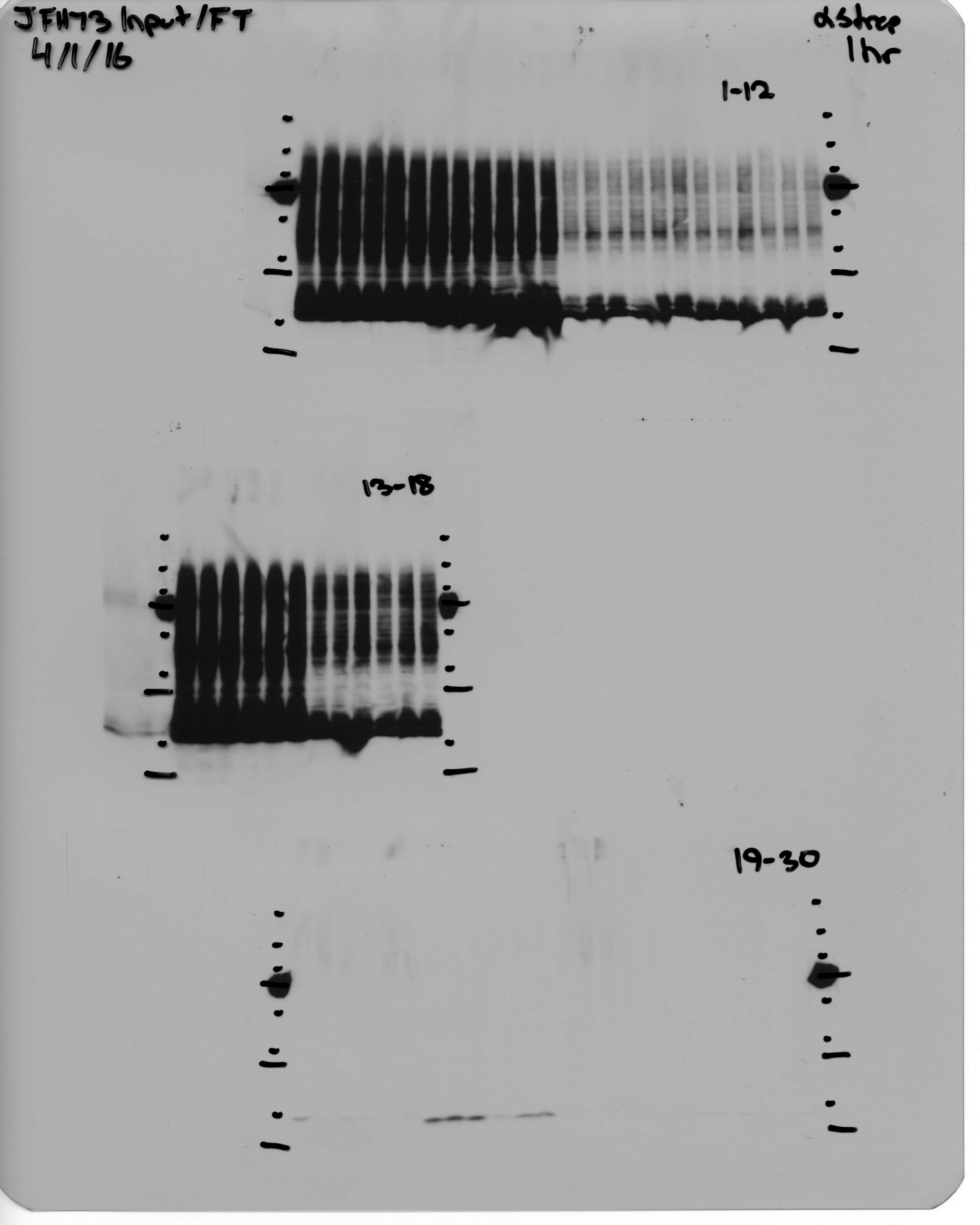

Supplement: Supplementary file 11 — Source Data [file 41467_2023_41442_MOESM11_ESM.zip › Haas_SourceData/Western Blot Scans (Supp Fig 3)/NHBE/JFH073 - Strep - 60m.tif]

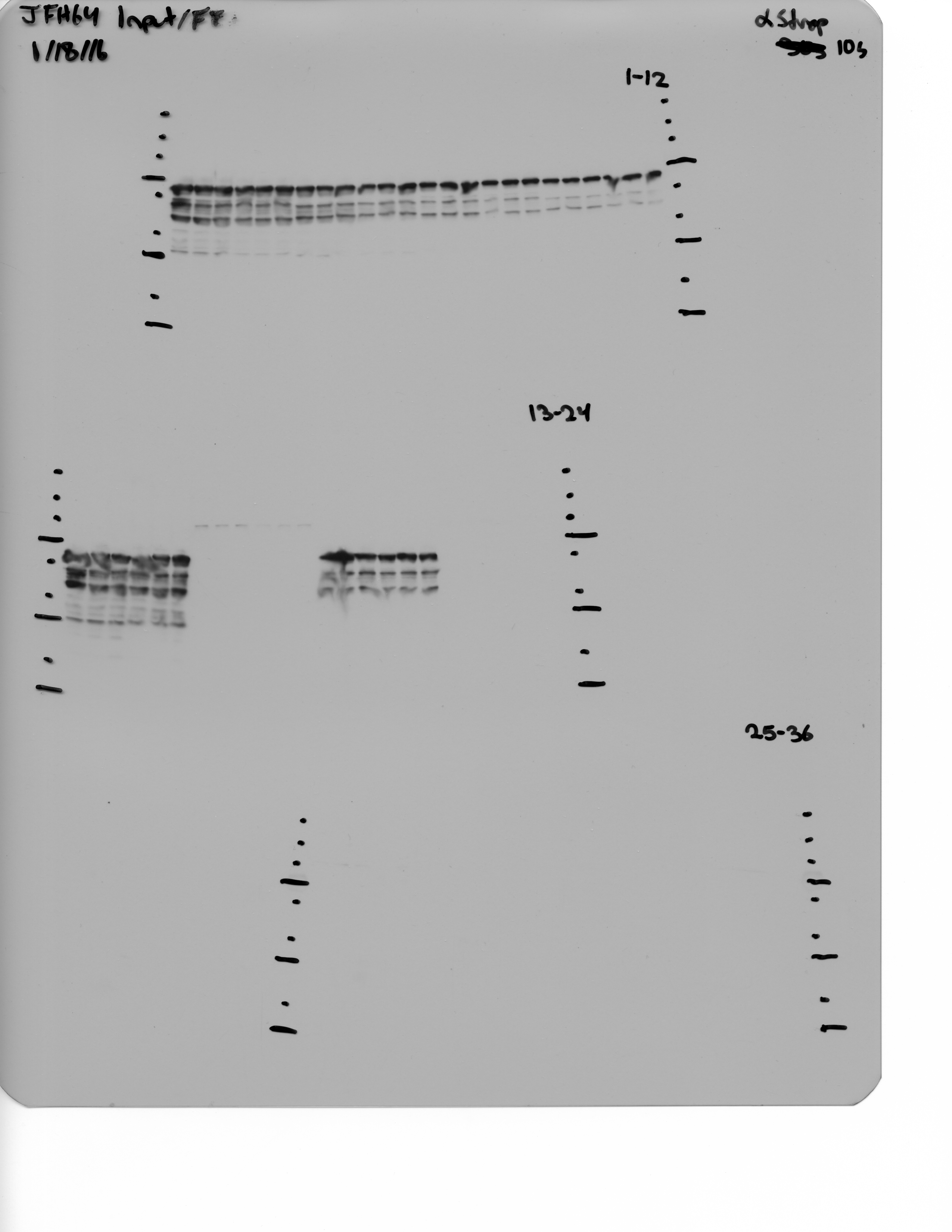

Supplement: Supplementary file 11 — Source Data [file 41467_2023_41442_MOESM11_ESM.zip › Haas_SourceData/Western Blot Scans (Supp Fig 3)/NHBE/JFH064 - Strep - 10s.tif]

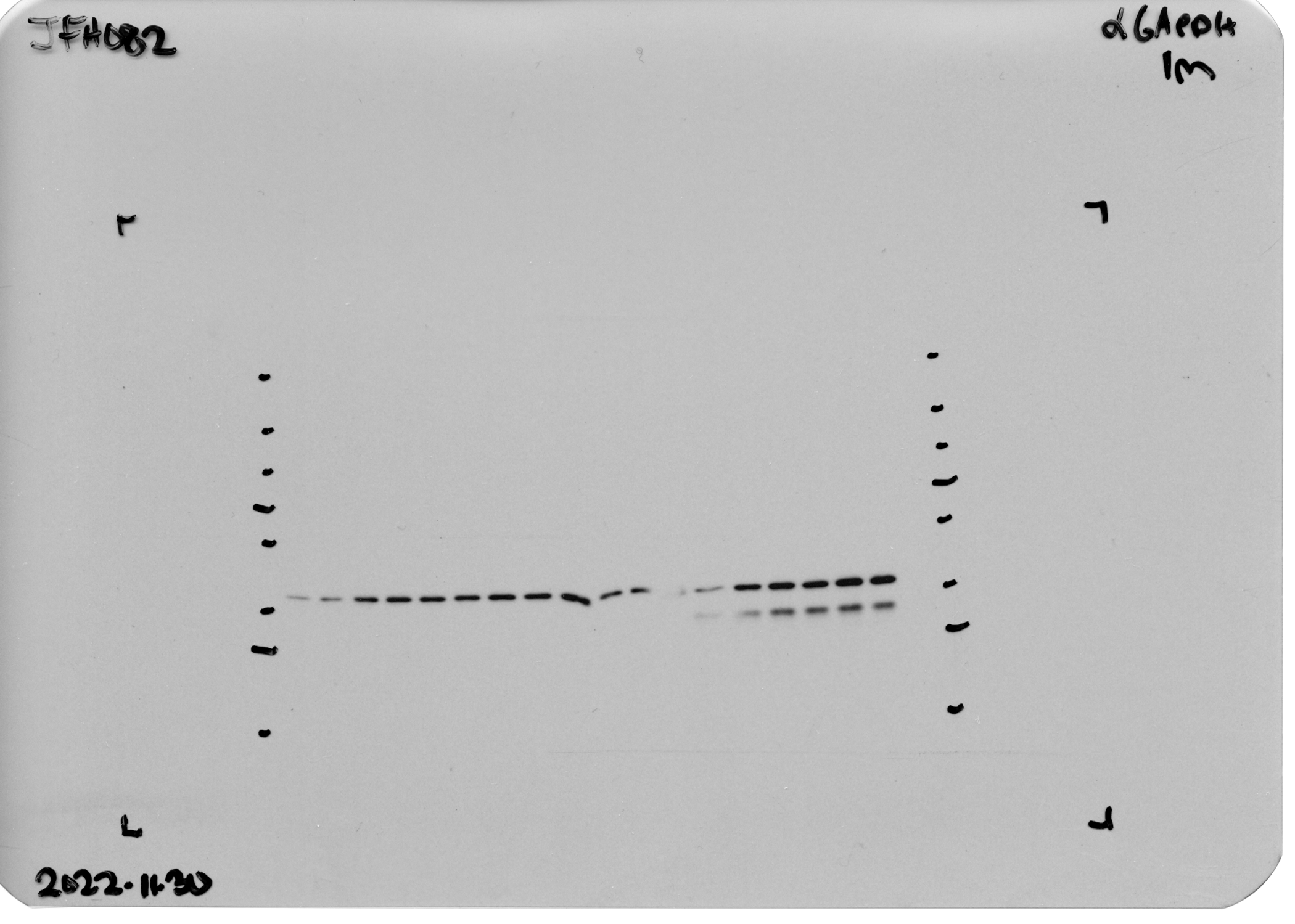

Supplement: Supplementary file 11 — Source Data [file 41467_2023_41442_MOESM11_ESM.zip › Haas_SourceData/Western Blot Scans (Supp Fig 3)/NHBE/JFH082 - GAPDH - 1m.tif]

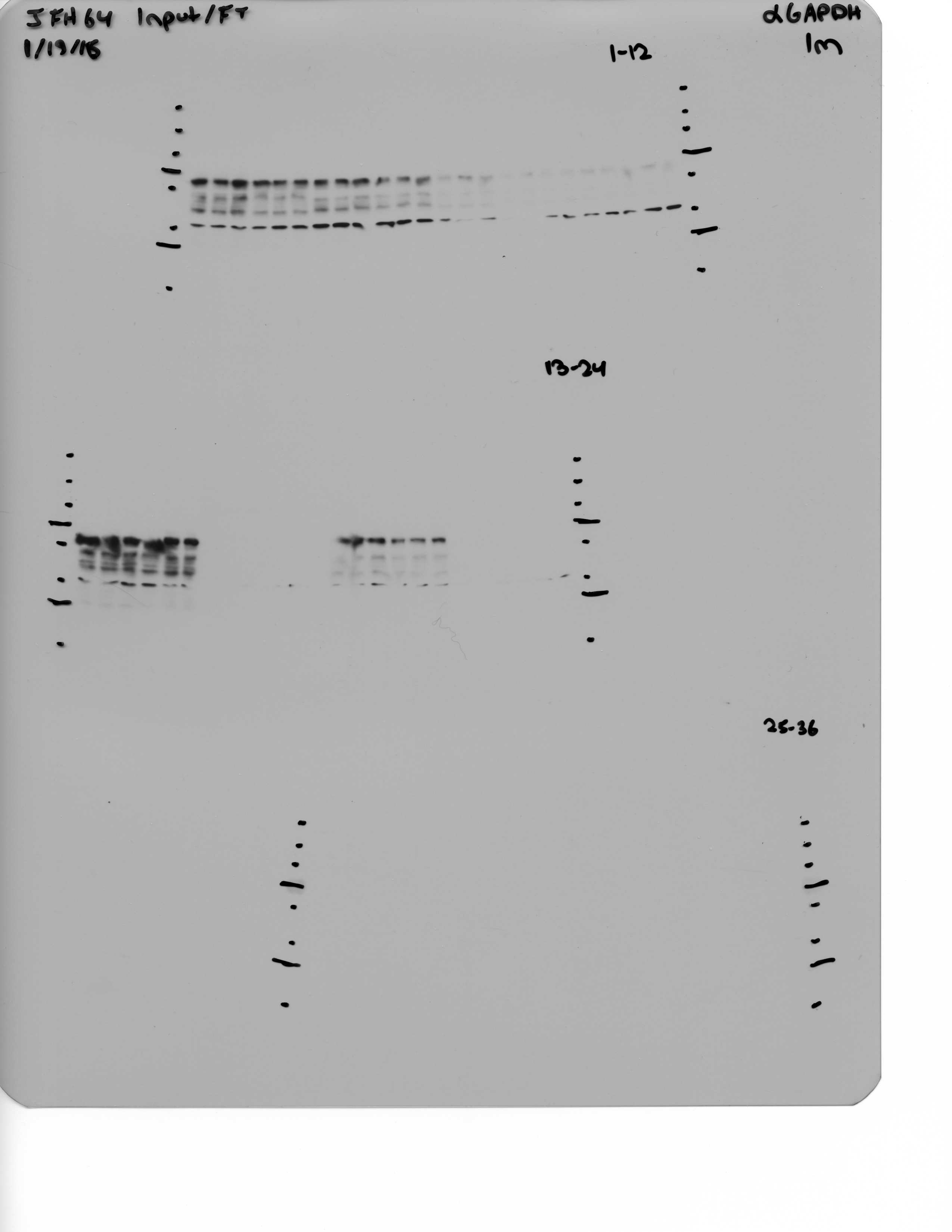

Supplement: Supplementary file 11 — Source Data [file 41467_2023_41442_MOESM11_ESM.zip › Haas_SourceData/Western Blot Scans (Supp Fig 3)/NHBE/JFH064 - GAPDH - 1m.tif]

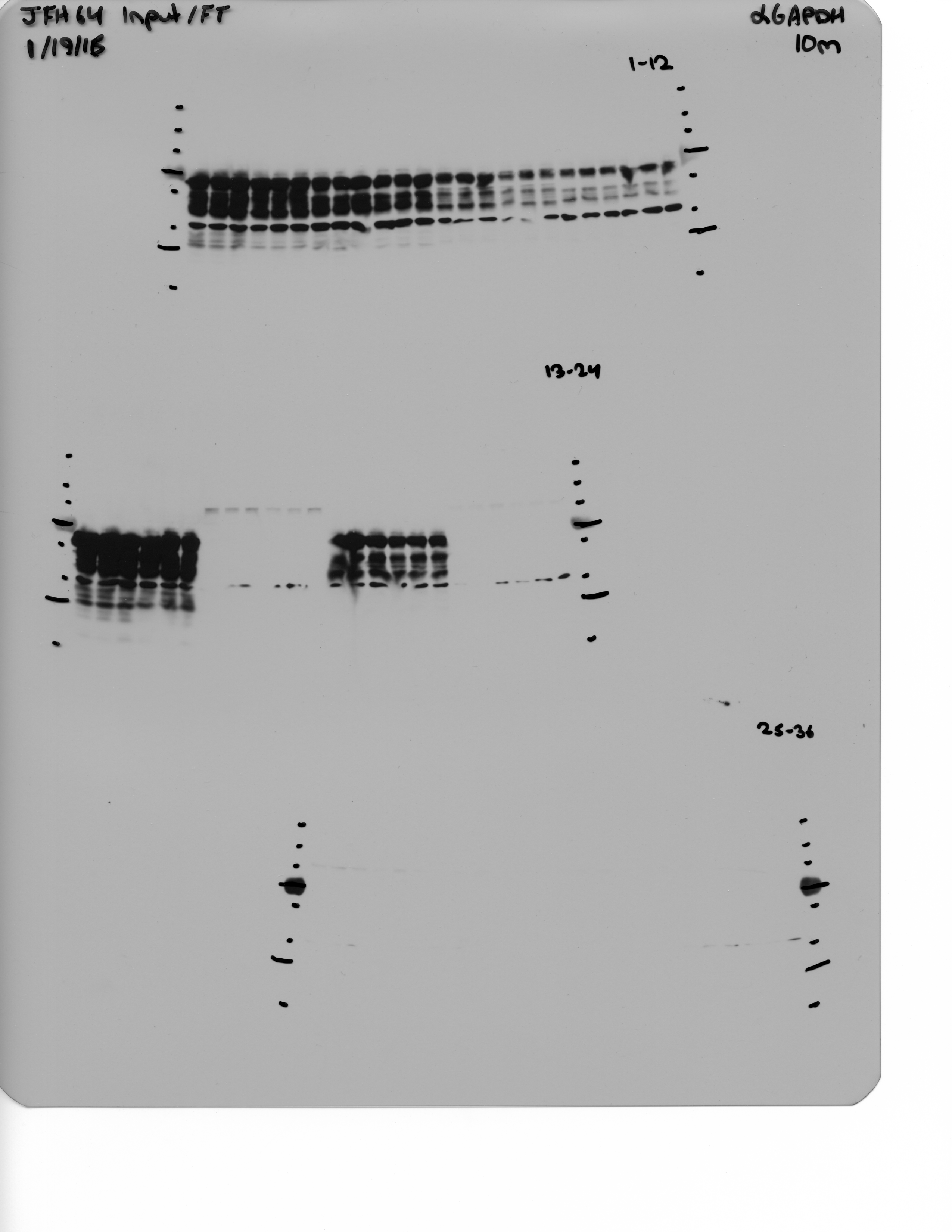

Supplement: Supplementary file 11 — Source Data [file 41467_2023_41442_MOESM11_ESM.zip › Haas_SourceData/Western Blot Scans (Supp Fig 3)/NHBE/JFH064 - GAPDH - 10m.tif]

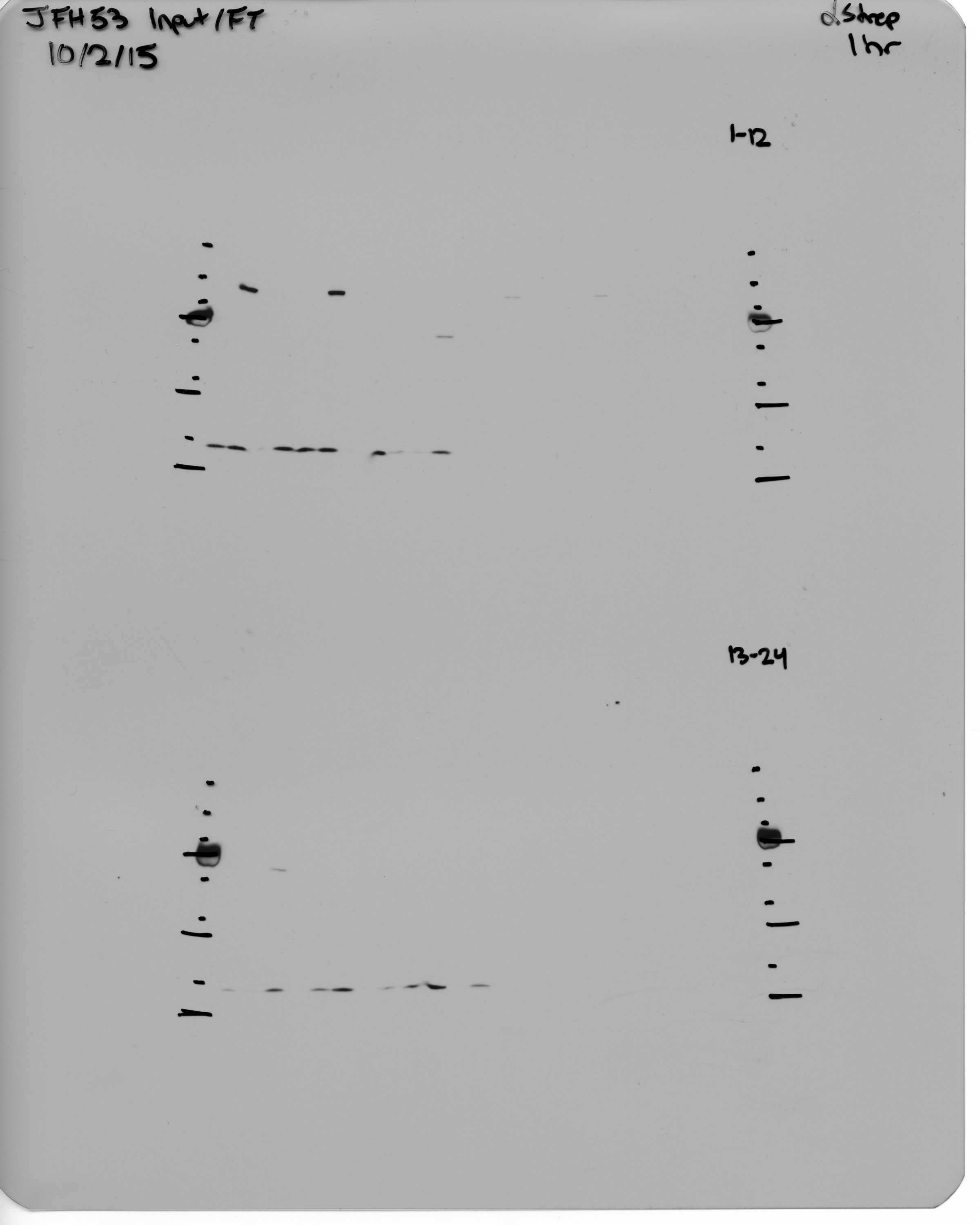

Supplement: Supplementary file 11 — Source Data [file 41467_2023_41442_MOESM11_ESM.zip › Haas_SourceData/Western Blot Scans (Supp Fig 3)/THP-1/JFH053 - Strep - 60m.tif]

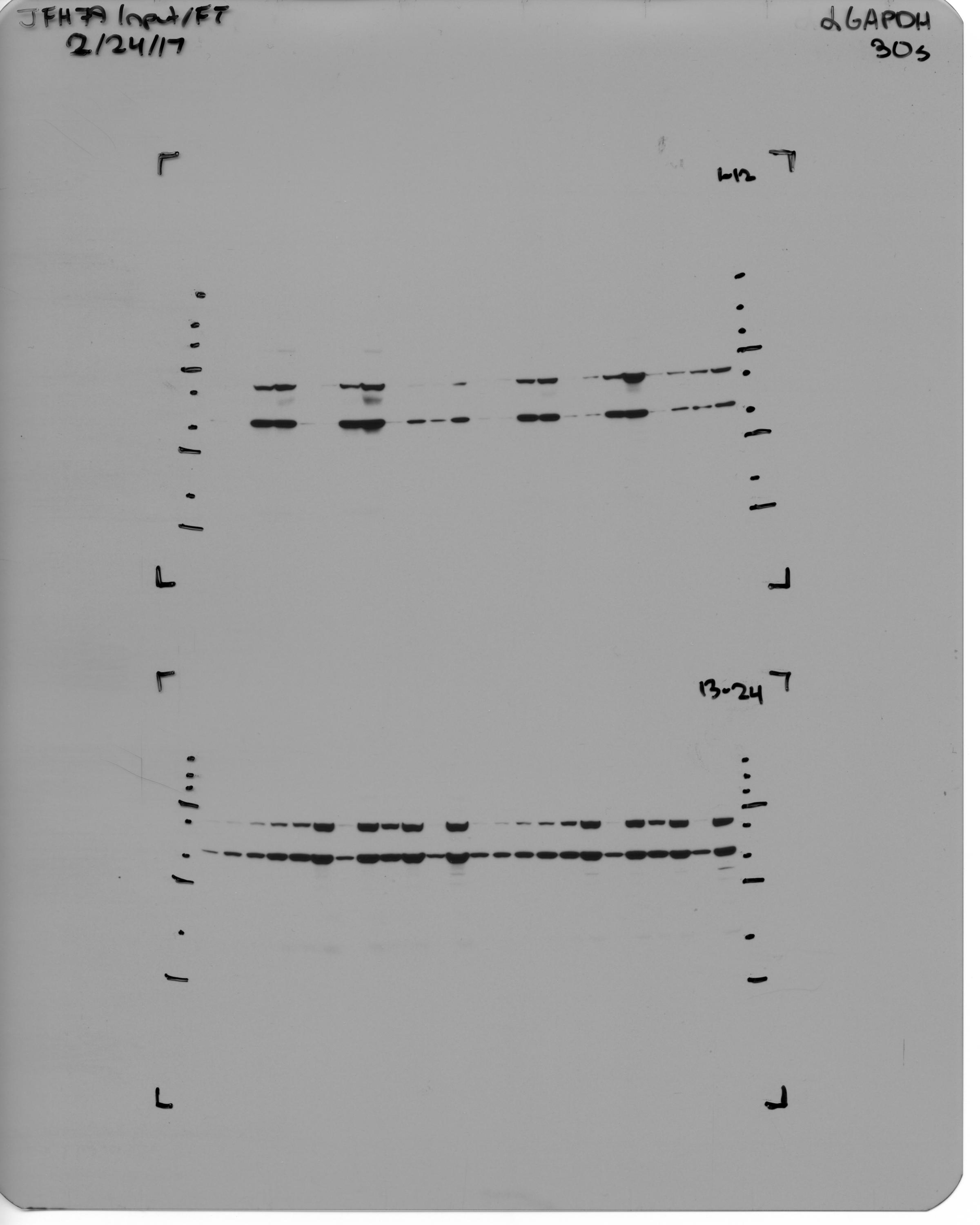

Supplement: Supplementary file 11 — Source Data [file 41467_2023_41442_MOESM11_ESM.zip › Haas_SourceData/Western Blot Scans (Supp Fig 3)/THP-1/JFH079 - GAPDH - 30s.tif]

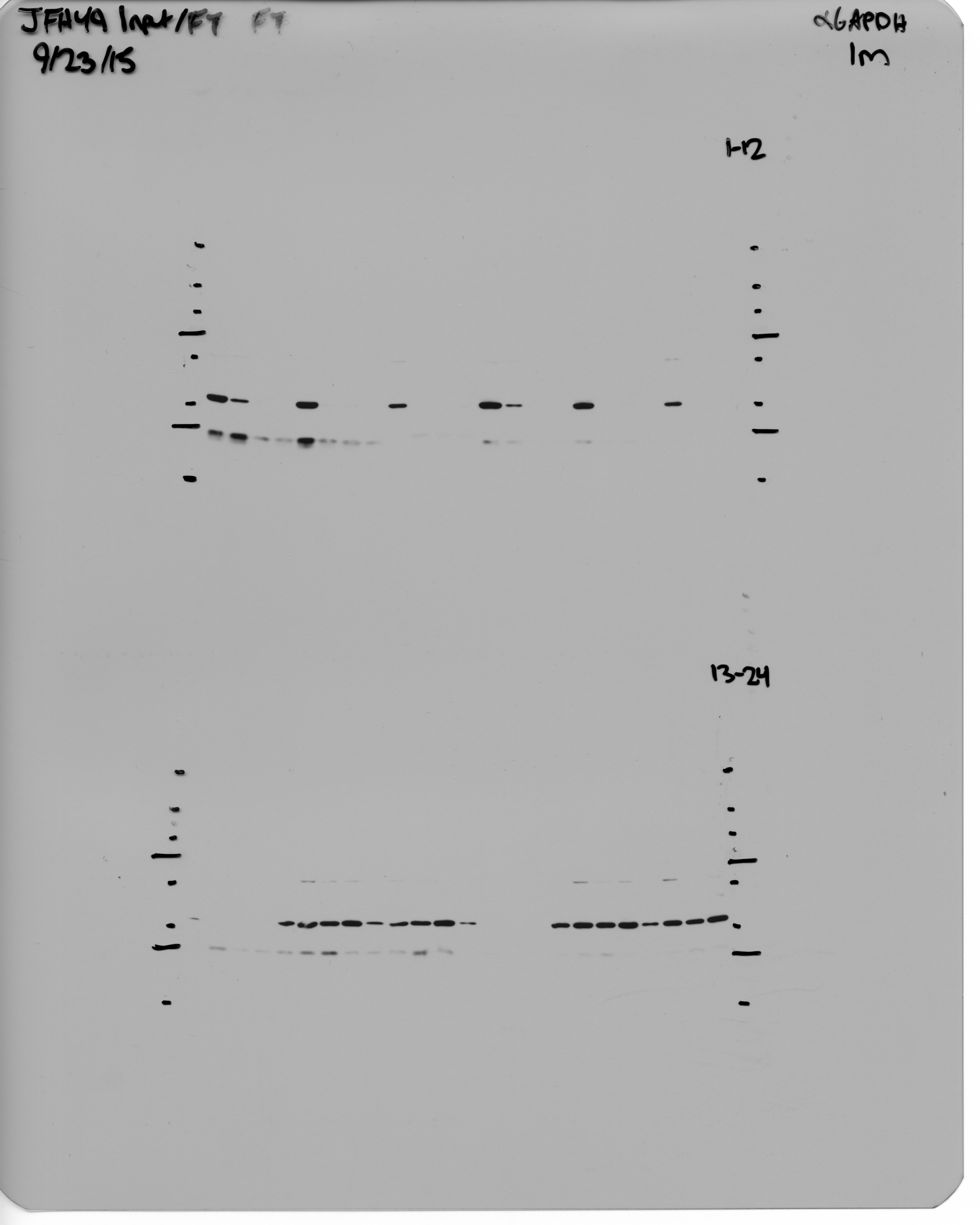

Supplement: Supplementary file 11 — Source Data [file 41467_2023_41442_MOESM11_ESM.zip › Haas_SourceData/Western Blot Scans (Supp Fig 3)/THP-1/JFH049 - GAPDH - 1m.tif]

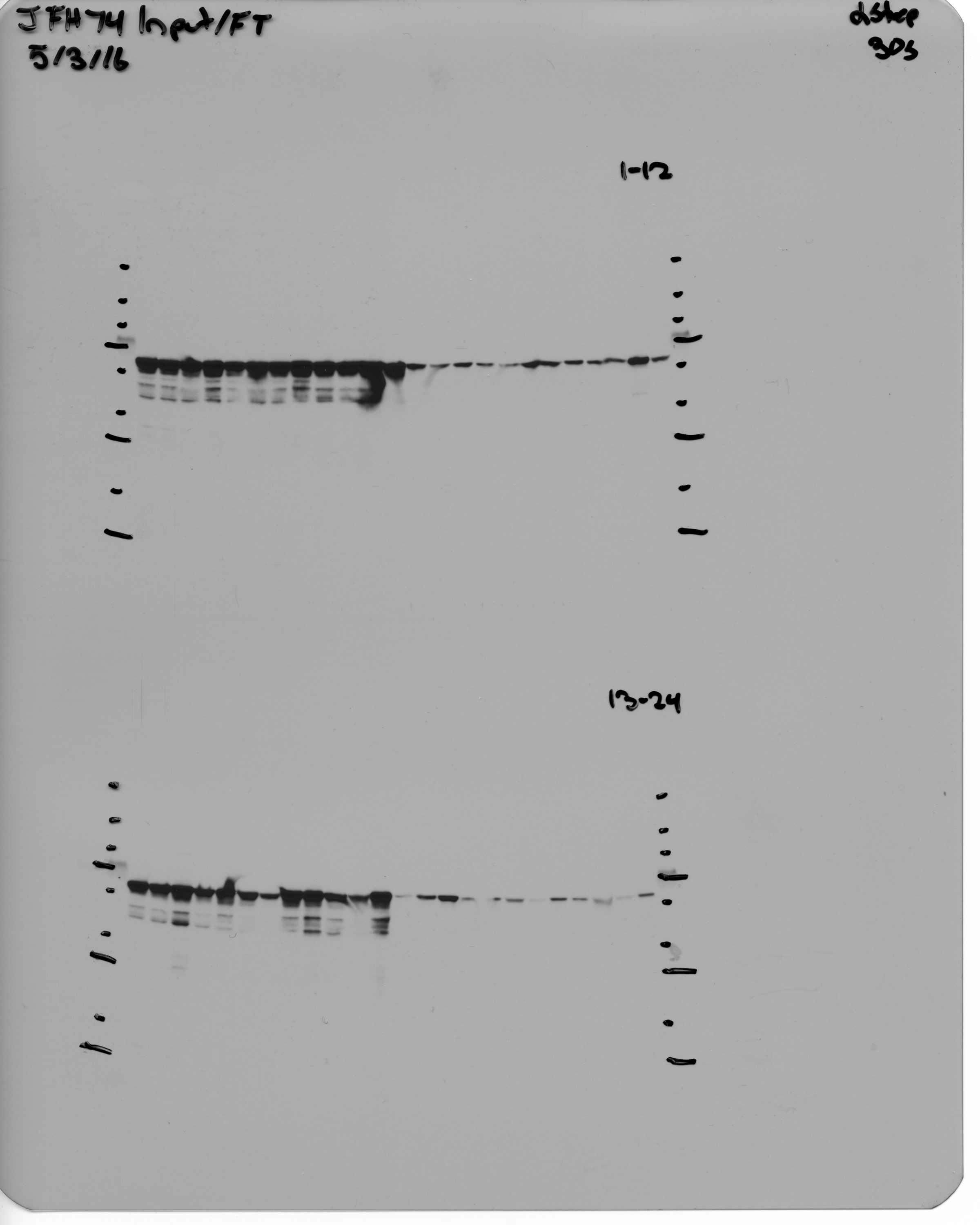

Supplement: Supplementary file 11 — Source Data [file 41467_2023_41442_MOESM11_ESM.zip › Haas_SourceData/Western Blot Scans (Supp Fig 3)/THP-1/JFH074 - Strep - 30s.tif]

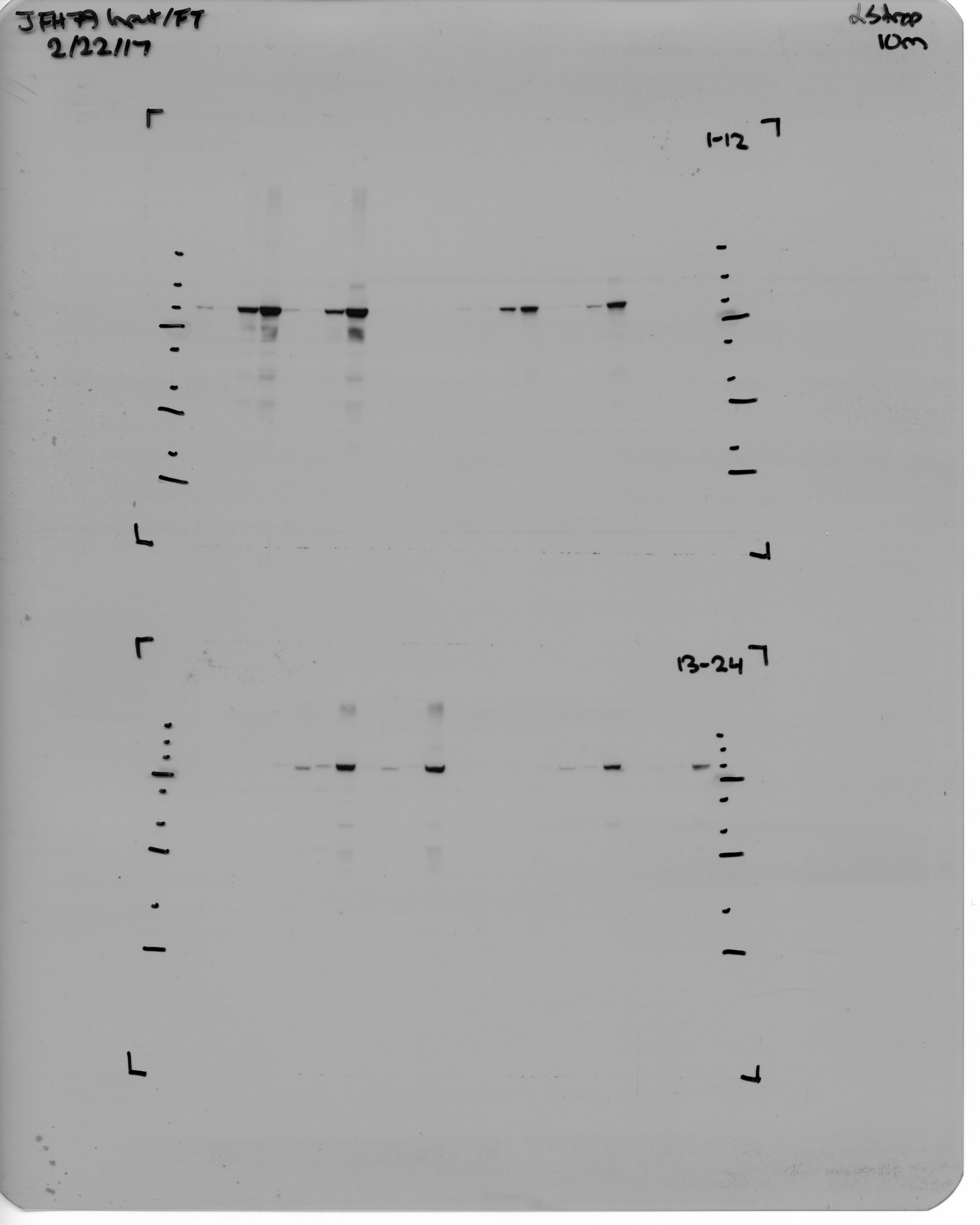

Supplement: Supplementary file 11 — Source Data [file 41467_2023_41442_MOESM11_ESM.zip › Haas_SourceData/Western Blot Scans (Supp Fig 3)/THP-1/JFH079 - Strep - 10m.tif]

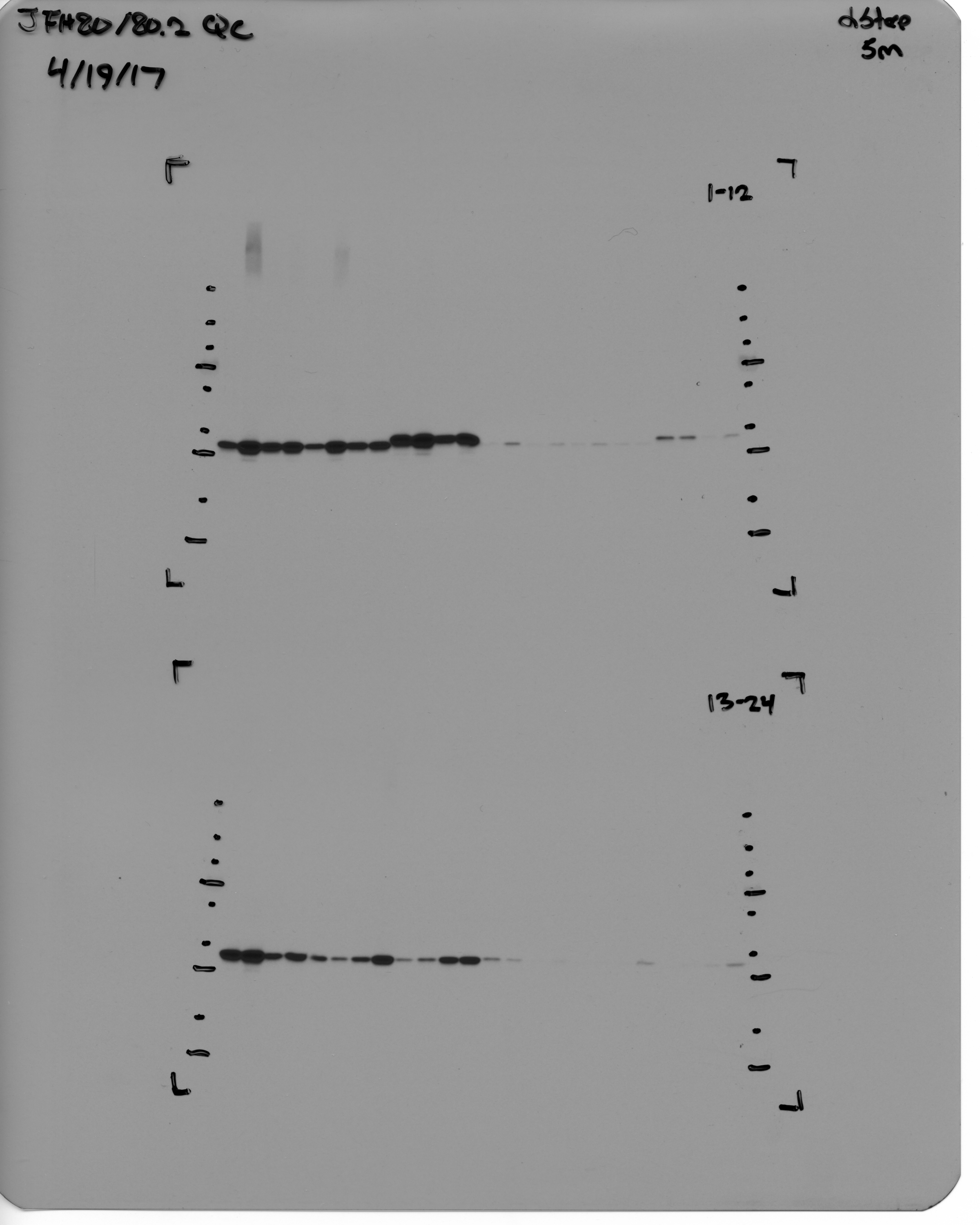

Supplement: Supplementary file 11 — Source Data [file 41467_2023_41442_MOESM11_ESM.zip › Haas_SourceData/Western Blot Scans (Supp Fig 3)/THP-1/JFH080_JFH080.2 - Strep - 5m.tif]

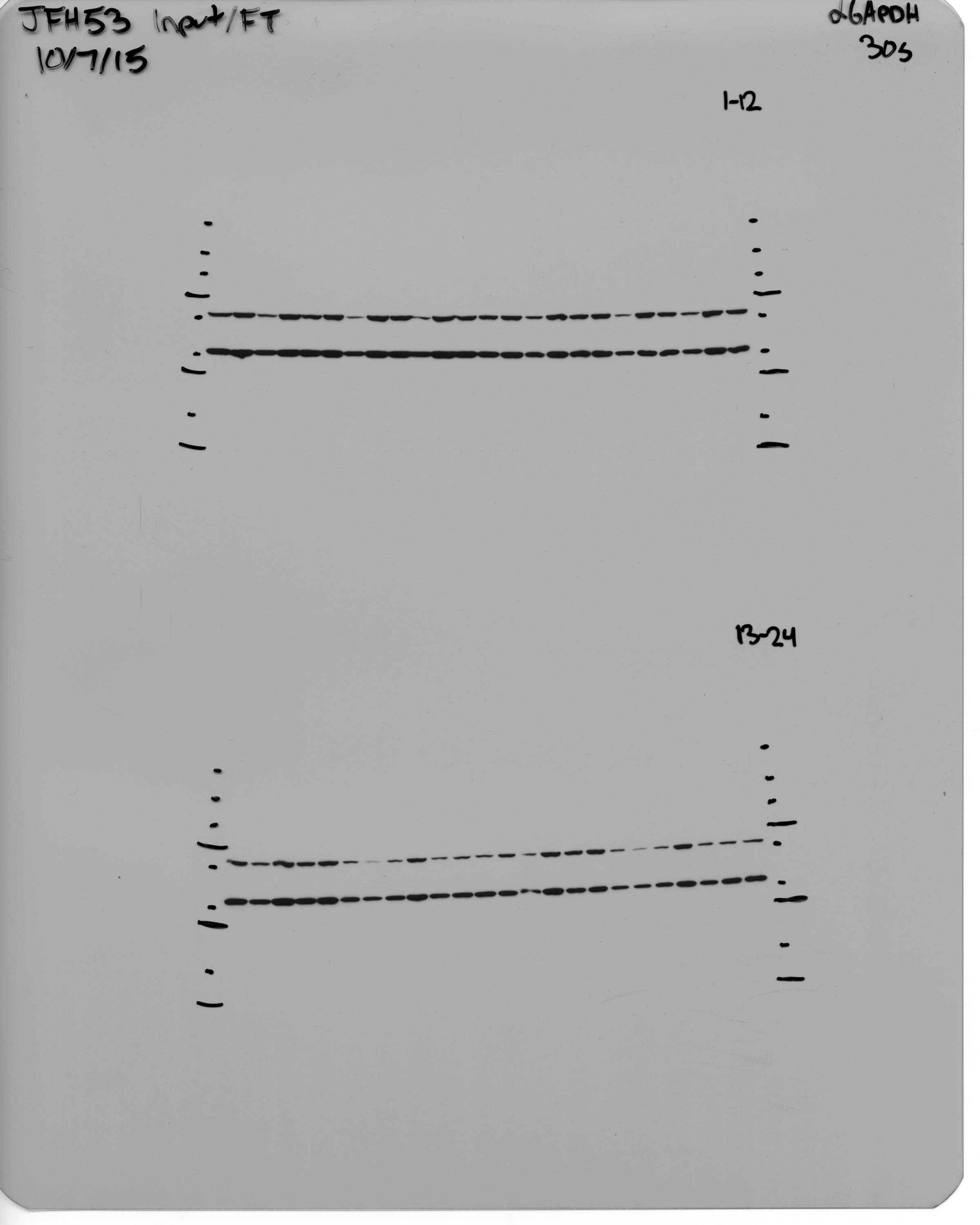

Supplement: Supplementary file 11 — Source Data [file 41467_2023_41442_MOESM11_ESM.zip › Haas_SourceData/Western Blot Scans (Supp Fig 3)/THP-1/JFH053 - GAPDH - 30s.tif]

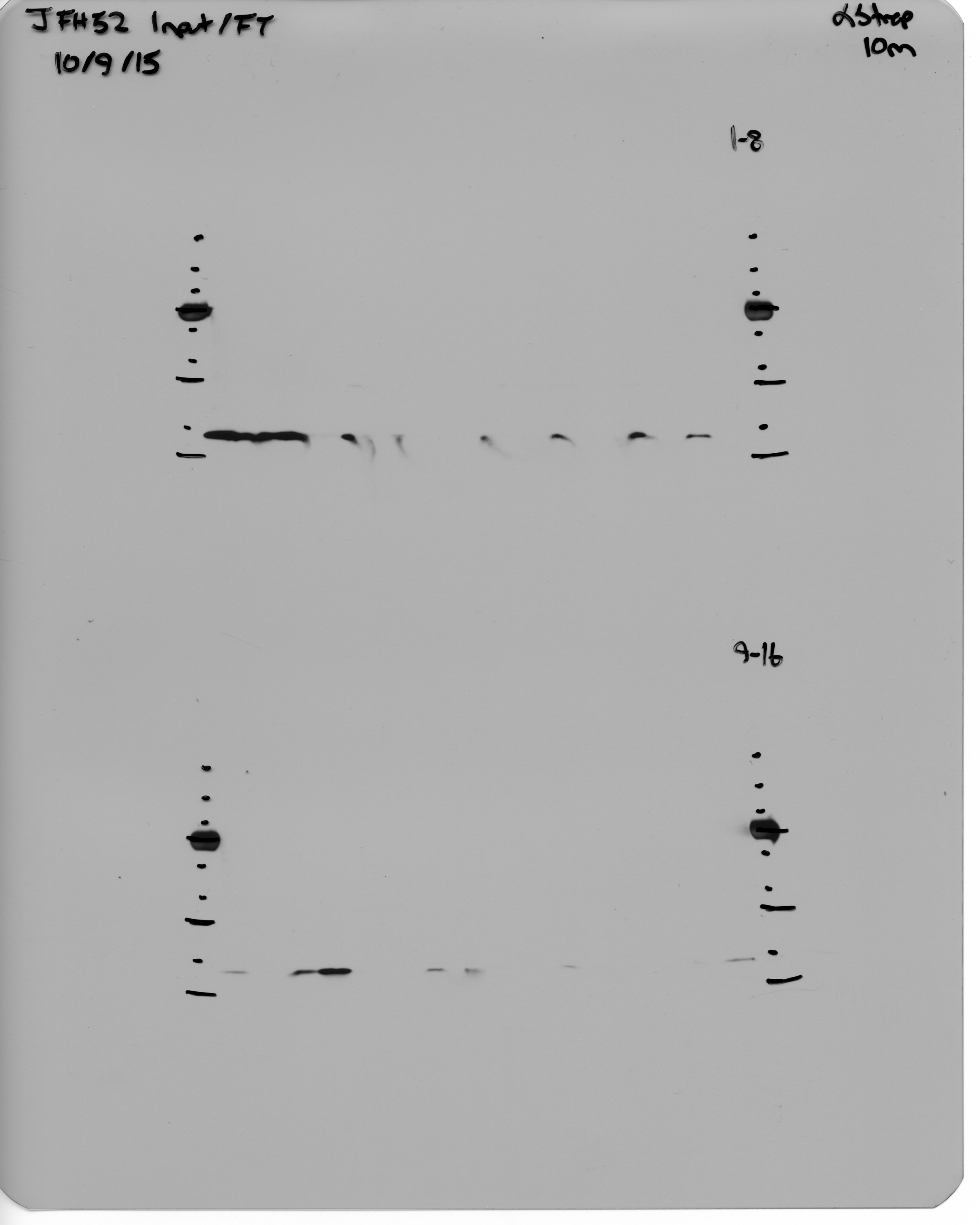

Supplement: Supplementary file 11 — Source Data [file 41467_2023_41442_MOESM11_ESM.zip › Haas_SourceData/Western Blot Scans (Supp Fig 3)/THP-1/JFH052 - Strep - 10m.tif]

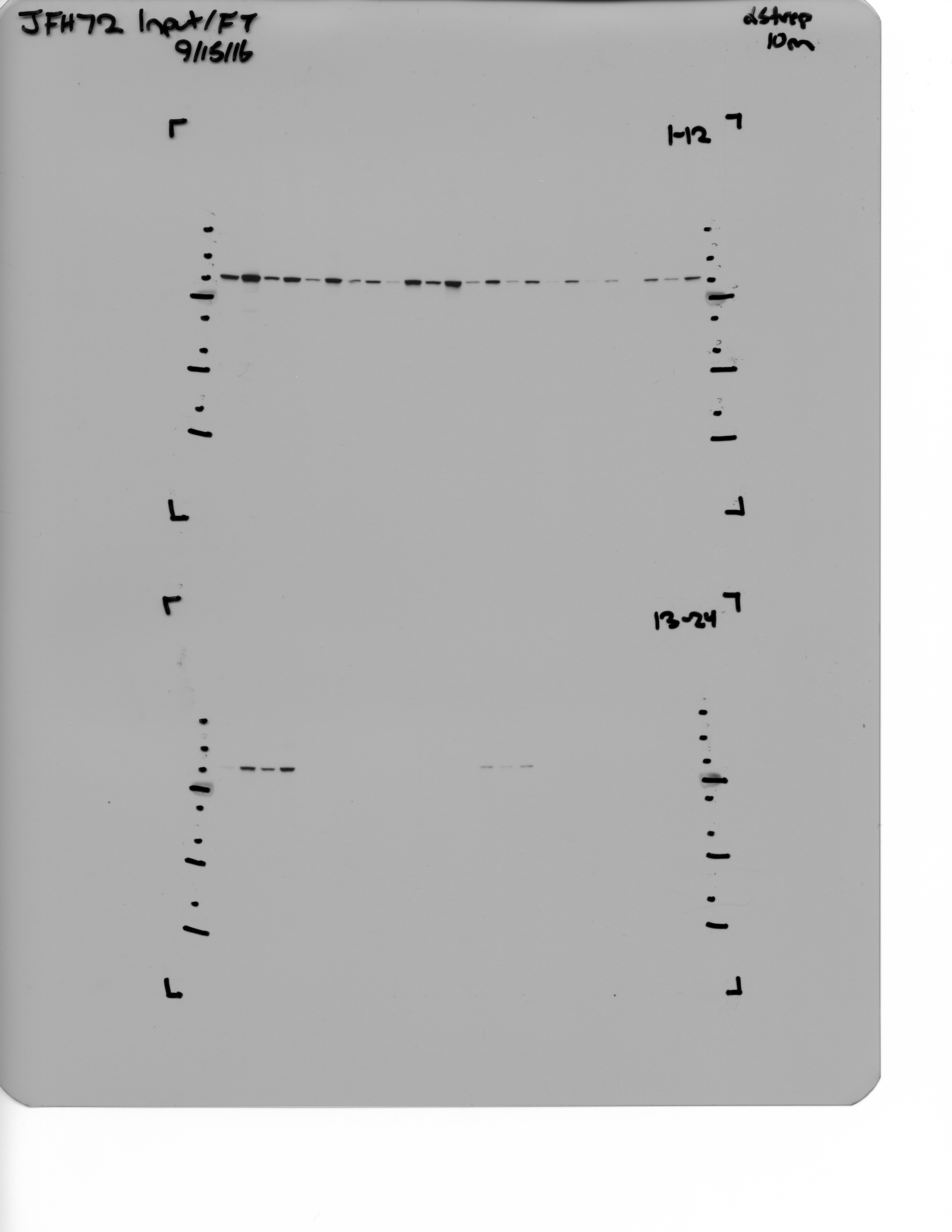

Supplement: Supplementary file 11 — Source Data [file 41467_2023_41442_MOESM11_ESM.zip › Haas_SourceData/Western Blot Scans (Supp Fig 3)/THP-1/JFH072 - Strep - 10m.tif]

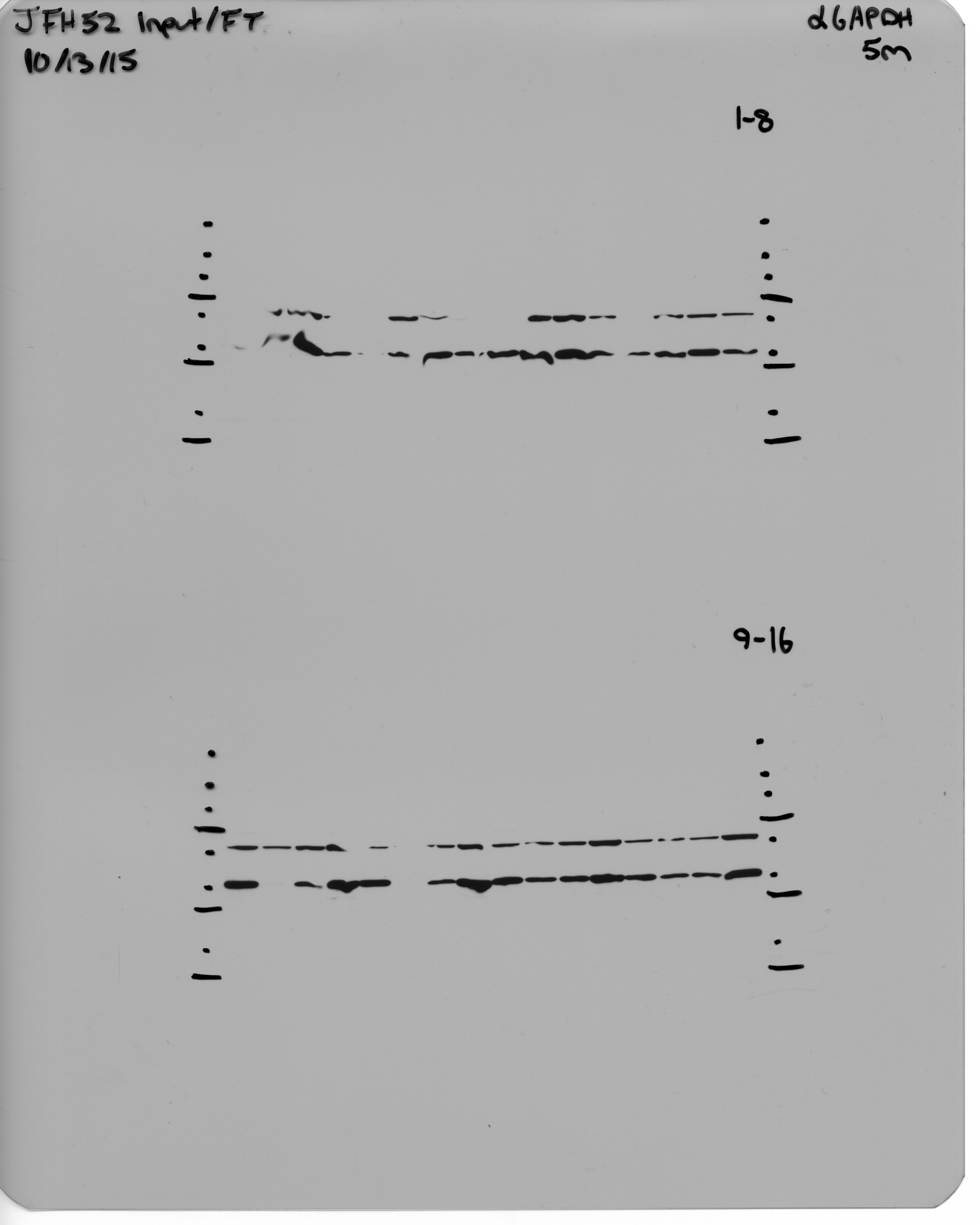

Supplement: Supplementary file 11 — Source Data [file 41467_2023_41442_MOESM11_ESM.zip › Haas_SourceData/Western Blot Scans (Supp Fig 3)/THP-1/JFH052 - GAPDH - 5m.tif]

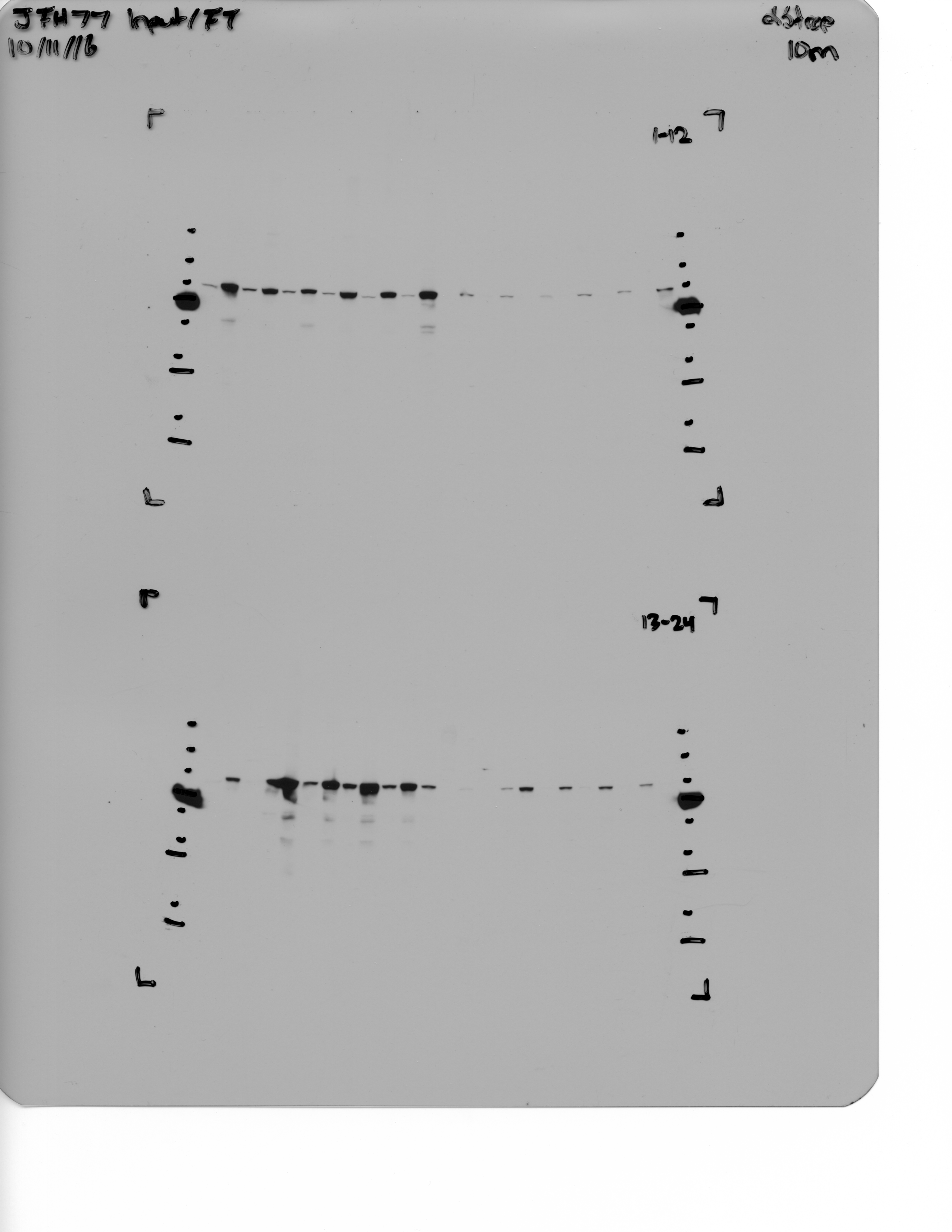

Supplement: Supplementary file 11 — Source Data [file 41467_2023_41442_MOESM11_ESM.zip › Haas_SourceData/Western Blot Scans (Supp Fig 3)/THP-1/JFH077 - Strep - 10m.tif]

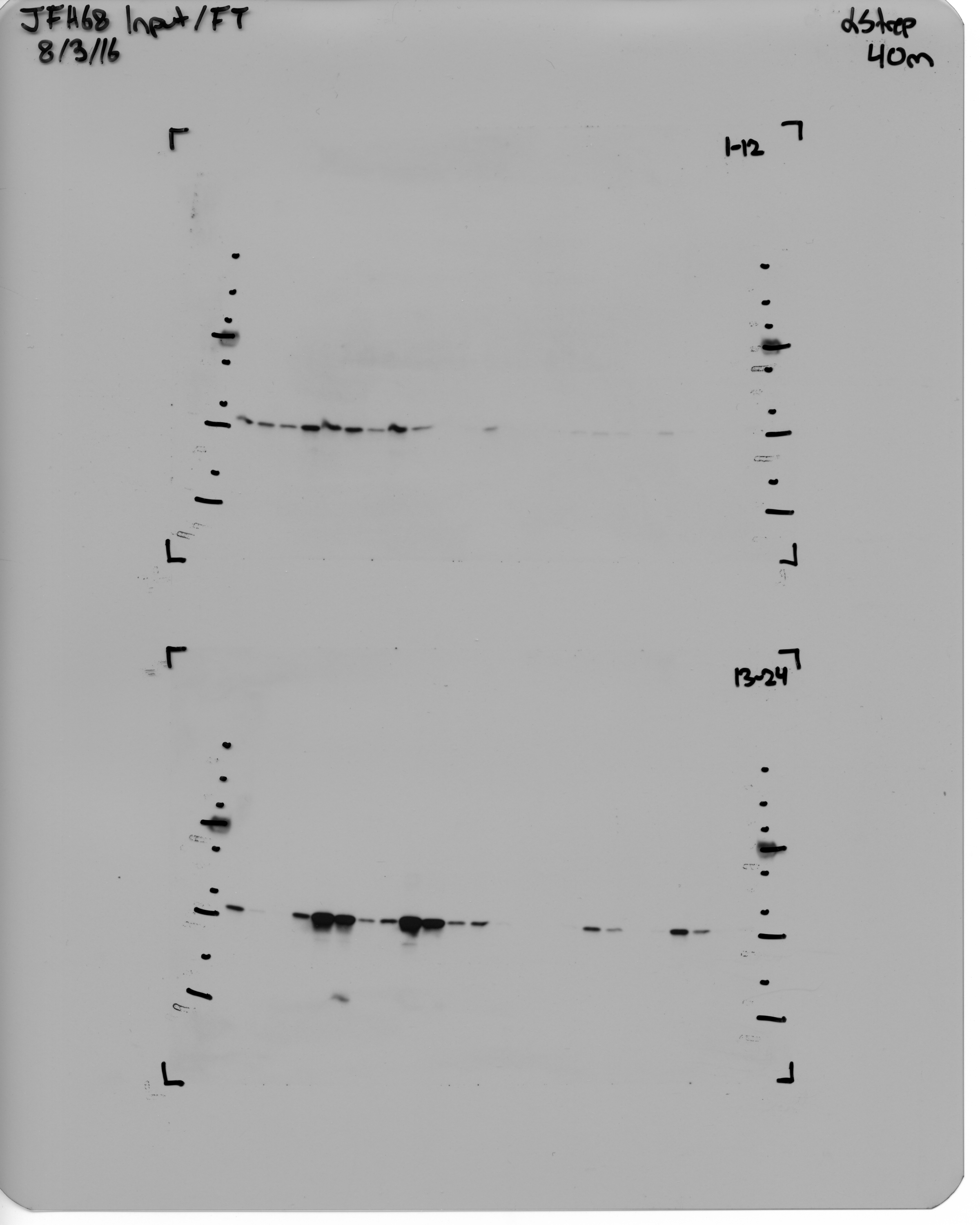

Supplement: Supplementary file 11 — Source Data [file 41467_2023_41442_MOESM11_ESM.zip › Haas_SourceData/Western Blot Scans (Supp Fig 3)/THP-1/JFH068 - Strep - 40m.tif]

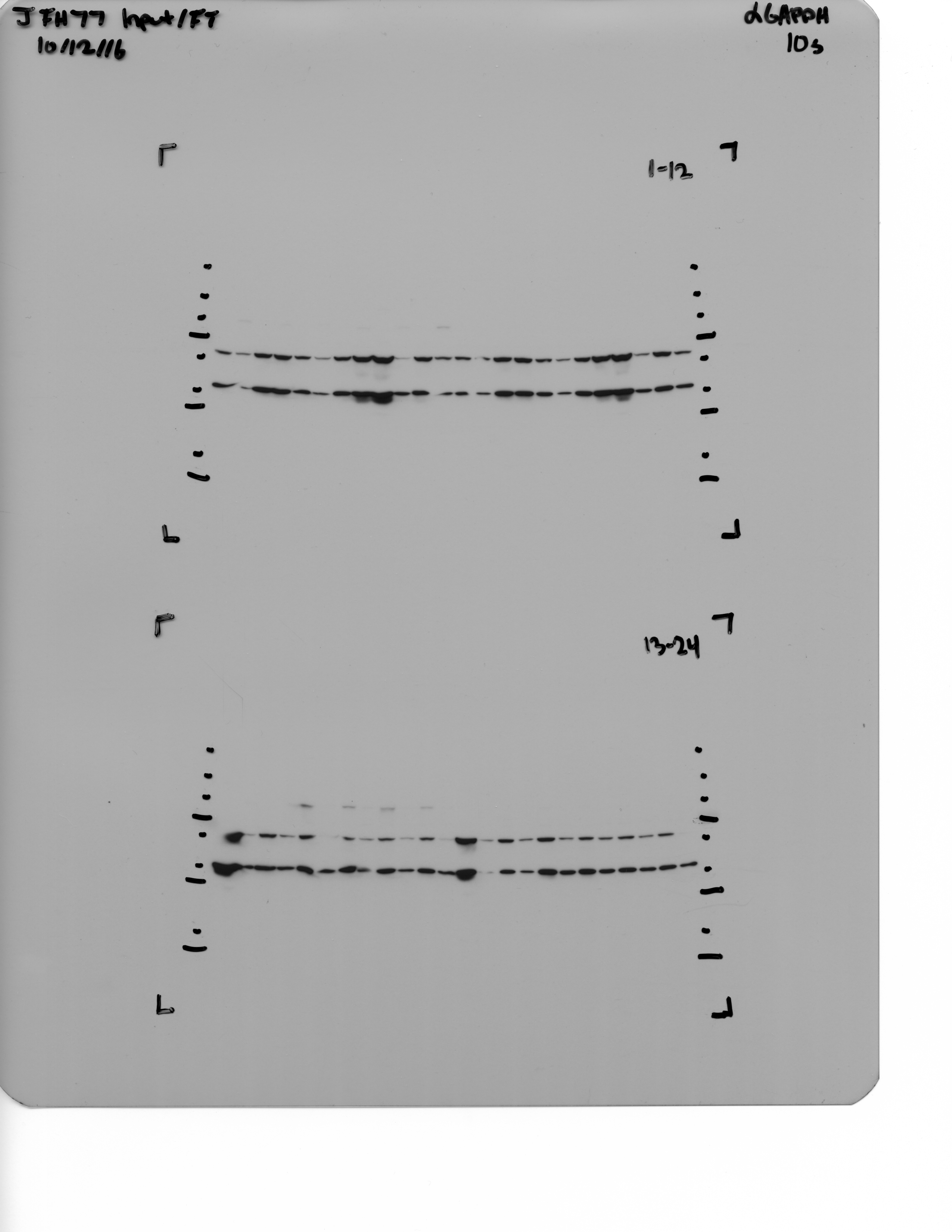

Supplement: Supplementary file 11 — Source Data [file 41467_2023_41442_MOESM11_ESM.zip › Haas_SourceData/Western Blot Scans (Supp Fig 3)/THP-1/JFH077 - GAPDH - 10s.tif]

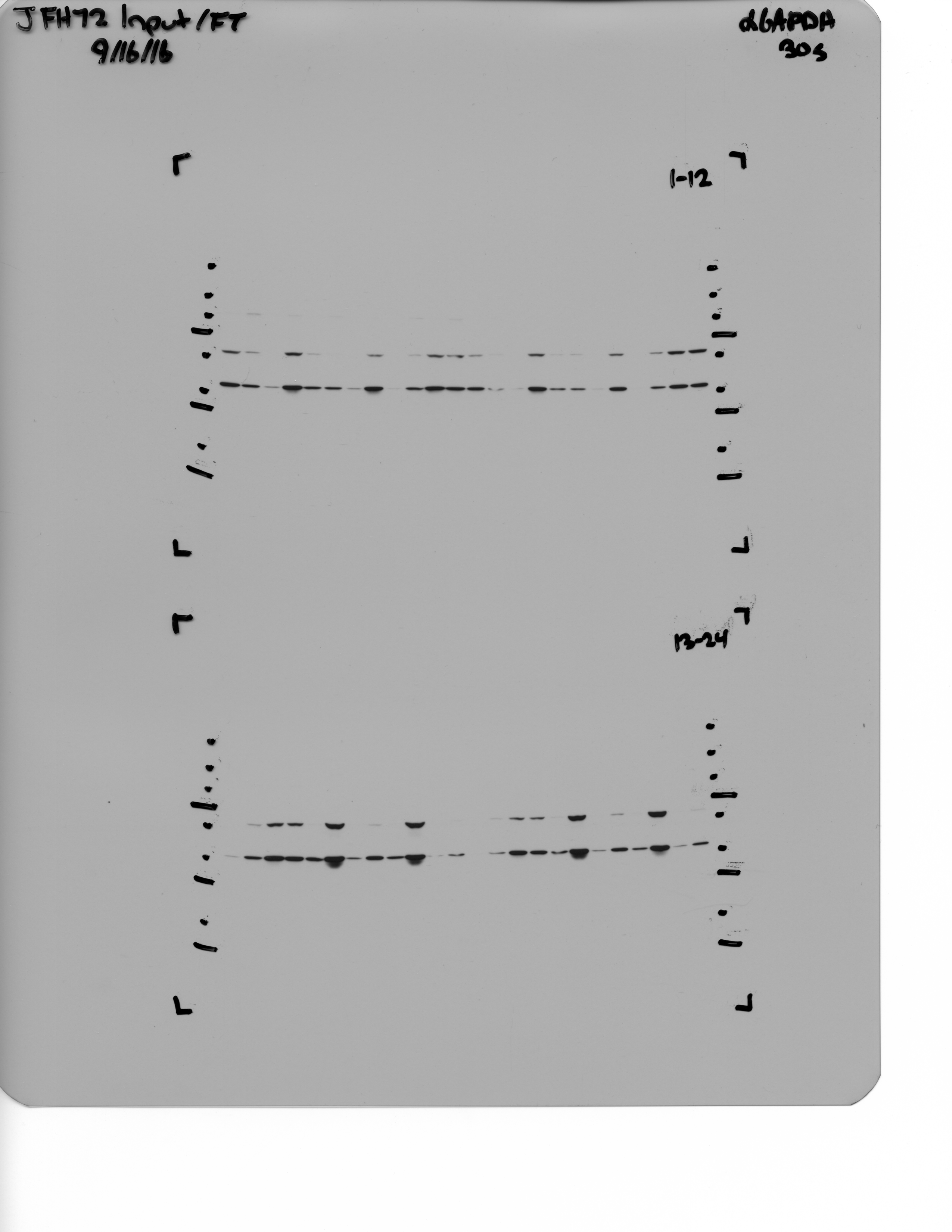

Supplement: Supplementary file 11 — Source Data [file 41467_2023_41442_MOESM11_ESM.zip › Haas_SourceData/Western Blot Scans (Supp Fig 3)/THP-1/JFH072 - GAPDH - 30s.tif]

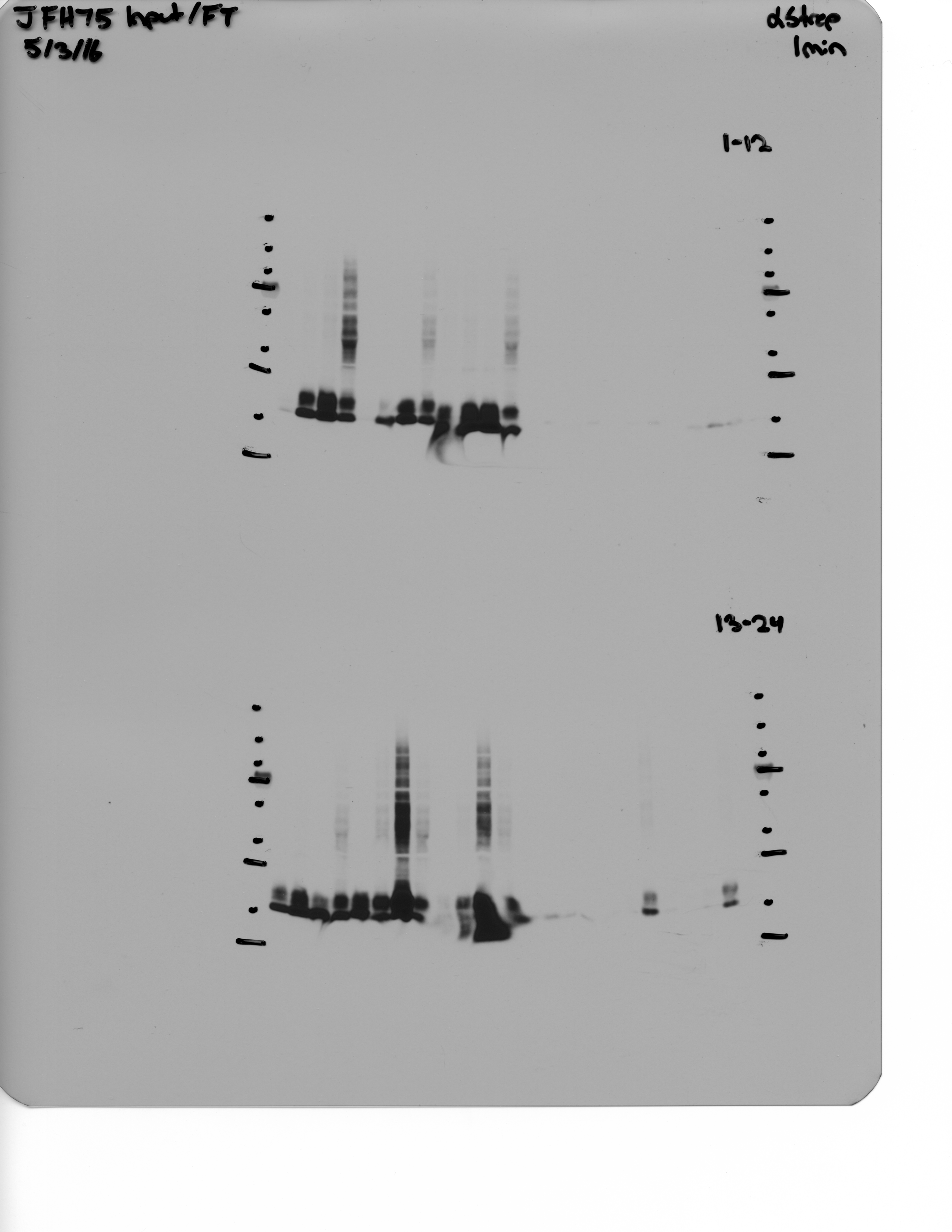

Supplement: Supplementary file 11 — Source Data [file 41467_2023_41442_MOESM11_ESM.zip › Haas_SourceData/Western Blot Scans (Supp Fig 3)/THP-1/JFH075 - Strep - 1m.tif]

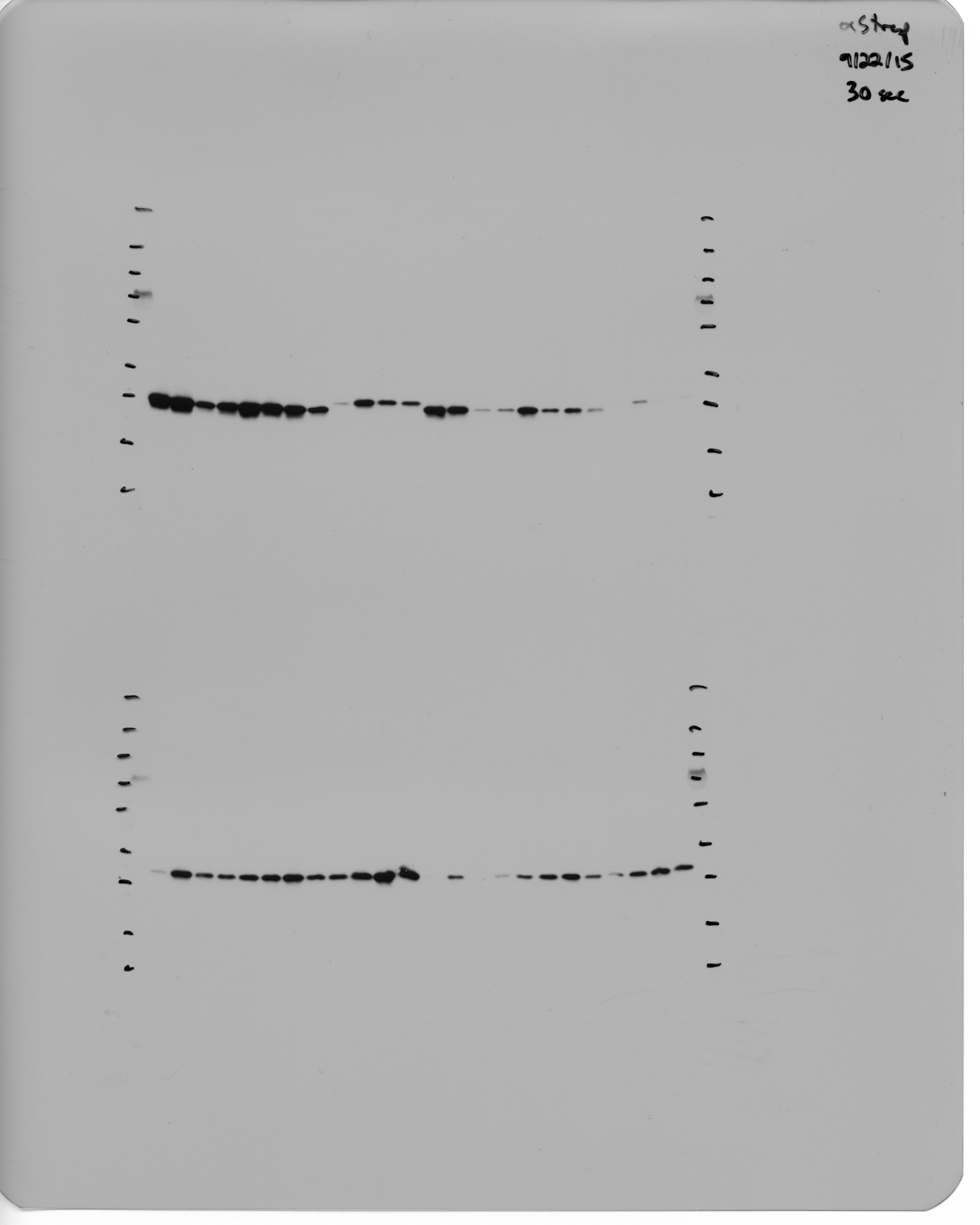

Supplement: Supplementary file 11 — Source Data [file 41467_2023_41442_MOESM11_ESM.zip › Haas_SourceData/Western Blot Scans (Supp Fig 3)/THP-1/JFH049 - Strep - 30s.tif]

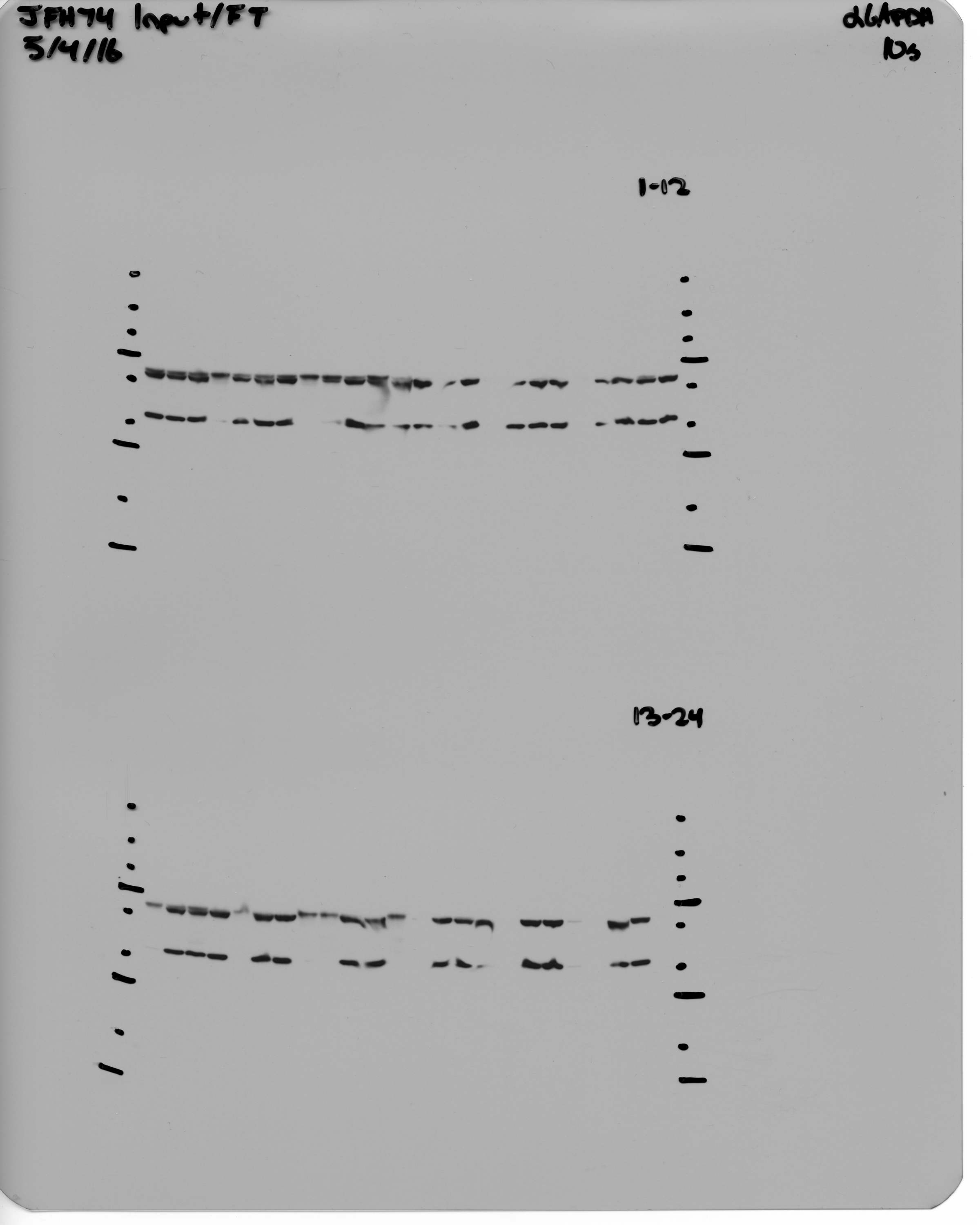

Supplement: Supplementary file 11 — Source Data [file 41467_2023_41442_MOESM11_ESM.zip › Haas_SourceData/Western Blot Scans (Supp Fig 3)/THP-1/JFH074 - GAPDH - 10s.tif]

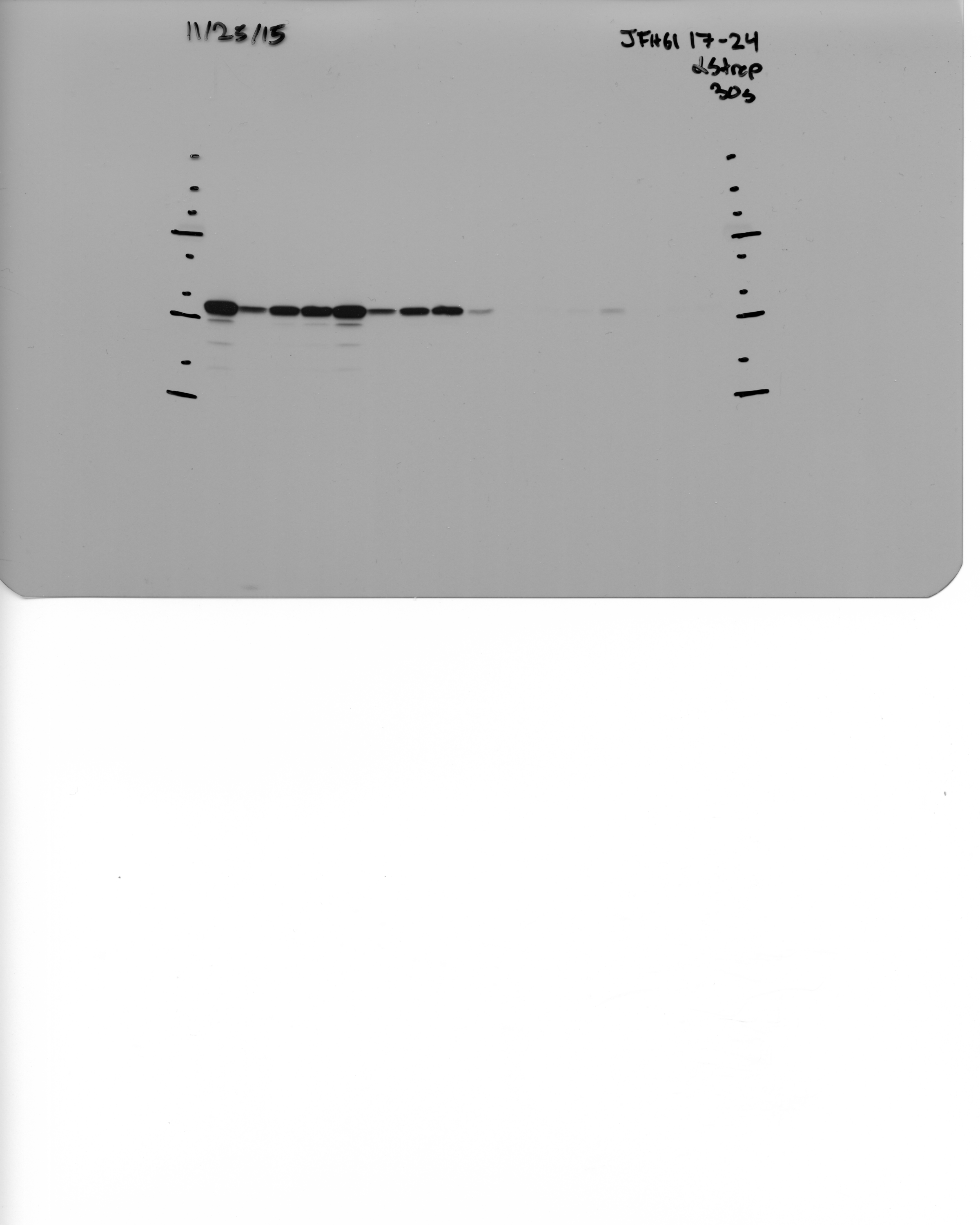

Supplement: Supplementary file 11 — Source Data [file 41467_2023_41442_MOESM11_ESM.zip › Haas_SourceData/Western Blot Scans (Supp Fig 3)/THP-1/JFH061 - Strep - 30s.tif]

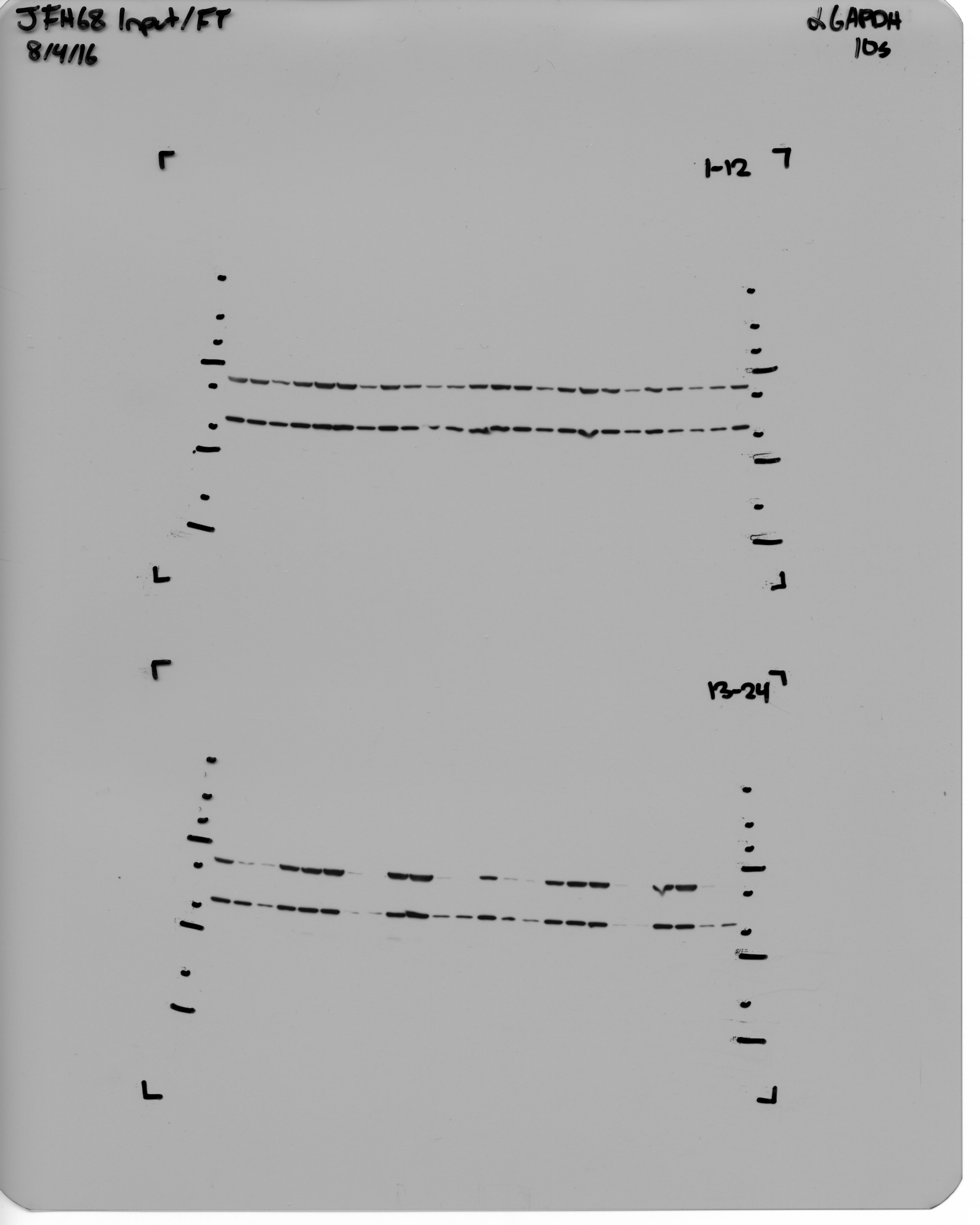

Supplement: Supplementary file 11 — Source Data [file 41467_2023_41442_MOESM11_ESM.zip › Haas_SourceData/Western Blot Scans (Supp Fig 3)/THP-1/JFH068 - GAPDH - 10s.tif]

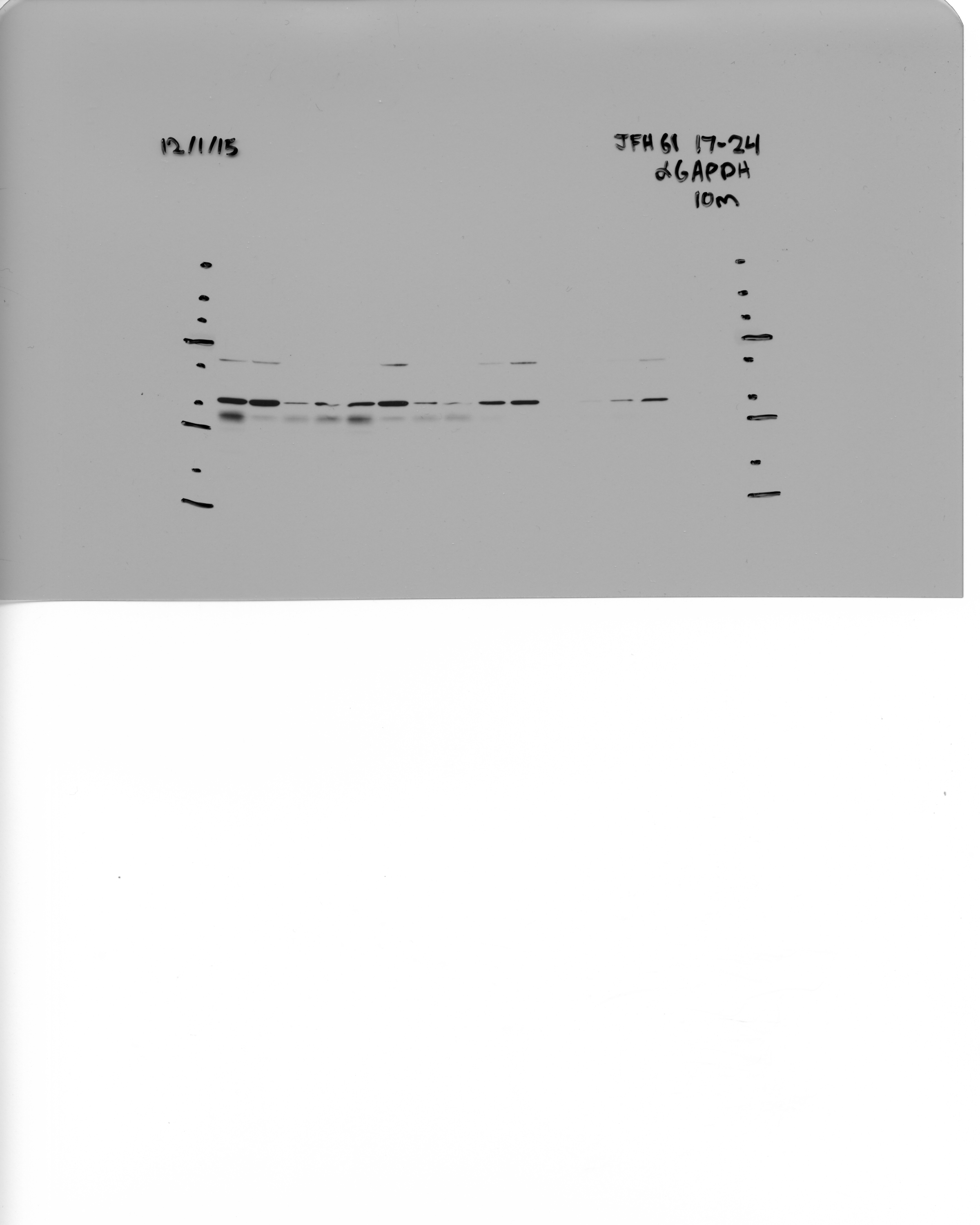

Supplement: Supplementary file 11 — Source Data [file 41467_2023_41442_MOESM11_ESM.zip › Haas_SourceData/Western Blot Scans (Supp Fig 3)/THP-1/JFH061 - GAPDH - 10m.tif]

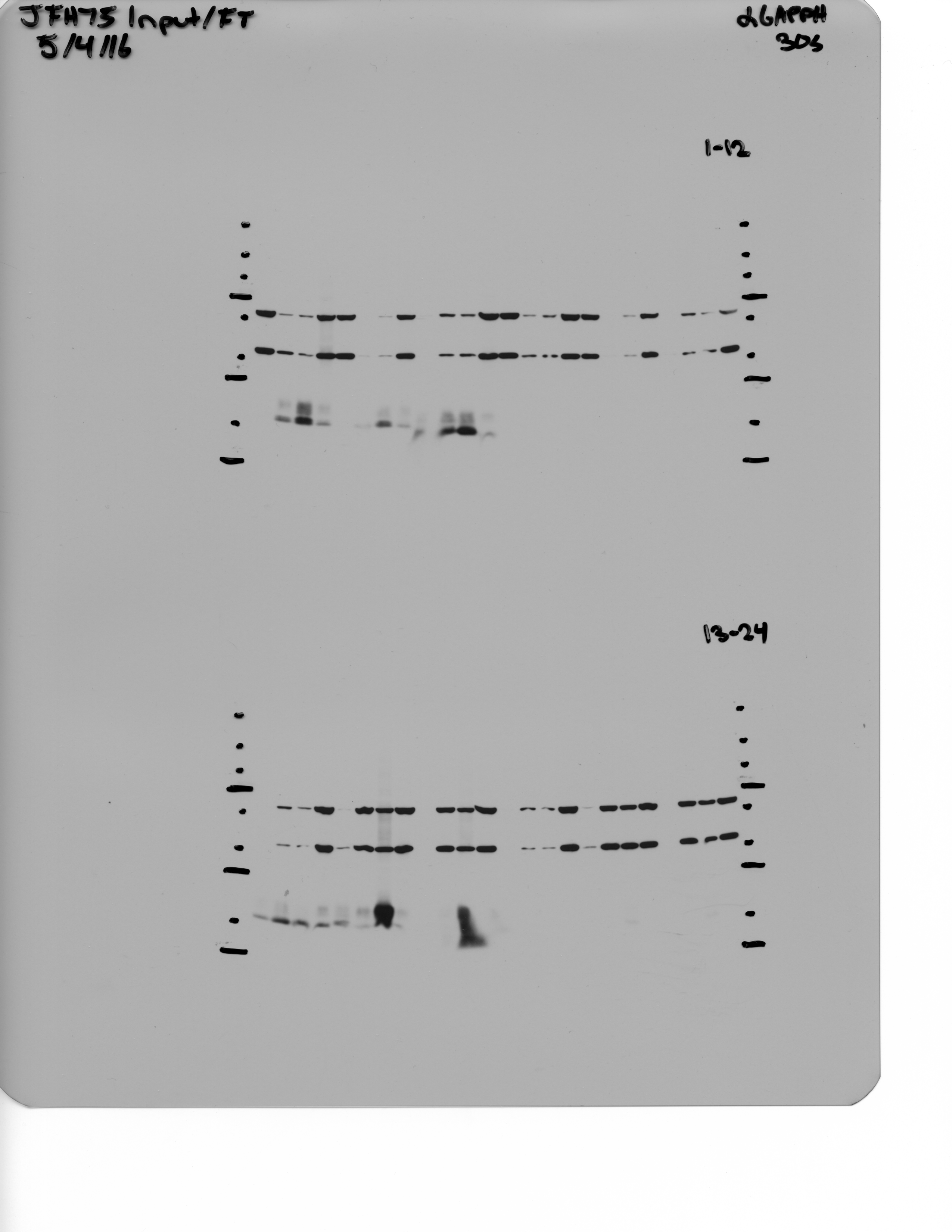

Supplement: Supplementary file 11 — Source Data [file 41467_2023_41442_MOESM11_ESM.zip › Haas_SourceData/Western Blot Scans (Supp Fig 3)/THP-1/JFH075 - GAPDH - 30s.tif]

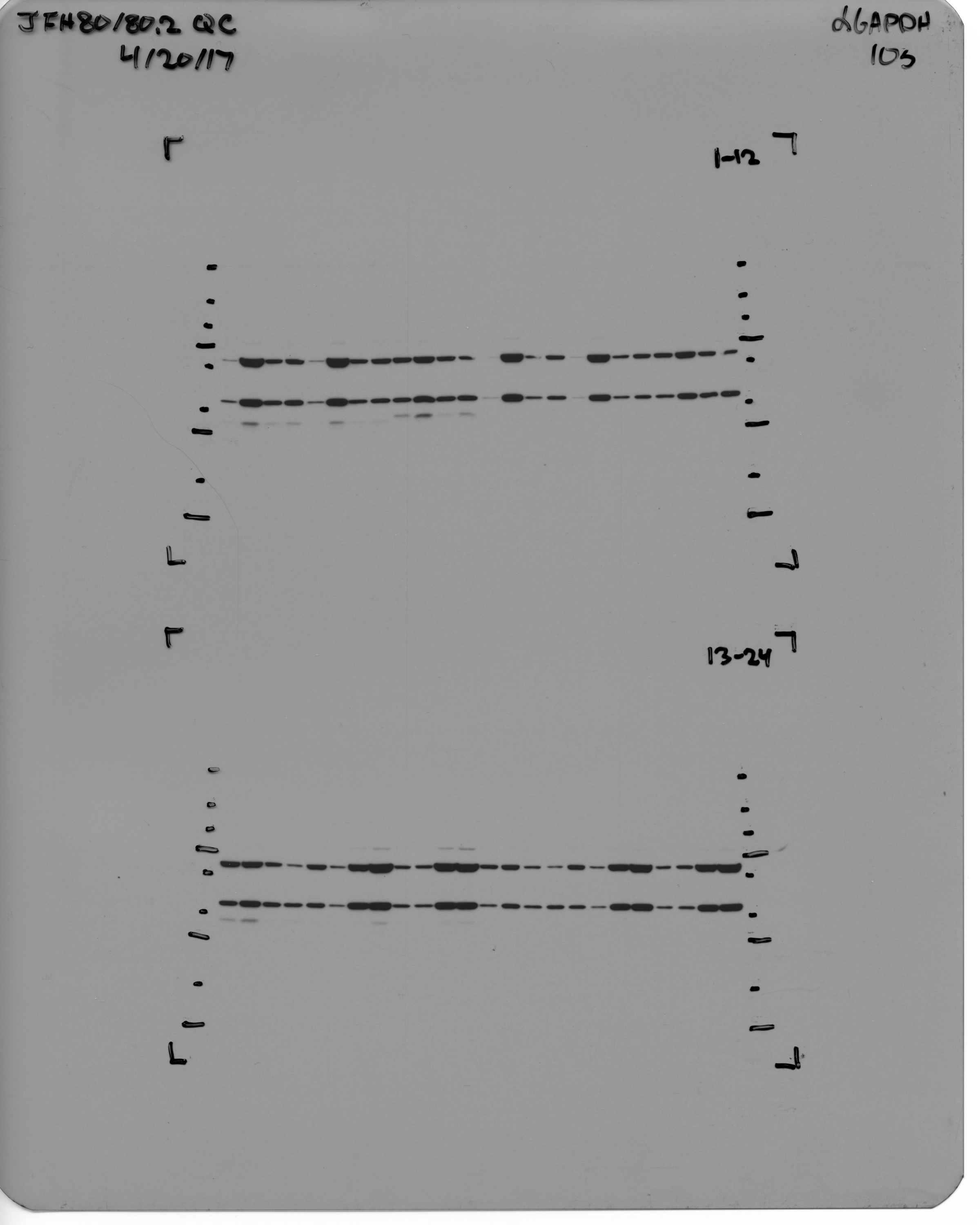

Supplement: Supplementary file 11 — Source Data [file 41467_2023_41442_MOESM11_ESM.zip › Haas_SourceData/Western Blot Scans (Supp Fig 3)/THP-1/JFH080_JFH080.2 - GAPDH - 10s.tif]

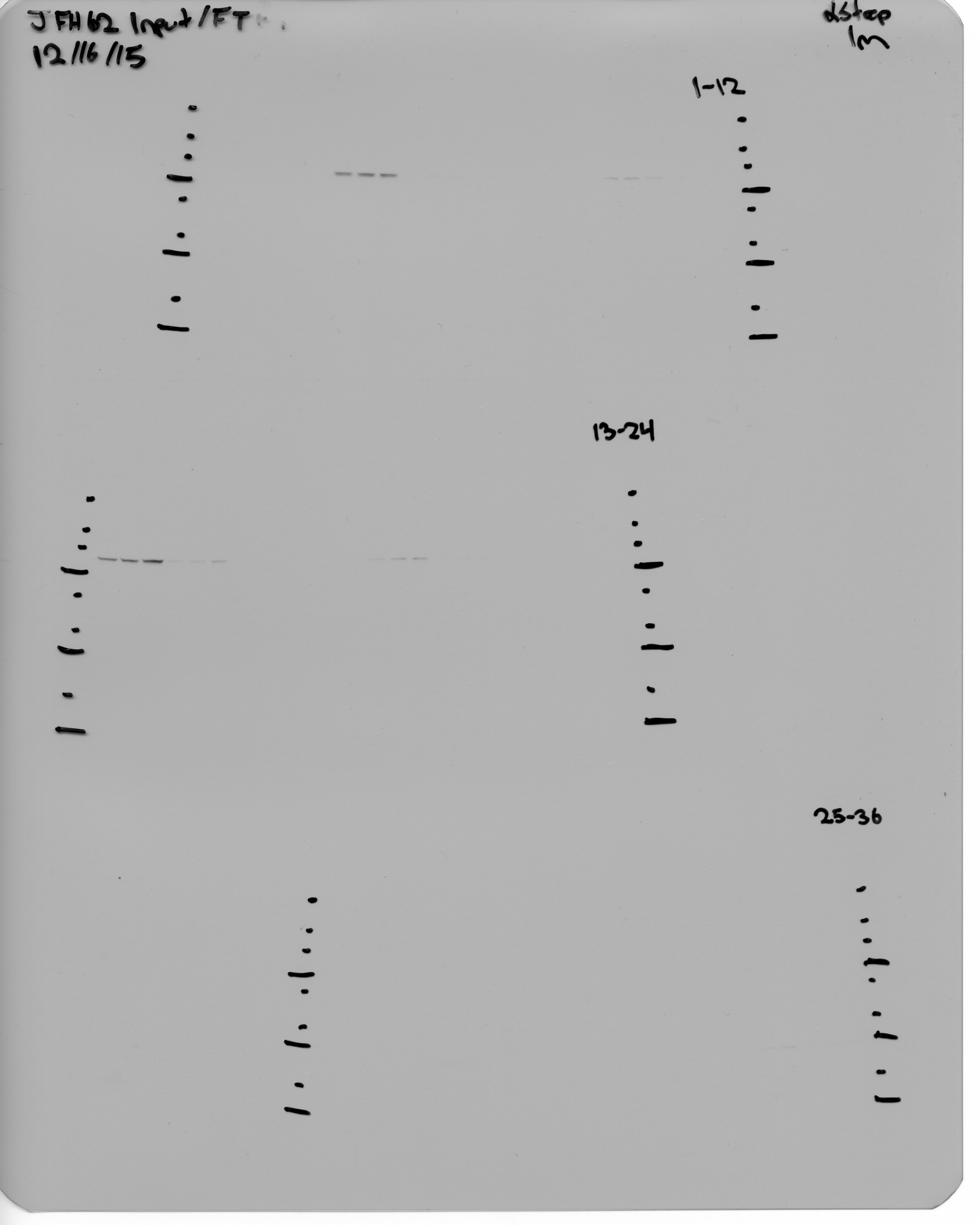

Supplement: Supplementary file 11 — Source Data [file 41467_2023_41442_MOESM11_ESM.zip › Haas_SourceData/Western Blot Scans (Supp Fig 3)/A549/JFH062 - Strep - 1m.tif]

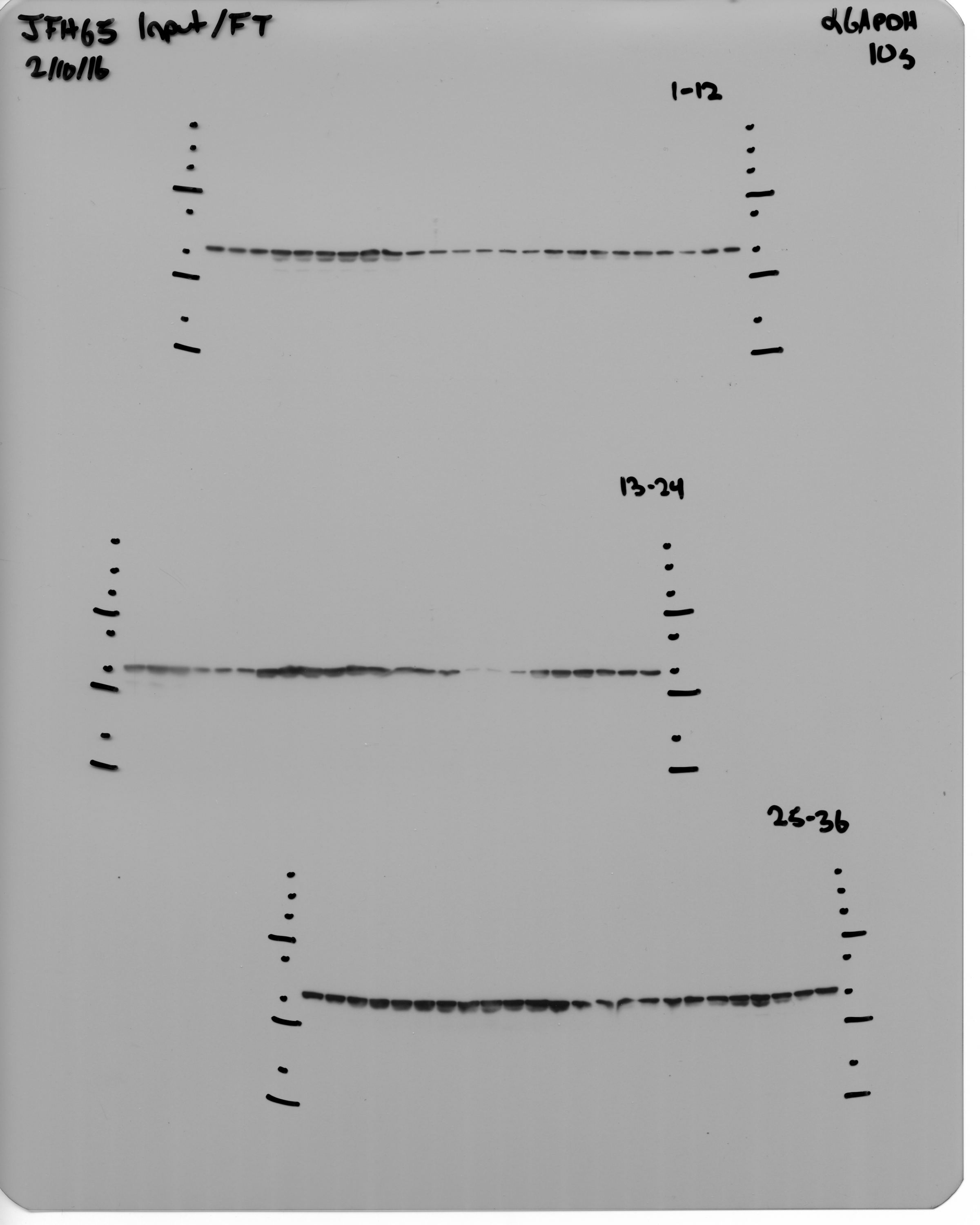

Supplement: Supplementary file 11 — Source Data [file 41467_2023_41442_MOESM11_ESM.zip › Haas_SourceData/Western Blot Scans (Supp Fig 3)/A549/JFH065 - GAPDH - 10s.tif]

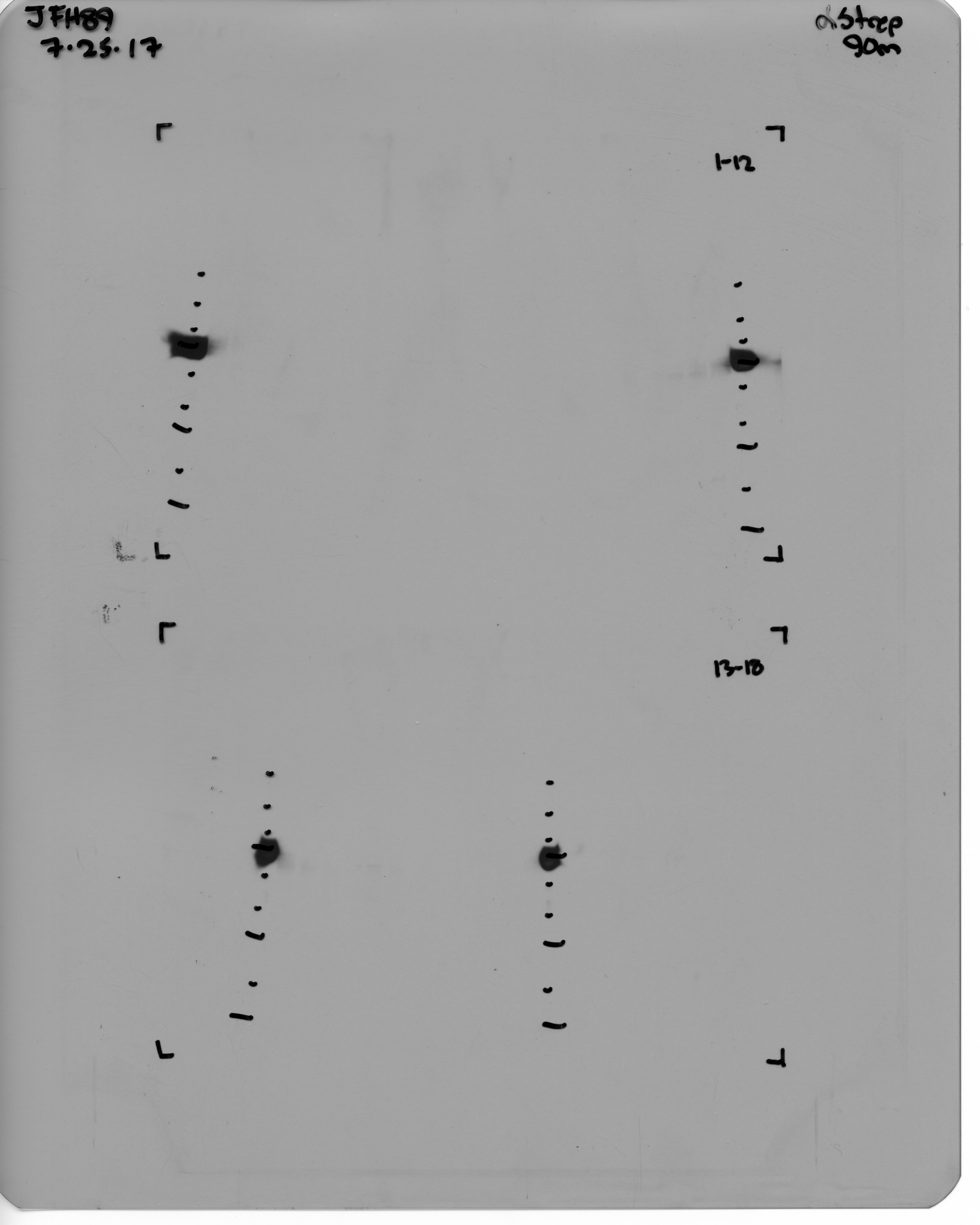

Supplement: Supplementary file 11 — Source Data [file 41467_2023_41442_MOESM11_ESM.zip › Haas_SourceData/Western Blot Scans (Supp Fig 3)/A549/JFH089 - Strep - 90m.tif]

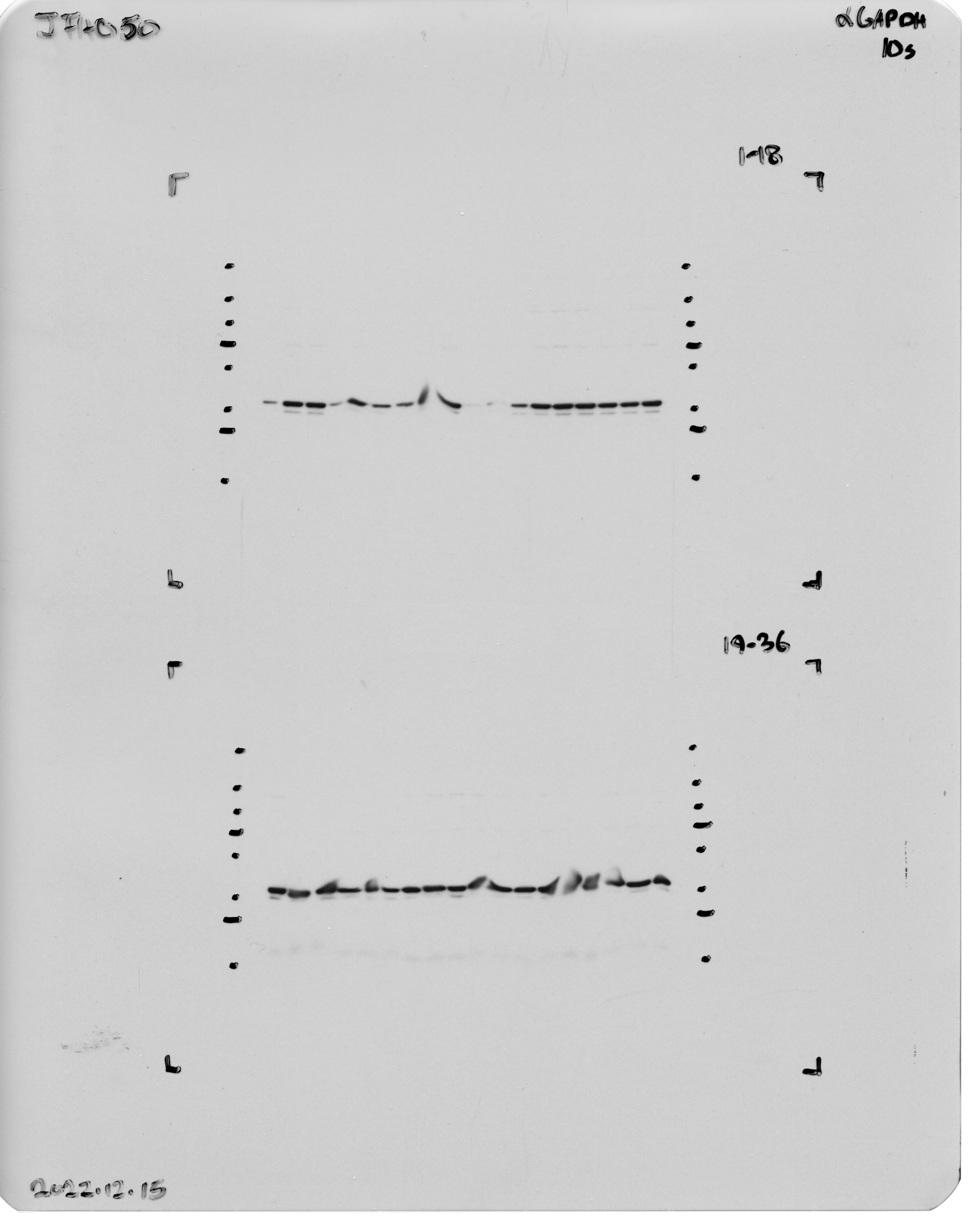

Supplement: Supplementary file 11 — Source Data [file 41467_2023_41442_MOESM11_ESM.zip › Haas_SourceData/Western Blot Scans (Supp Fig 3)/A549/JFH050 - GAPDH - 10s.tif]

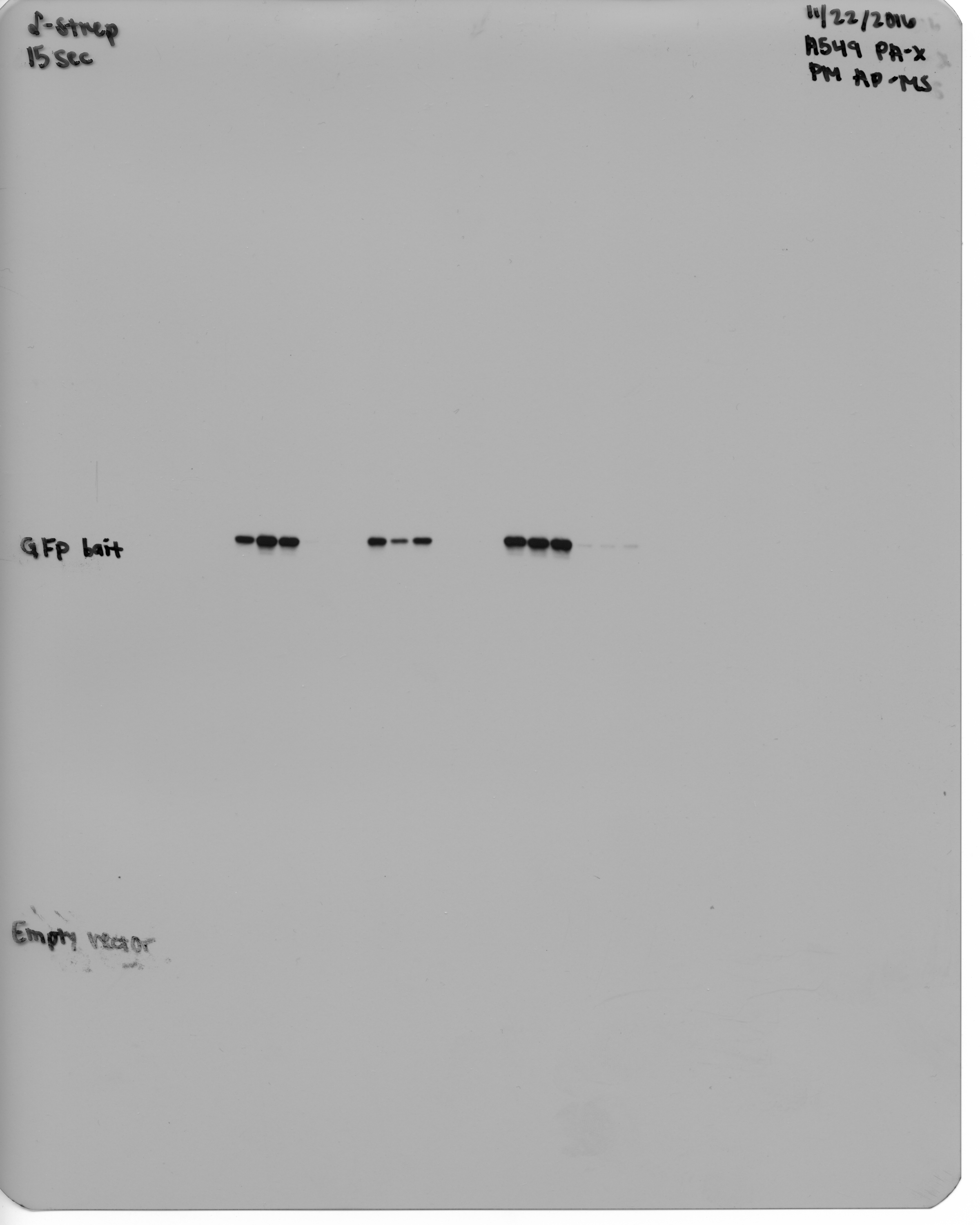

Supplement: Supplementary file 11 — Source Data [file 41467_2023_41442_MOESM11_ESM.zip › Haas_SourceData/Western Blot Scans (Supp Fig 3)/A549/JFH081 - GFP-Strep - 15s.tif]

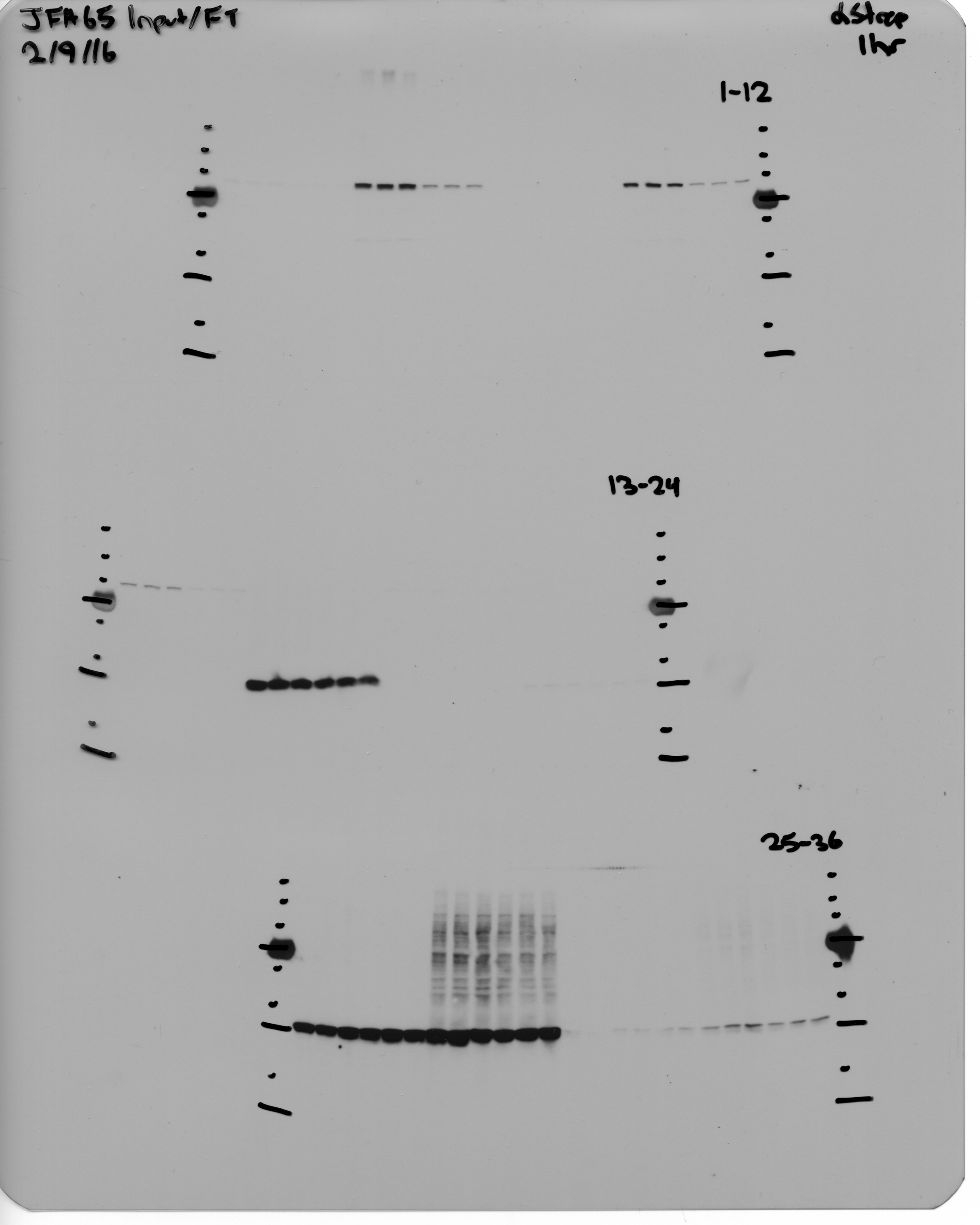

Supplement: Supplementary file 11 — Source Data [file 41467_2023_41442_MOESM11_ESM.zip › Haas_SourceData/Western Blot Scans (Supp Fig 3)/A549/JFH065 - Strep - 60m.tif]

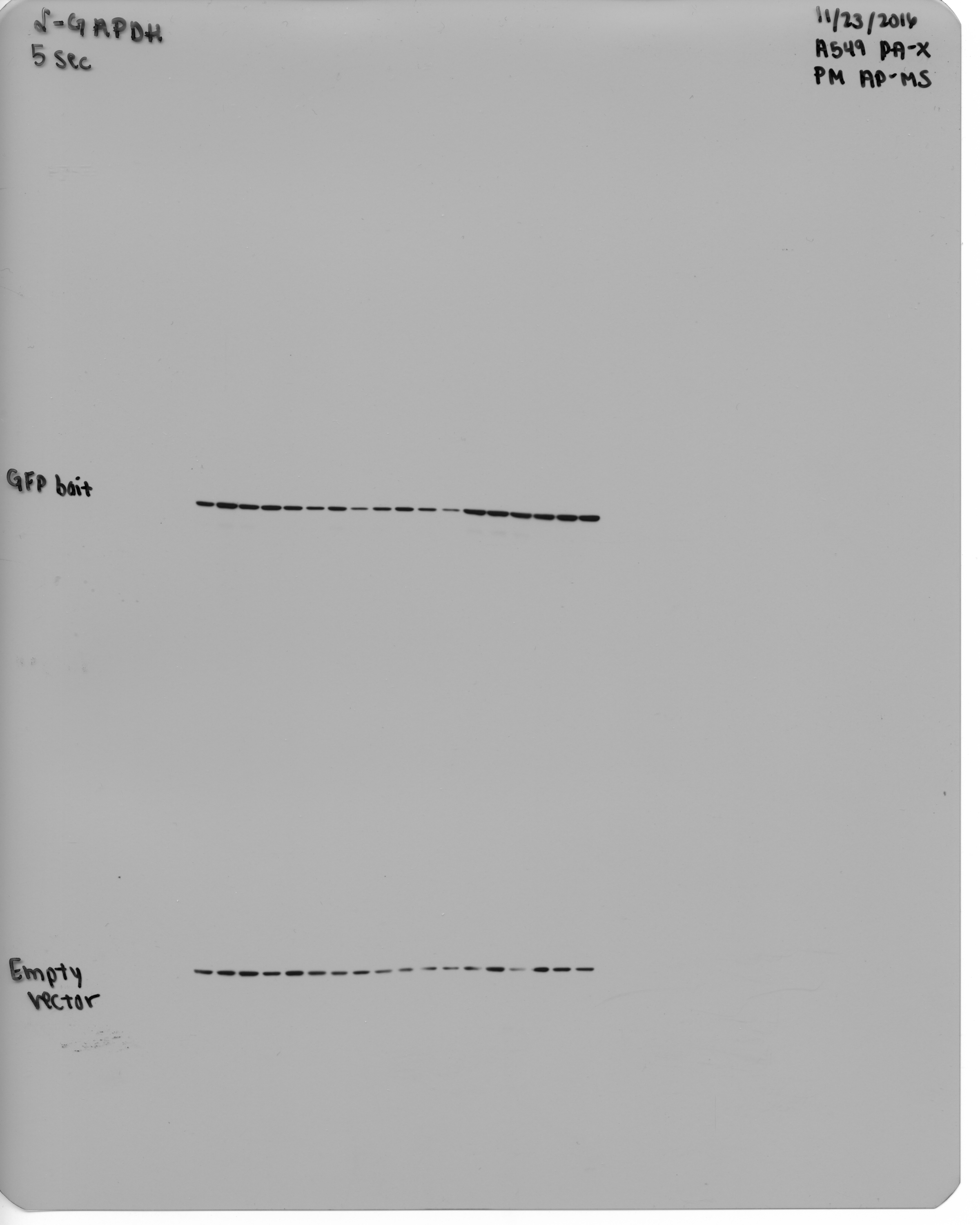

Supplement: Supplementary file 11 — Source Data [file 41467_2023_41442_MOESM11_ESM.zip › Haas_SourceData/Western Blot Scans (Supp Fig 3)/A549/JFH081 - GFP-GAPDH - 5s.tif]

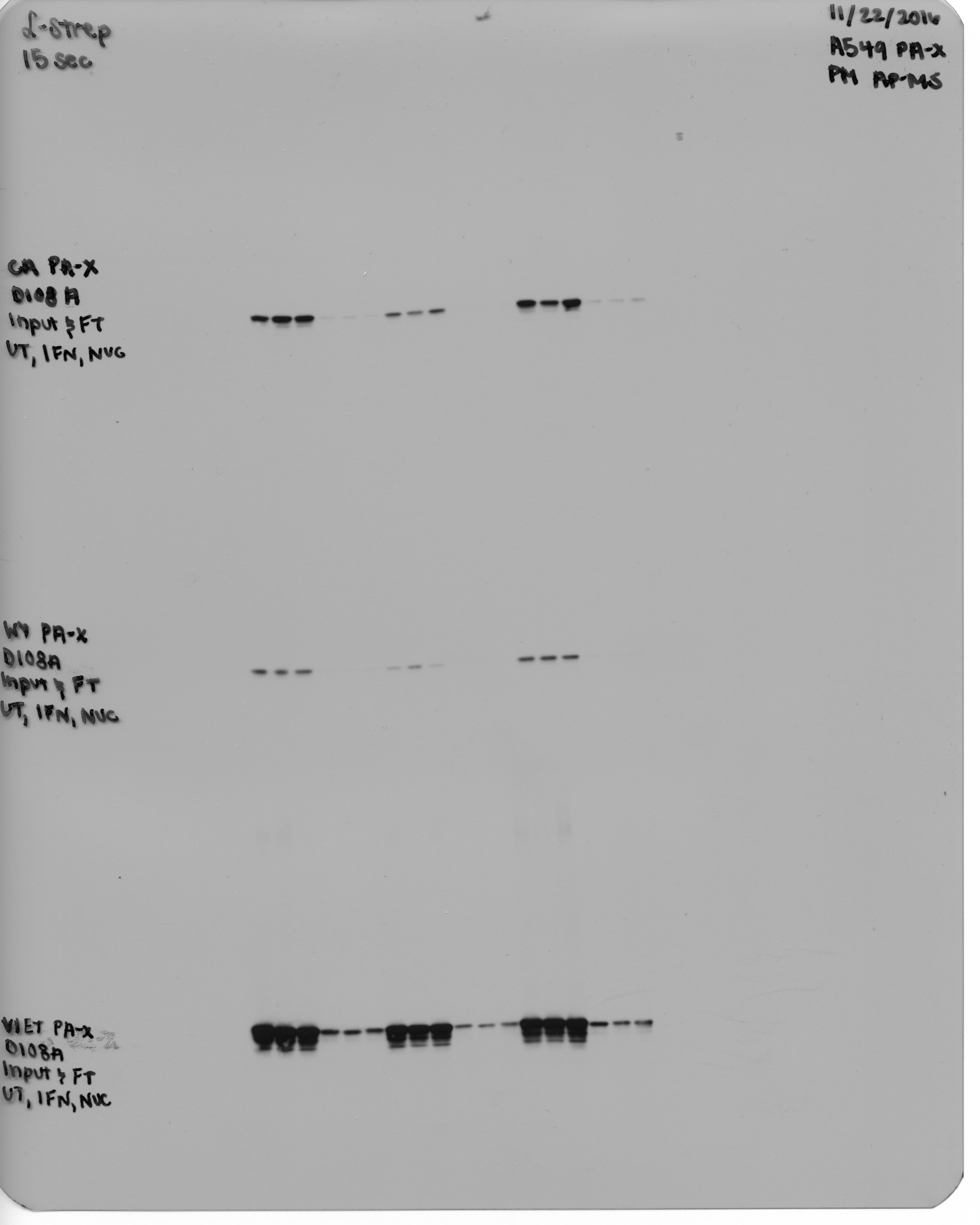

Supplement: Supplementary file 11 — Source Data [file 41467_2023_41442_MOESM11_ESM.zip › Haas_SourceData/Western Blot Scans (Supp Fig 3)/A549/JFH081 - Strep - 15s.tif]

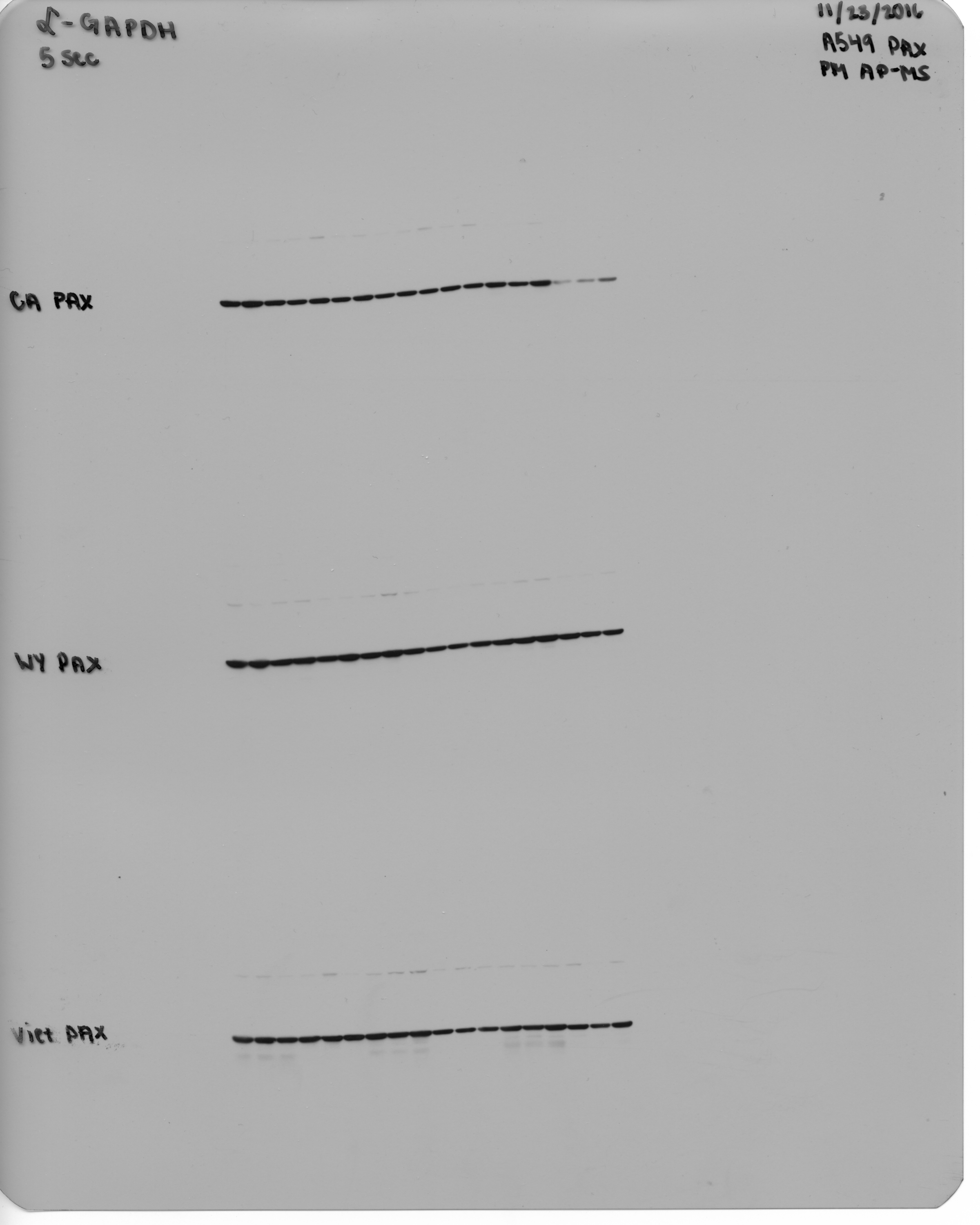

Supplement: Supplementary file 11 — Source Data [file 41467_2023_41442_MOESM11_ESM.zip › Haas_SourceData/Western Blot Scans (Supp Fig 3)/A549/JFH081 - GAPDH - 5s.tif]

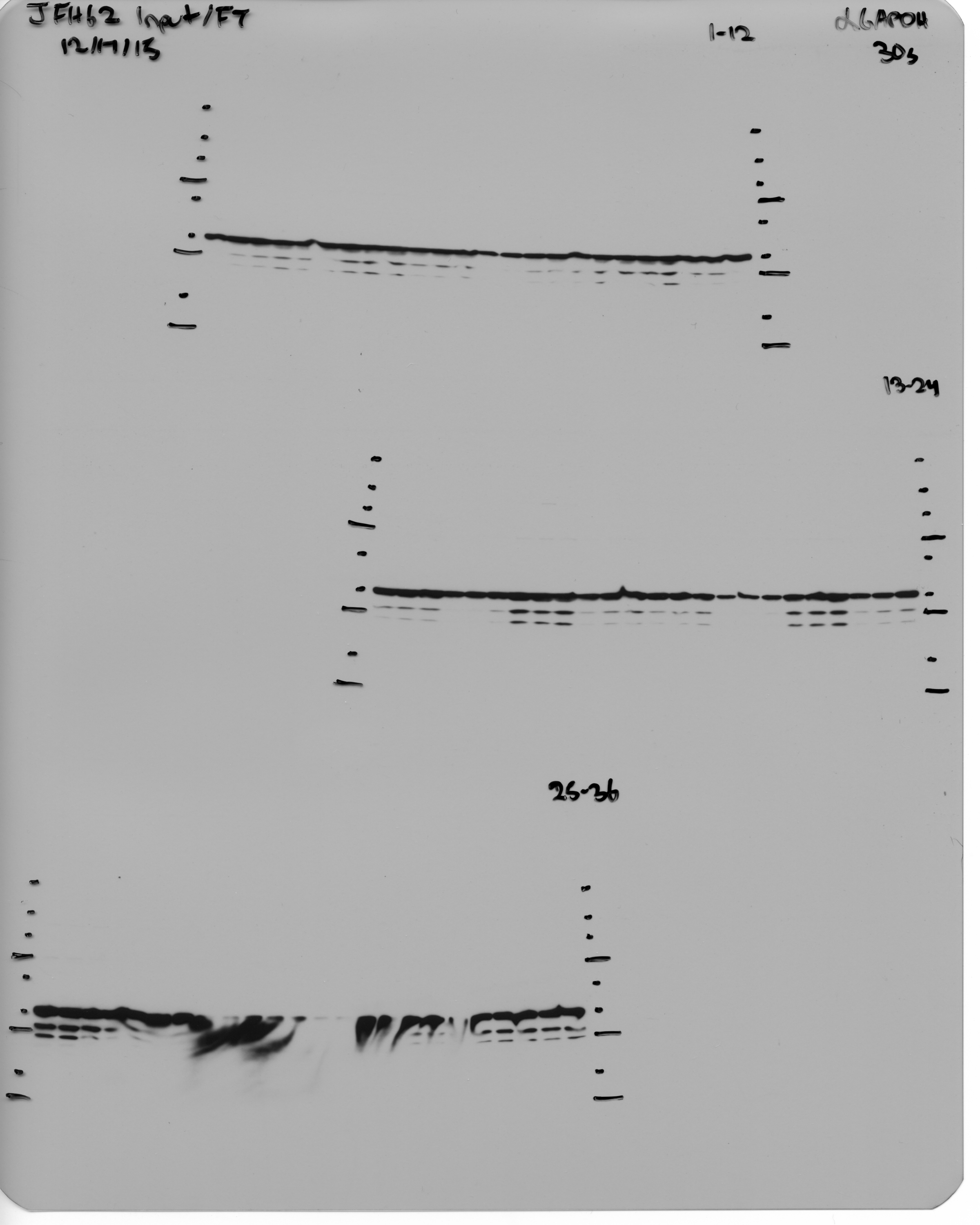

Supplement: Supplementary file 11 — Source Data [file 41467_2023_41442_MOESM11_ESM.zip › Haas_SourceData/Western Blot Scans (Supp Fig 3)/A549/JFH062 - GAPDH - 30s.tif]

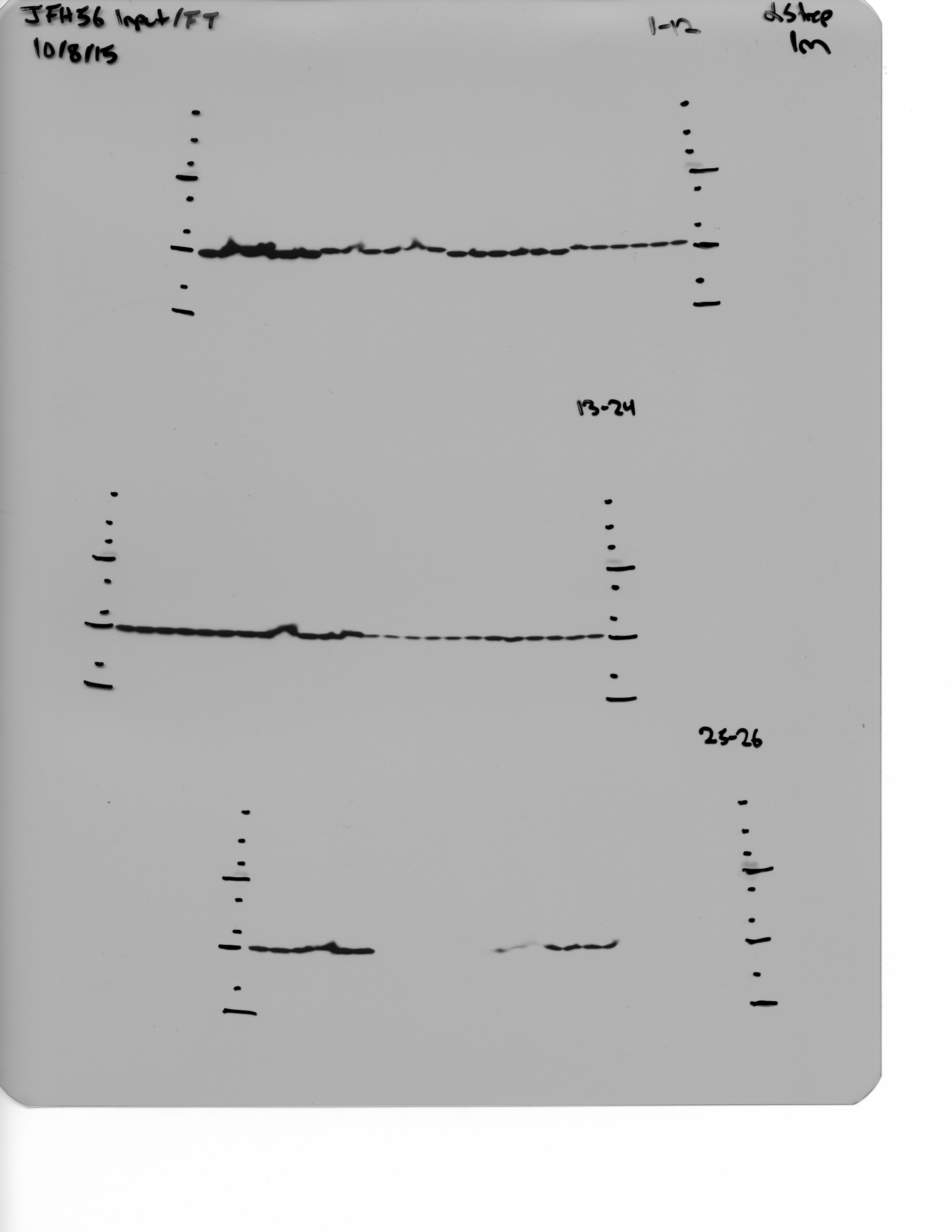

Supplement: Supplementary file 11 — Source Data [file 41467_2023_41442_MOESM11_ESM.zip › Haas_SourceData/Western Blot Scans (Supp Fig 3)/A549/JFH056 - Strep - 1m.tif]

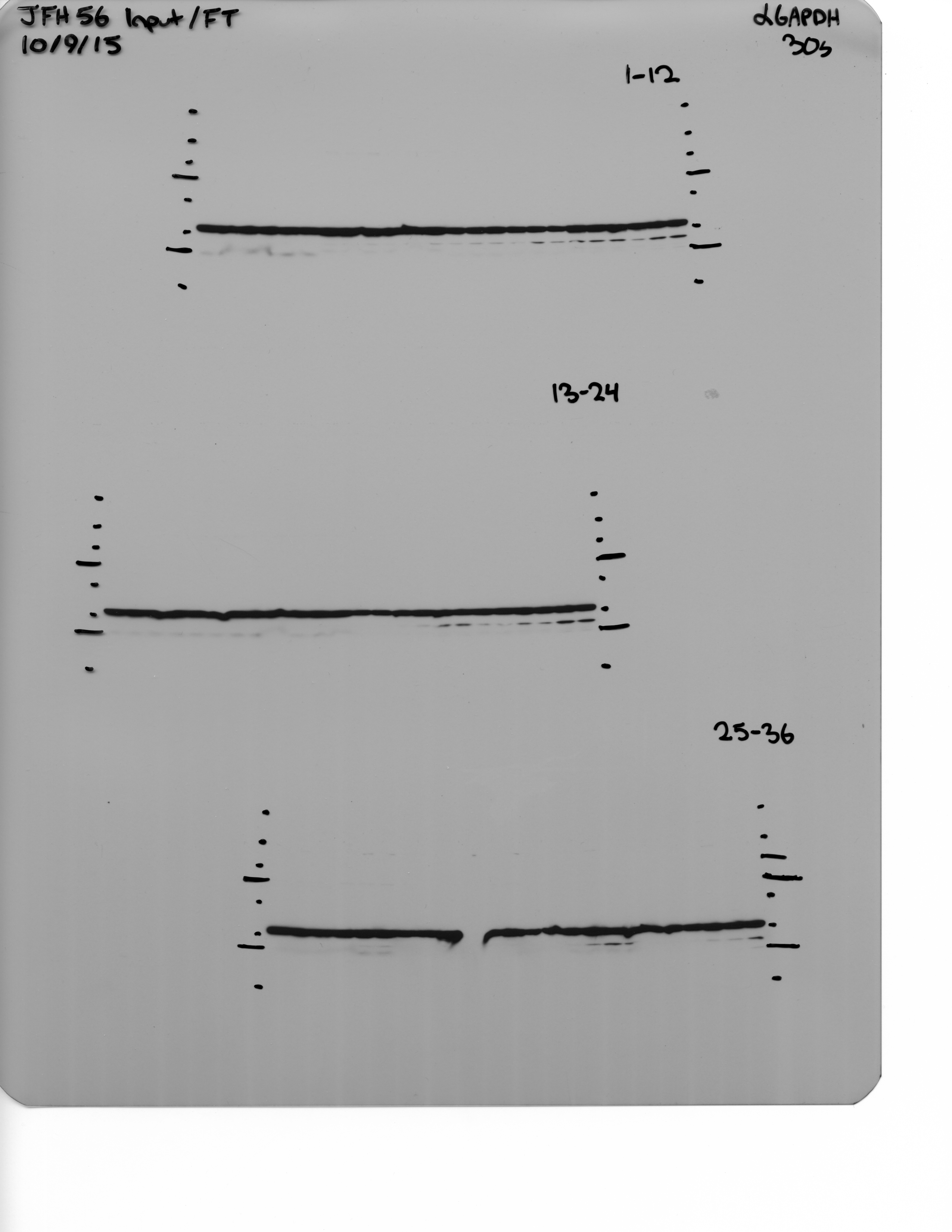

Supplement: Supplementary file 11 — Source Data [file 41467_2023_41442_MOESM11_ESM.zip › Haas_SourceData/Western Blot Scans (Supp Fig 3)/A549/JFH056 - GAPDH - 30s.tif]

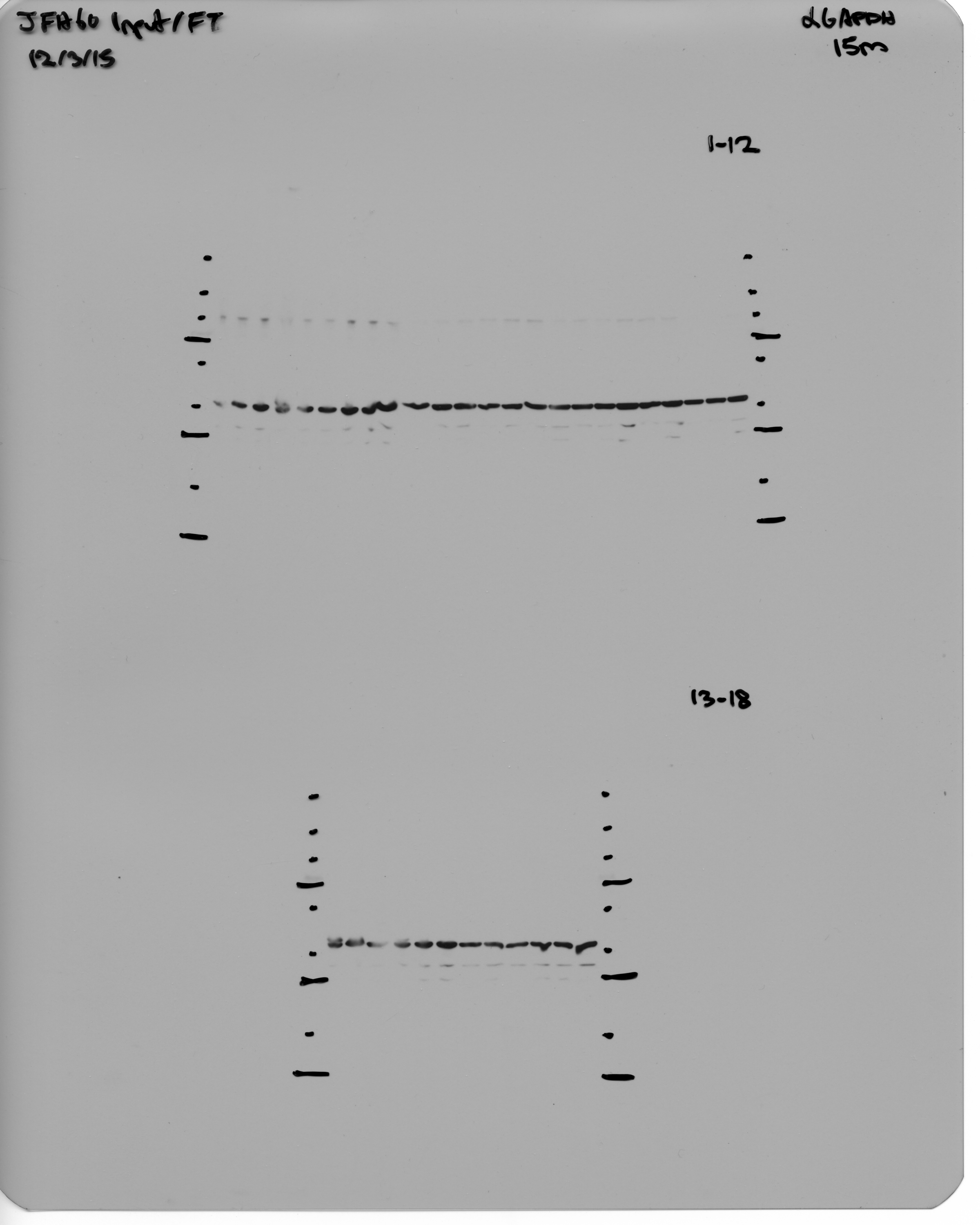

Supplement: Supplementary file 11 — Source Data [file 41467_2023_41442_MOESM11_ESM.zip › Haas_SourceData/Western Blot Scans (Supp Fig 3)/A549/JFH060 - GAPDH - 15m.tif]

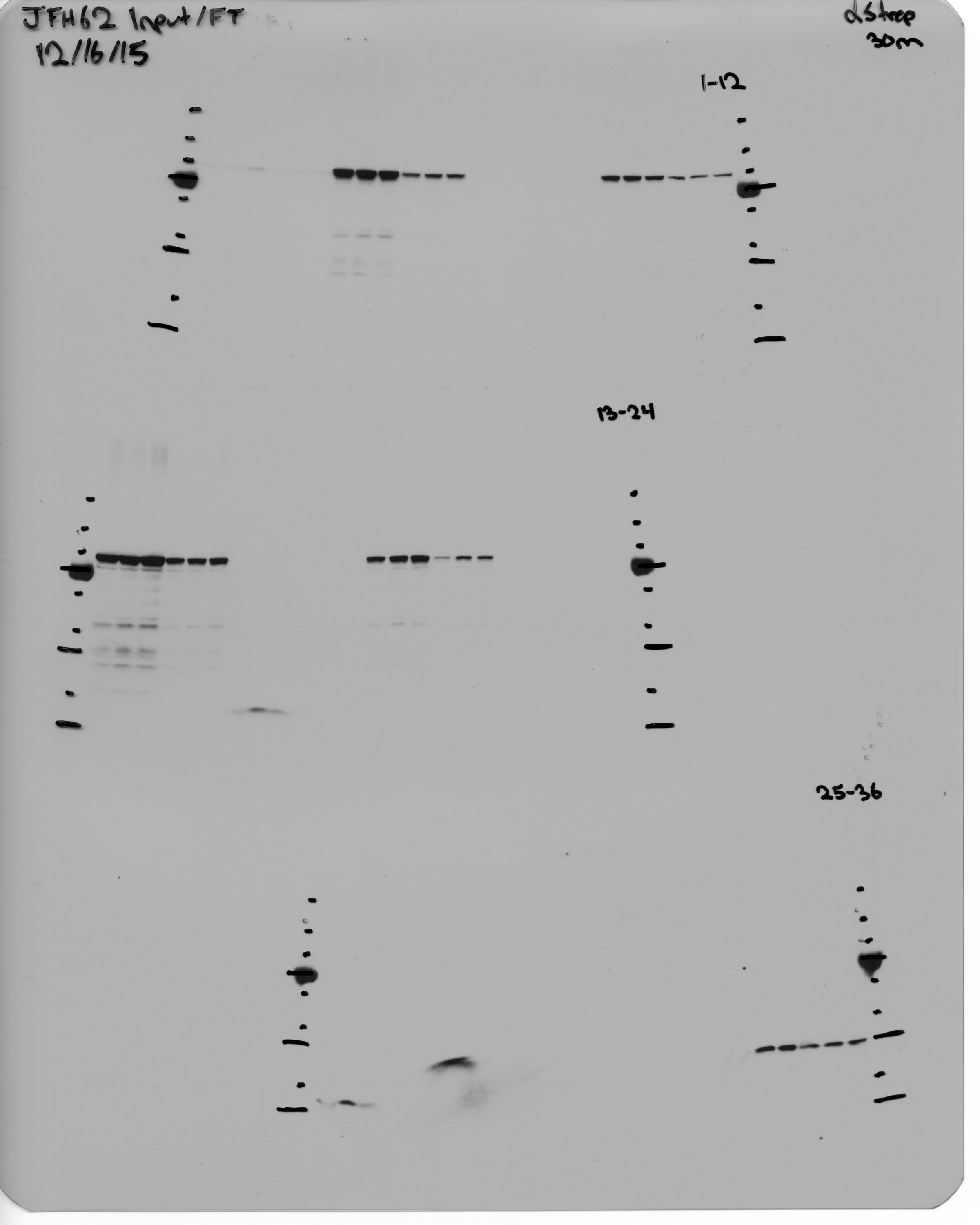

Supplement: Supplementary file 11 — Source Data [file 41467_2023_41442_MOESM11_ESM.zip › Haas_SourceData/Western Blot Scans (Supp Fig 3)/A549/JFH062 - Strep - 30m.tif]

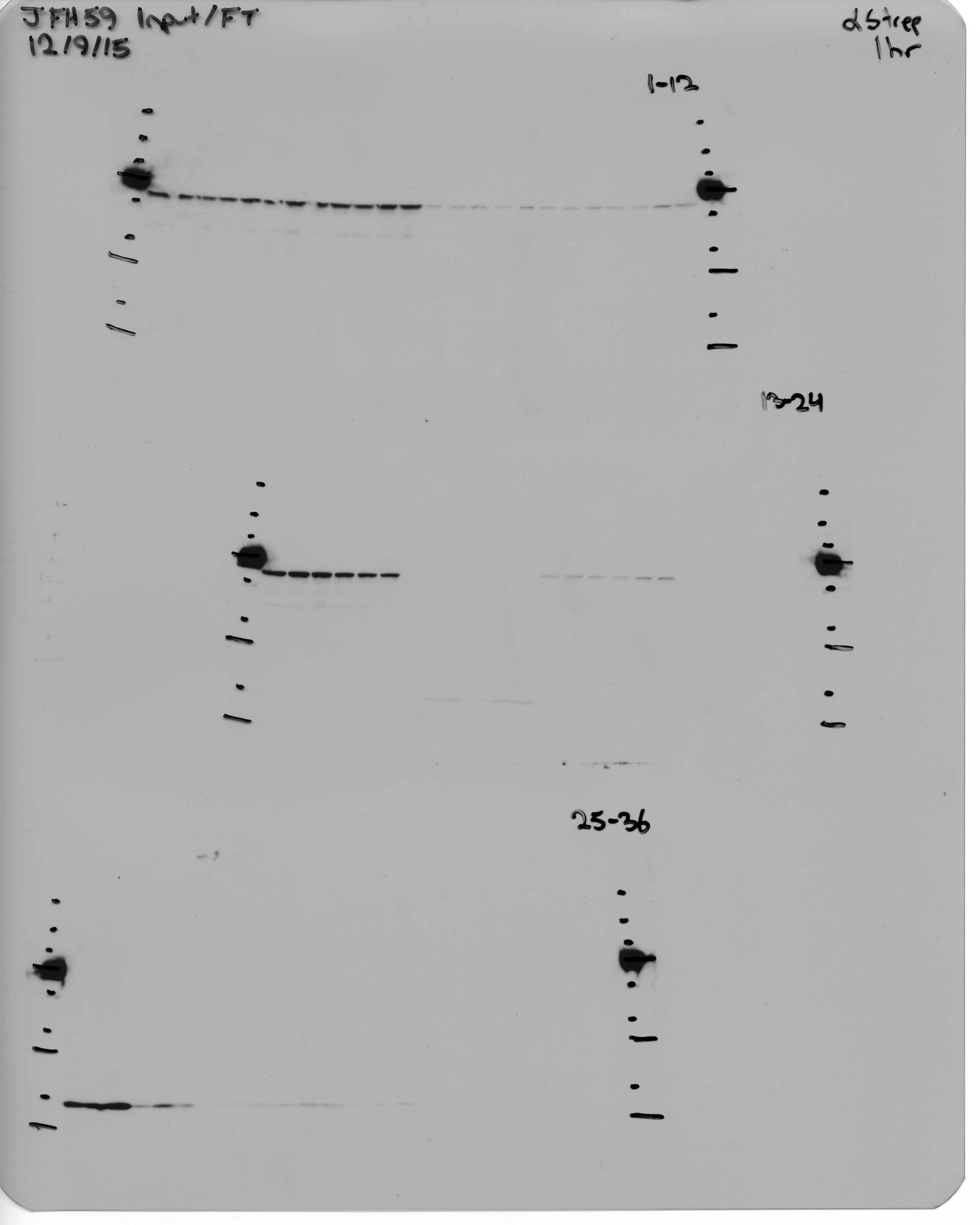

Supplement: Supplementary file 11 — Source Data [file 41467_2023_41442_MOESM11_ESM.zip › Haas_SourceData/Western Blot Scans (Supp Fig 3)/A549/JFH059 - Strep - 60m.tif]

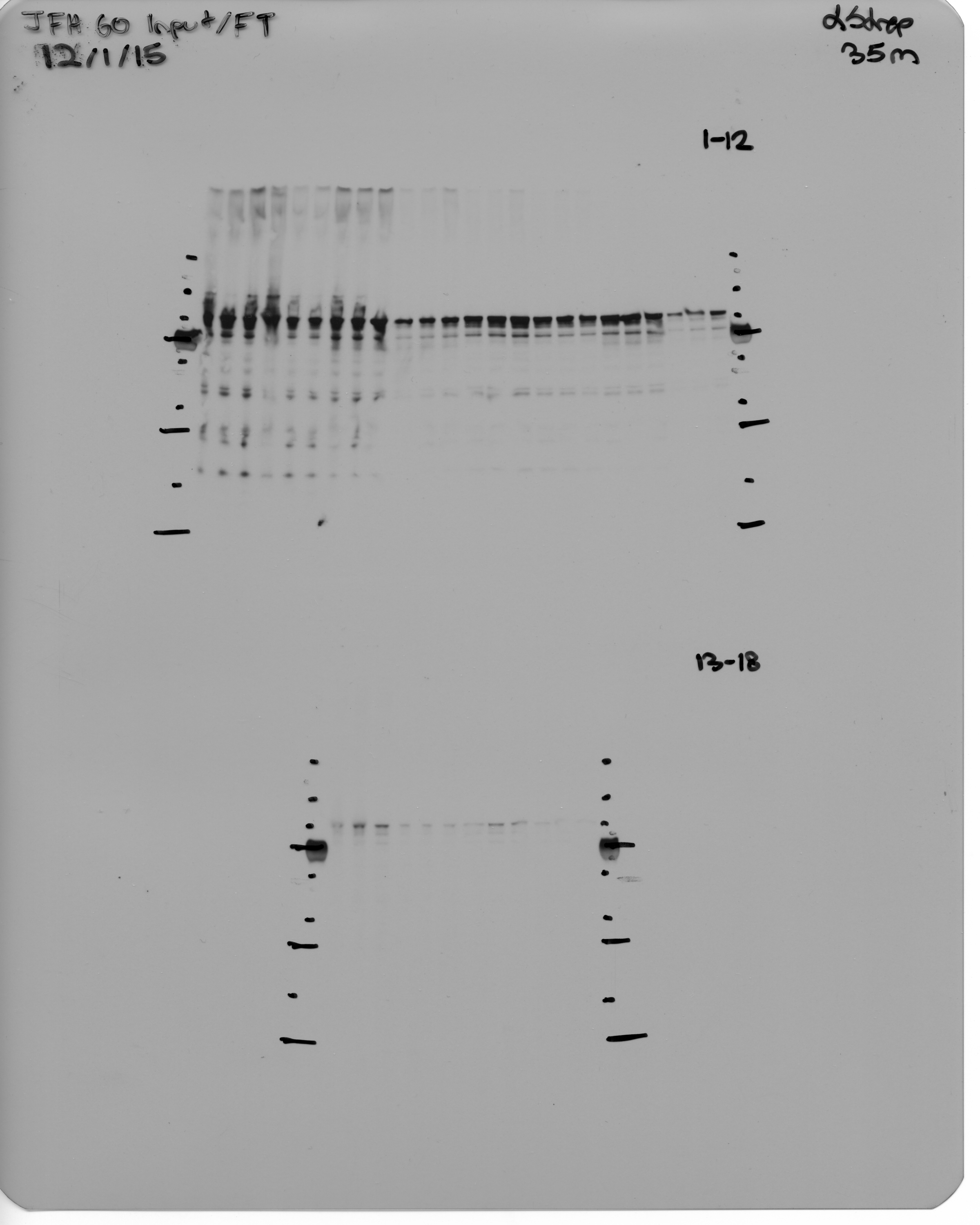

Supplement: Supplementary file 11 — Source Data [file 41467_2023_41442_MOESM11_ESM.zip › Haas_SourceData/Western Blot Scans (Supp Fig 3)/A549/JFH060 - Strep - 35m.tif]

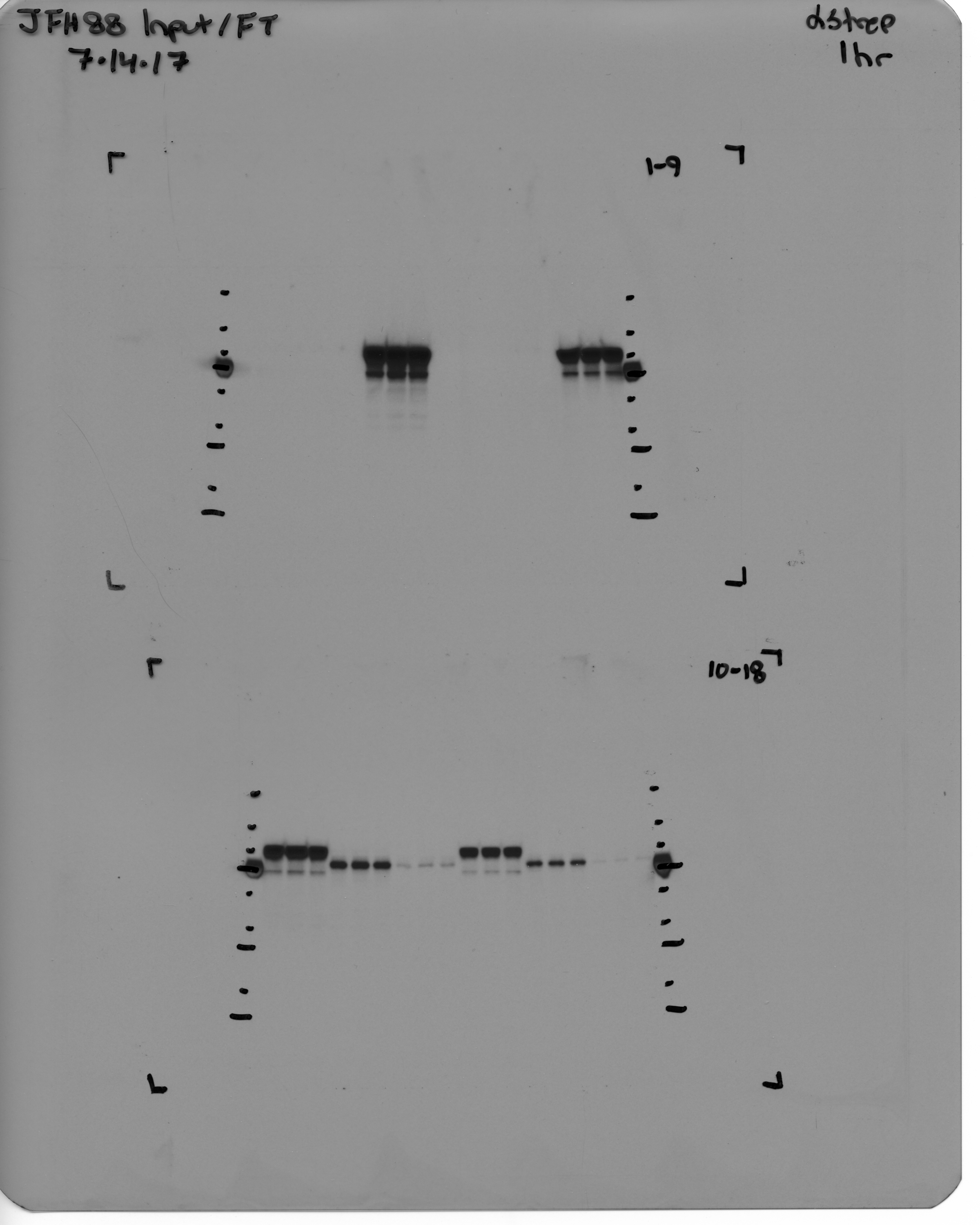

Supplement: Supplementary file 11 — Source Data [file 41467_2023_41442_MOESM11_ESM.zip › Haas_SourceData/Western Blot Scans (Supp Fig 3)/A549/JFH088 - Strep - 60m.tif]

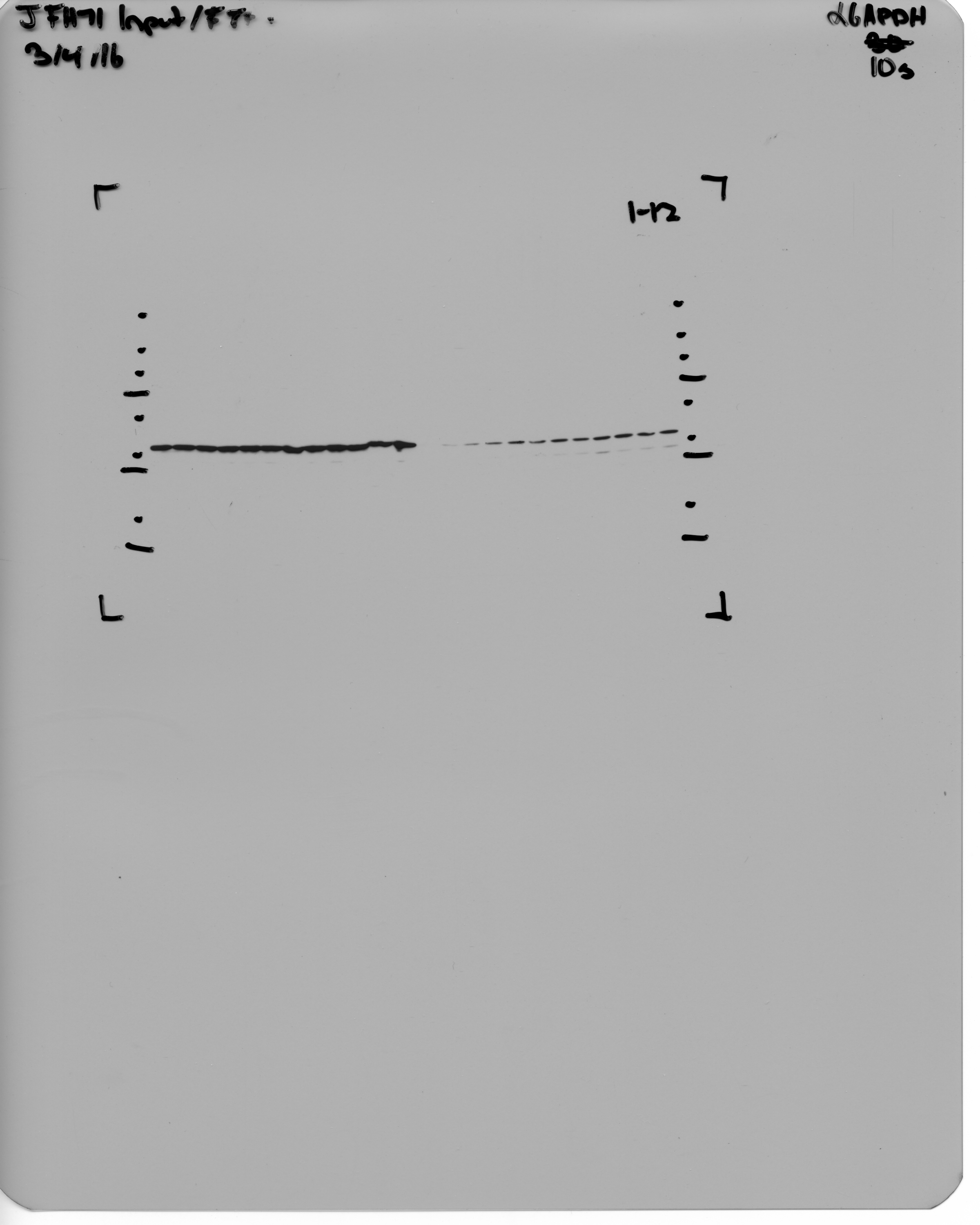

Supplement: Supplementary file 11 — Source Data [file 41467_2023_41442_MOESM11_ESM.zip › Haas_SourceData/Western Blot Scans (Supp Fig 3)/A549/JFH071 - GAPDH - 10s.tif]

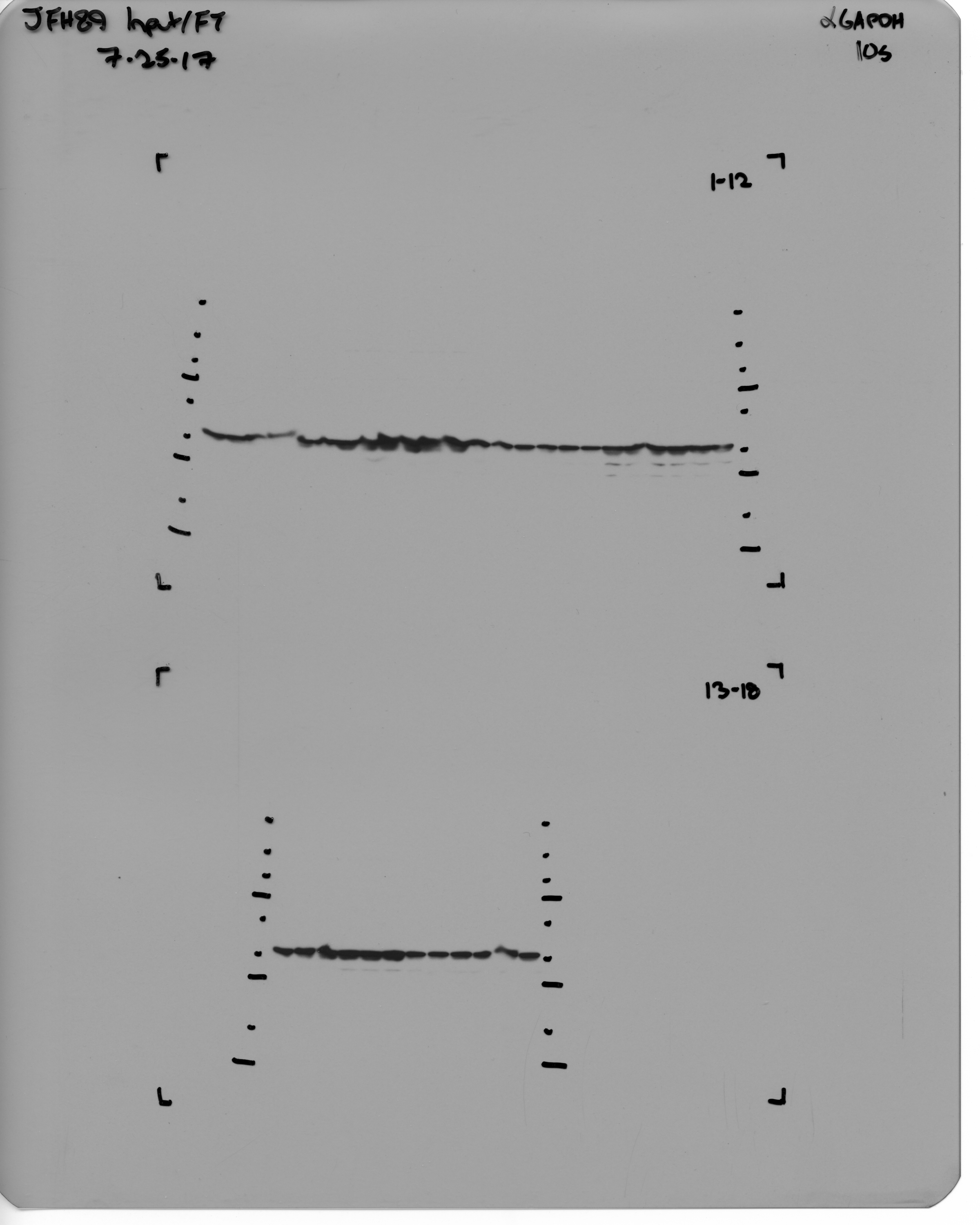

Supplement: Supplementary file 11 — Source Data [file 41467_2023_41442_MOESM11_ESM.zip › Haas_SourceData/Western Blot Scans (Supp Fig 3)/A549/JFH089 - GAPDH - 10s.tif]

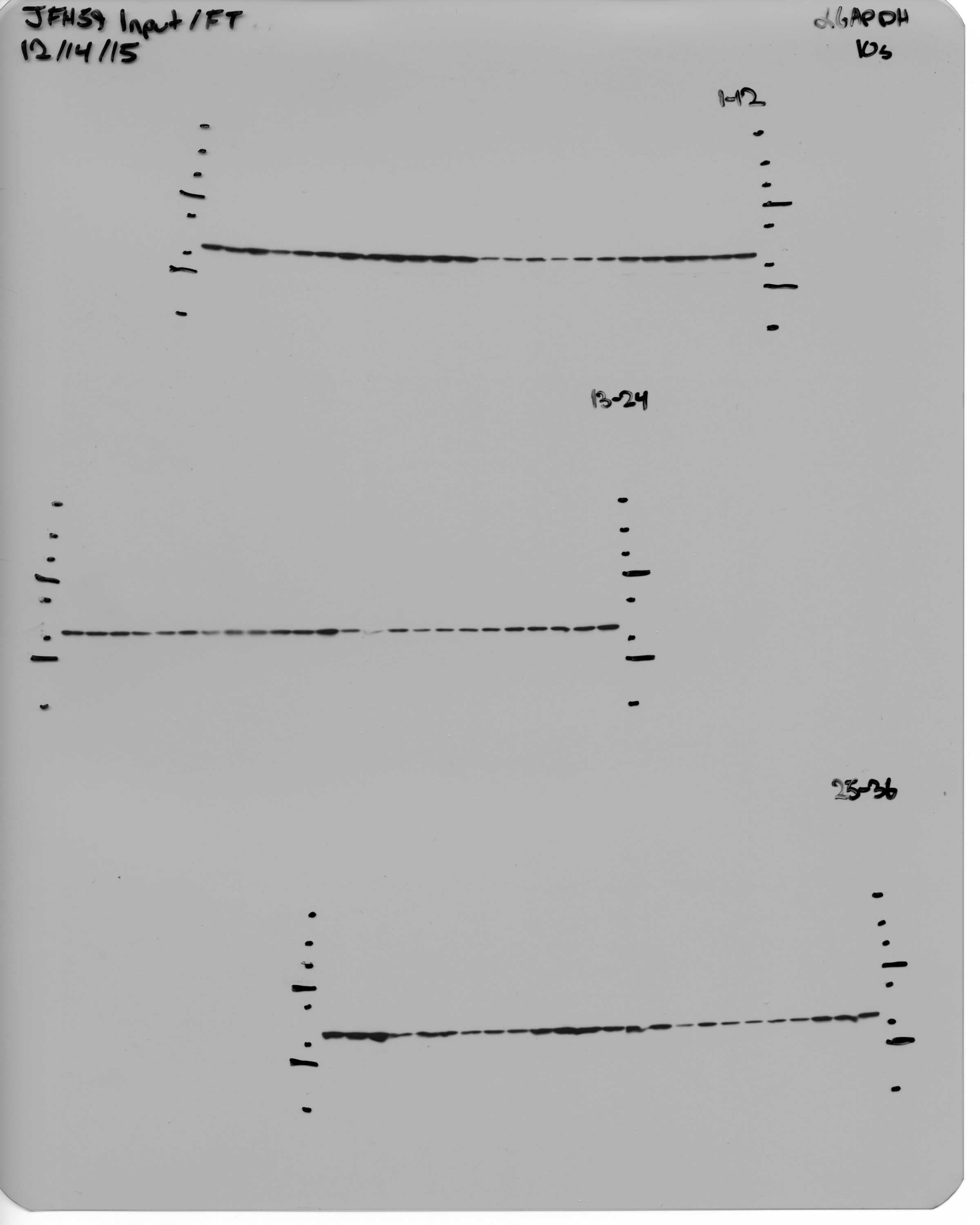

Supplement: Supplementary file 11 — Source Data [file 41467_2023_41442_MOESM11_ESM.zip › Haas_SourceData/Western Blot Scans (Supp Fig 3)/A549/JFH059 - GAPDH - 10s.tif]

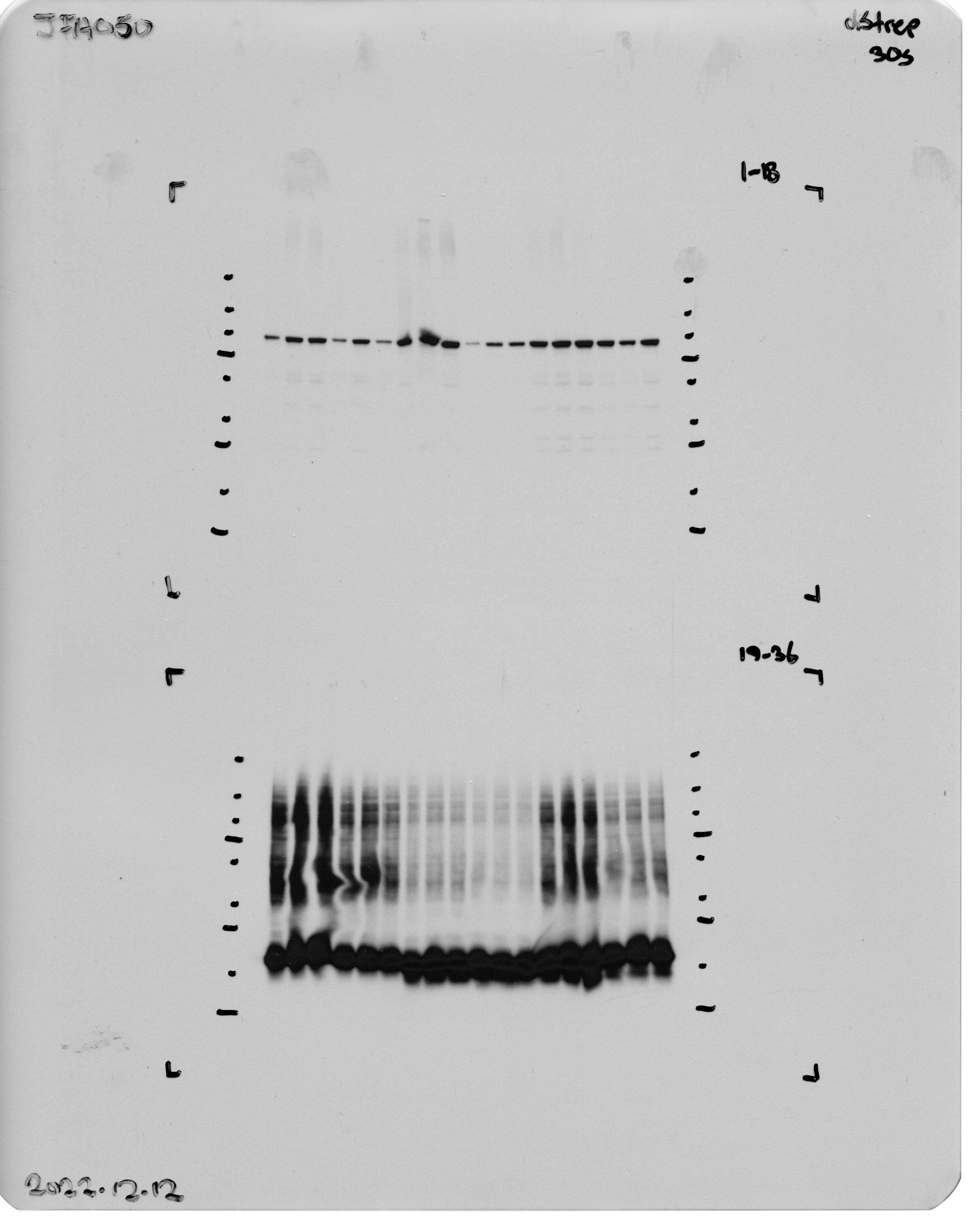

Supplement: Supplementary file 11 — Source Data [file 41467_2023_41442_MOESM11_ESM.zip › Haas_SourceData/Western Blot Scans (Supp Fig 3)/A549/JFH050 - Strep - 30s.tif]

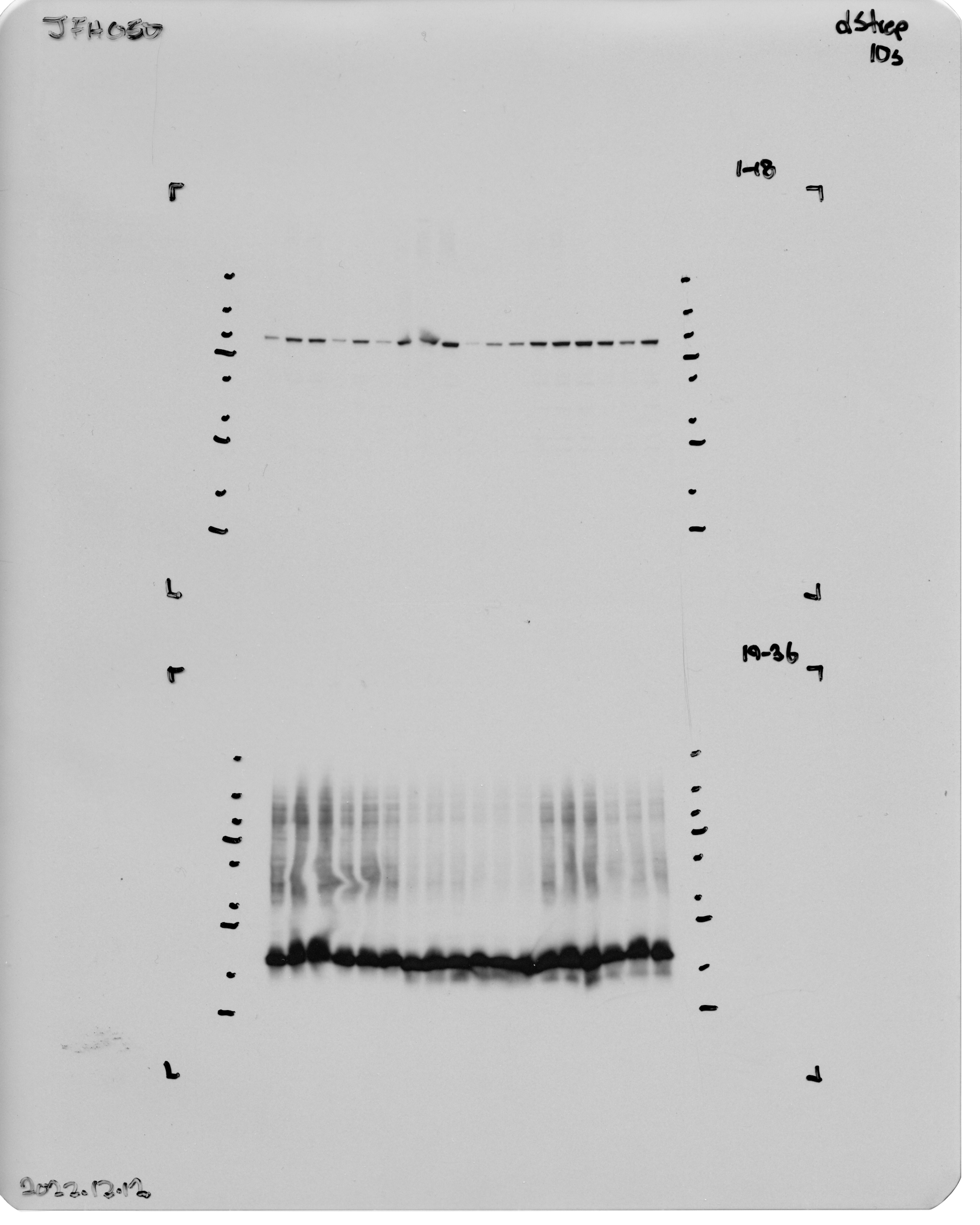

Supplement: Supplementary file 11 — Source Data [file 41467_2023_41442_MOESM11_ESM.zip › Haas_SourceData/Western Blot Scans (Supp Fig 3)/A549/JFH050 - Strep - 10s.tif]

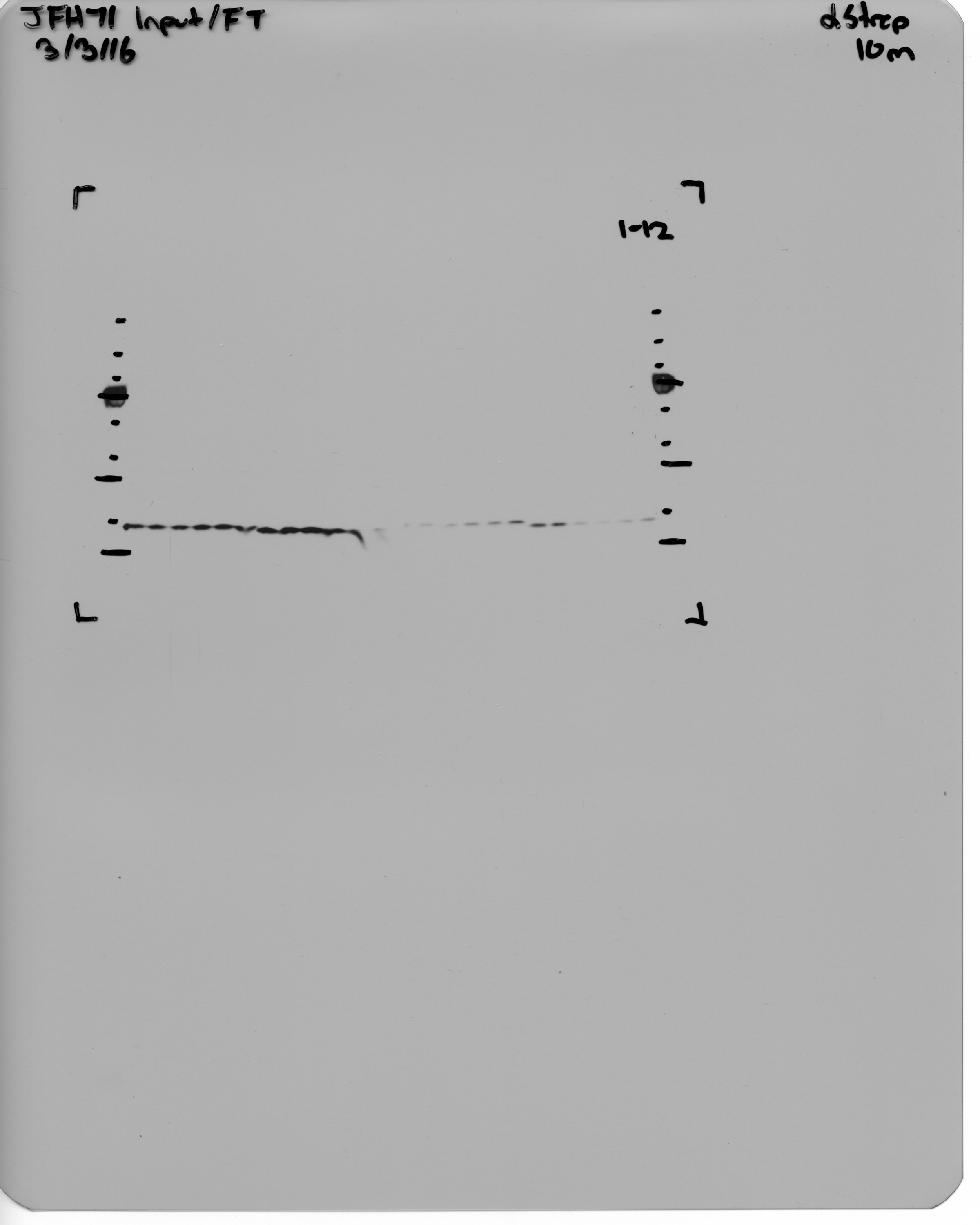

Supplement: Supplementary file 11 — Source Data [file 41467_2023_41442_MOESM11_ESM.zip › Haas_SourceData/Western Blot Scans (Supp Fig 3)/A549/JFH071 - Strep - 10m.tif]

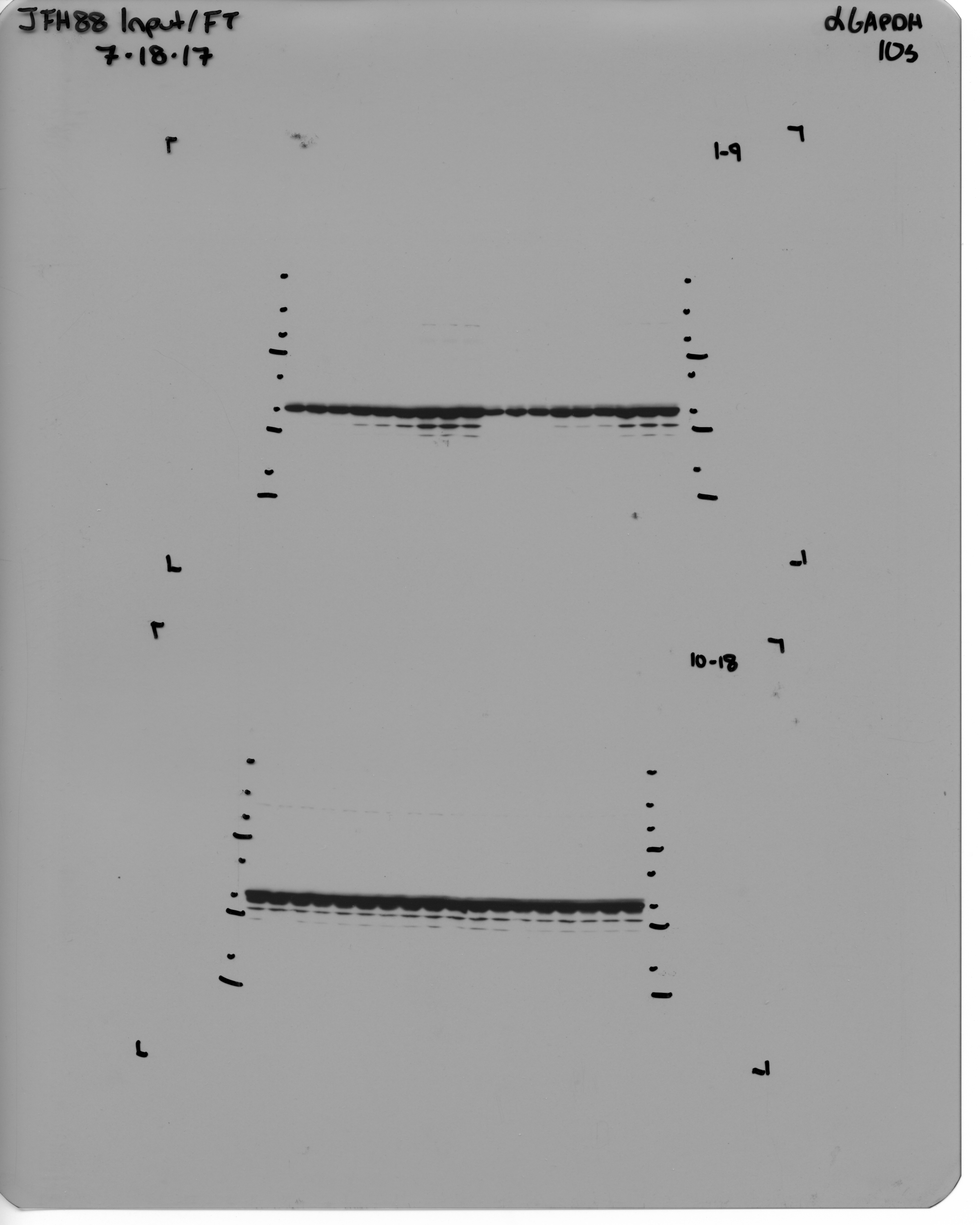

Supplement: Supplementary file 11 — Source Data [file 41467_2023_41442_MOESM11_ESM.zip › Haas_SourceData/Western Blot Scans (Supp Fig 3)/A549/JFH088 - GAPDH - 10s.tif]

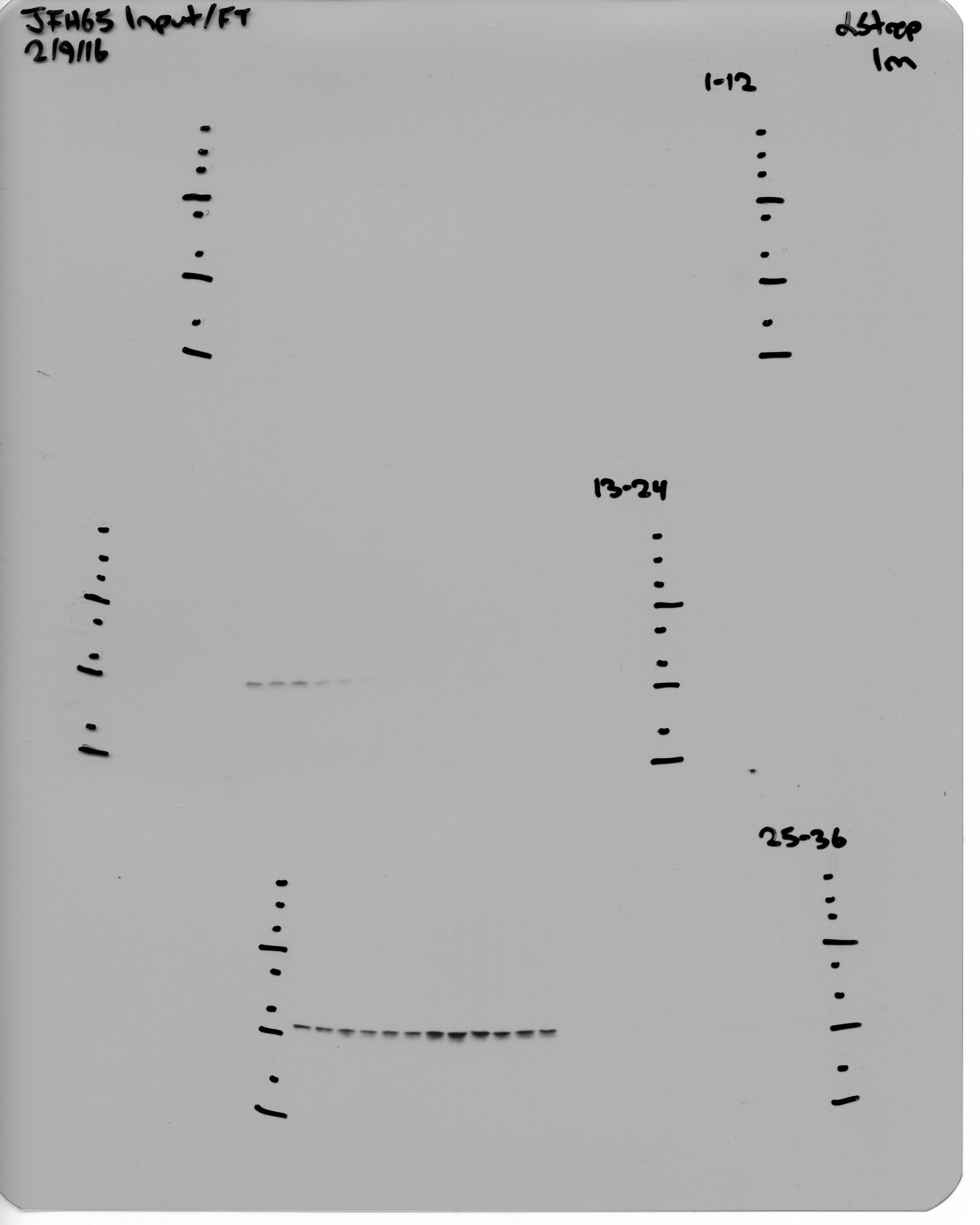

Supplement: Supplementary file 11 — Source Data [file 41467_2023_41442_MOESM11_ESM.zip › Haas_SourceData/Western Blot Scans (Supp Fig 3)/A549/JFH065 - Strep - 1m.tif]
